# Integrative Analysis of Cuproptosis‐Related Mitochondrial Depolarisation Genes for Prognostic Prediction in Non‐Small Cell Lung Cancer

**DOI:** 10.1111/jcmm.70438

**Published:** 2025-02-26

**Authors:** Guoqing Lyu, Lihua Dai, Xin Deng, Xiankai Liu, Yan Guo, Yuan Zhang, Xiufeng Wang, Yan Huang, Sun Wu, Jin‐Cheng Guo, Yanting Liu

**Affiliations:** ^1^ Department of Hematology The First Affiliated Hospital of Xinxiang Medical University Weihui Henan Province China; ^2^ Life Science Center The First Affiliated Hospital of Xinxiang Medical University Weihui Henan Province China; ^3^ Key Laboratory for Leukemia Molecular Diagnosis and Treatment in Xinxiang City Weihui Henan Province China; ^4^ Key Laboratory for Lymphoma Molecular Diagnosis and Treatment in Xinxiang City Weihui Henan Province China; ^5^ Prenatal Diagnosis Center Shanghai General Hospital Affiliated to Shanghai JiaoTong University School of Medicine Shanghai China; ^6^ Beijing University of Chinese Medicine Beijing China; ^7^ Department of Oncology The First Affiliated Hospital of Xinxiang Medical University Weihui Henan Province China

**Keywords:** cuproptosis, mitochondrial depolarisation, non‐small cell lung cancer, prognostic model, tumour marker

## Abstract

In this research, we conducted an in‐depth analysis of differentially expressed genes associated with mitochondrial depolarisation in non‐small cell lung cancer (NSCLC) using single‐cell sequencing. By combining our findings with cuproptosis‐related genes, we identified 10 significant risk genes: DCN, PTHLH, CRYAB, HMGCS1, DSG3, ZFP36L2, SCAND1, NUDT4, NDUFA4L2 and RPL36A, using univariate Cox regression analysis and machine learning methods. These genes form the core of our prognosis risk prediction model, which demonstrated high specificity and accuracy in predicting patient outcomes, as evidenced by ROC curve analysis. Kaplan–Meier curves further confirmed that patients in the low‐risk group had significantly better survival rates compared to those in the high‐risk group. Our models also provided valuable insights into the tumour microenvironment, immunotherapy sensitivity and chemotherapy response. To facilitate the quantification of the probability of patient survival, we incorporated clinical data into a nomogram. We comprehensively analysed the mutation status and expression patterns of the 10 risk genes using bulk transcriptomic, single‐cell and spatial transcriptomic datasets. Drug target predictions highlighted DSG3, PTHLH, ZFP36L2, DCN and NDUFA4L2 as promising therapeutic targets. Notably, RPL36A emerged as a potential tumour marker for NSCLC, with its expression validated in lung cancer cell lines through qPCR. This study has established a predictive models based on mitochondrial depolarisation genes associated with cuproptosis, aiding clinicians in forecasting overall survival and guiding personalised treatment strategies. The identification of novel tumour markers has paved the way for targeted therapies, and therapeutic targets are critical for advancing the treatment of NSCLC.

## 1 Introduction

Lung cancer is the most frequently diagnosed malignancy, with approximately 2.5 million new cases reported annually, accounting for 12.4% of all global cancer diagnoses. It is also the foremost cause of cancer‐related mortality, responsible for an estimated 1.8 million deaths (18.7%) per year [1]. Among these, non‐small cell lung cancer (NSCLC) constitutes more than 80% of all lung cancer cases. NSCLC often manifests at advanced stages, which limits surgical intervention and negatively impacts patient survival rates [2]. The delayed onset of symptoms and the absence of effective early detection methods further exacerbate the challenges in predicting prognosis and limit therapeutic options, thereby affecting survival outcomes [3]. The objective of this study was to develop a predictive model for the prognosis of NSCLC patients, aiming to forecast their survival and to elucidate the role of cuproptosis‐associated mitochondrial depolarisation genes in the progression of NSCLC.

Mitochondrial function is crucial for cell survival [4]. Under normal conditions, an electrochemical gradient of hydrogen ions (H+) is established across the inner mitochondrial membrane as electrons traverse the respiratory chain, driving ATP synthesis. However, the production of reactive oxygen species (ROS) can induce the opening of permeability transition pores (PTPs) in the mitochondrial membrane, leading to mitochondrial permeability transition (MPT) [5, 6, 7]. This MPT results in the collapse and depolarisation of the mitochondrial membrane potential, causing ATP depletion [8]. Mitochondrial depolarisation can lead to necrosis, cytochrome c release, and the rupture of the outer mitochondrial membrane. Severe ATP depletion typically results in necrosis, whereas moderate ATP depletion triggers apoptosis [9, 10].

Tsvetkov et al. identified a novel form of cell death known as cuproptosis, detailing its underlying mechanism [11]. Copper toxicity can be modulated by regulating intracellular copper ion concentrations, a process that holds potential for cancer therapy [12]. Future research on copper toxicity‐based treatments should focus on identifying patient populations that could benefit from these therapies, particularly those with cancers characterised by high mitochondrial metabolism, tumour stem cell properties and drug resistance [13]. These studies aim to provide new therapeutic strategies for these challenging cancers.

Both mitochondrial depolarisation and cuproptosis hold significant promise for tumour therapy, making it essential to further explore their interactions. In this study, we examined cuproptosis‐related genes associated with mitochondrial depolarisation in NSCLC using both single‐cell and bulk transcriptomic data. We developed a prognosis prediction model that demonstrated high accuracy. Notable differences in immune infiltration and responses to immunotherapy and chemotherapy were observed between high‐ and low‐risk patients. Additionally, we analysed 10 risk genes at various transcriptome levels to determine their expression and mutation patterns, which will inform drug predictions for precision medicine and personalised therapy.

## 2 Materials and Methods

### 2.1 Data Source

The data for this study were obtained from the TCGA, GEO, ICGC and BioStudies databases. The expression data and patient prognosis data for the model training set were derived from TCGA‐LUAD and TCGA‐LUSC (*n* = 1026), while the validation set data were from GSE30219 (*n* = 307). We performed relevant pathway scoring and differential gene analysis using a single‐cell dataset of NSCLC from the GEO database (GSE117570, *n* = 8). For subsequent validation, we used additional datasets from the GEO database (GSE131907, *n* = 11). The prediction model was also used to predict the prognosis of patients with other types of cancer, utilising datasets containing expression data and prognostic data of patients, including gastric cancer (GSE84437, *n* = 433), liver cancer (ICGC‐Liver Cancer—RIKEN, JP, *n* = 208), ovarian cancer (GSE63885, *n* = 70), urothelial carcinoma (GSE32894, *n* = 224), pancreatic cancer (GSE85916, *n* = 79), breast cancer (GSE20685, *n* = 327) and colorectal cancer (GSE17537, *n* = 55). Publicly available spatial transcriptome (ST) data can be found in the BioStudies database under accession number E‐MTAB‐13530 (*n* = 1).

### 2.2 Data Analysis

Single‐cell or spatial transcriptome data processing and analysis.

The initial gene expression matrix was preprocessed using the ‘Seurat’ R package (version 4.4.0). Cells were filtered to retain those with more than 300 expressed genes, more than 3 non‐zero UMI counts across cells, < 50% mitochondrial UMI content, more than 3% ribosomal protein content and < 0.1% haemoglobin content. Genes were retained if expressed in at least three cells. A set of highly variable genes was selected for principal component analysis (PCA), and the top 30 significant principal components were chosen for Uniform Manifold Approximation and Projection (UMAP) dimension reduction to visualise gene expression patterns. To correct for batch effects, the Harmony methods were applied. Differentially expressed genes within each cell subpopulation were identified using the FindAllMarkers function with the Wilcoxon rank‐sum test and FDR correction for multiple hypothesis testing. Cellular annotation was achieved through clustering using the FindClusters function with a resolution parameter of 0.8, and further refined using the ‘SingleR’ package, which aligns single‐cell RNA sequencing data with reference datasets to assign cell types. Cell cycle heterogeneity and pathway scores were assessed using the AUCell algorithm. Differential analysis identified 560 differentially expressed genes (DEGs) in subpopulations of epithelial cells, which were combined with 19 cuproptosis‐related genes (CRGs) from the literature and correlated with 34 mitochondrial depolarisation‐related genes (MDRGs) from the MSigDB database, resulting in a final total of 345 cuproptosis‐related mitochondrial depolarisation genes (CRMDGs). Spatial transcriptome data were analysed using the ‘spacexr’ software package to associate cellular subpopulation annotations with spatial locations [14]. The Scissor algorithm, based on the ‘scAB’ R package, was utilised to identify cell subpopulations highly associated with NSCLC by integrating single‐cell data, bulk RNA‐seq data and phenotypic information [15].

### 2.3 Construction and Evaluation of Predictive Models

The initial set of 345 cuproptosis‐related mitochondrial depolarisation genes (CRMDGs) was narrowed down to 49 through univariate Cox regression analysis. Following this, the Lasso machine learning algorithm identified 10 risk genes. The risk score was calculated using the formula: Riskscore = ∑(Expi*coefi). Survival analysis was conducted using the Kaplan–Meier method and log‐rank test to evaluate survival rates between the two patient groups. The ‘survival’ and ‘survminer’ packages were used for univariate Cox regression, multivariate Cox regression construction, survival analysis, and forest plot visualisation. To evaluate the predictive performance of the prognostic model and the nomogram, we utilised R packages such as ‘rms’, ‘pROC’, ‘timeROC’ and ‘dcurves’ to perform ROC curve analysis, calibration curves and clinical decision curve analysis (DCA). These analyses aimed to compare the prediction accuracy and clinical utility of the Lasso regression model, the nomogram and clinical data. The list of related genes appearing in the article is provided in Table S1.

### 2.4 Application of Prognostic Modelling

Patients were divided into high‐risk and low‐risk groups based on their risk scores. To compare the differential gene expression between these groups, we used the ‘limma’ package. Genes with a *p* < 0.05 and logFC < −1 were considered downregulated, while genes with a *p* < 0.05 and logFC > 1 were considered upregulated. The “clusterProfiler” package was then used to perform Gene Ontology (GO), Kyoto Encyclopedia of Genes and Genomes (KEGG) and Gene Set Enrichment Analysis (GSEA) pathway enrichment analyses on the differentially expressed genes. The ‘GVSA’ software package was utilised to identify distinct enrichment pathways between these groups. Kaplan–Meier survival analysis was used to evaluate the survival differences between high‐risk and low‐risk groups of patients in NSCLC and other tumour types after risk prediction. To evaluate differences in immune and stromal cell infiltration, we employed the ‘ssGSEA’, ‘MCPcounter’ and ‘quanTIseq’ software packages [16, 17, 18]. We also accessed the Tumor Immune Dysfunction and Exclusion (TIDE) database to predict treatment responsiveness in each group [19]. Additionally, the ‘oncoPredict’ software package was used to predict drug sensitivity based on gene expression status, which helped us study the correlation between high‐risk and low‐risk groups and their sensitivity to chemotherapeutic agents [20].

### 2.5 Expression and Mutation Patterns of Risk Genes

Somatic mutation analysis was conducted using the ‘maftools’ software package to explore and visualise mutation patterns of risk genes linked to NSCLC [21]. The expression levels of these 10 risk genes in normal tissues, lung cancer tissues and various cellular subpopulations were evaluated using bulk transcriptomics, single‐cell sequencing and spatial transcriptomics data to assess their roles and prognostic significance. The GEPIA2 database was utilised to assess the prognostic impact of risk genes in NSCLC [22]. Meanwhile, the UALCAN database was employed to explore variations in RPL36A gene expression among patients with different stages of NSCLC [23]. Additionally, the HPA database provided immunohistochemical images illustrating the expression levels of these risk genes in both normal lung tissue and lung cancer tissue. The DGIdb database was used to perform drug predictions for several risk genes, aiding in the discovery of potential therapeutic targets for NSCLC [24].

### 2.6 Validation Using Quantitative Real‐Time PCR (qPCR)

To validate the expression of drug target genes among several prognostic model genes, qPCR was performed on the normal lung cell line BEAS‐2B and the lung cancer cell lines H1703 and H226. Total RNA was extracted from all cell lines using the RNAprep Pure Cell/Bacteria Kit (TIANGEN, Beijing, China; Cat. No. DP430). Reverse transcription was carried out using the PrimeScript RT Reagent Kit (Takara, Beijing, China; Cat. No. RR037A). For the qPCR, SYBR Green qPCR Mix (Beyotime, Beijing, China; Cat. No. D7260) was utilised, with GAPDH serving as the internal reference for gene detection. The PCR conditions included a 2‐min pre‐denaturation step at 95°C, followed by 40 cycles of denaturation at 95°C for 15 s, annealing at 60°C for 30 s and extension at 60°C for 30 s. Primer sequences are detailed in Table S2. This study included three biological replicates, and the expression levels of biomarkers in the BEAS‐2B, H1703 and H226 cell lines were analysed using one‐way ANOVA, with a *p* < 0.05 considered statistically significant.

### 2.7 Statistical Analysis

Data manipulation, statistical analyses and visualisation processes were conducted using R software version 4.2.3. Overall survival (OS) of patients in different risk groups was estimated and compared using the Kaplan–Meier method and the log‐rank test. Discrepancies in continuous variables between the low‐risk and high‐risk groups were assessed using the Wilcoxon test or *t*‐test, depending on the data distribution. Categorical variables were evaluated using the chi‐squared test or Fisher's exact test. To control for multiple comparisons, *p*‐values were corrected using the false discovery rate (FDR) method. Relationships among variables were explored using Spearman's rank correlation coefficient. A *p*‐value threshold of < 0.05 was considered statistically significant.

## 3 Results

### 3.1 Screening Genes Involved in Mitochondrial Depolarisation Linked to Cuproptosis

**FIGURE 1:**
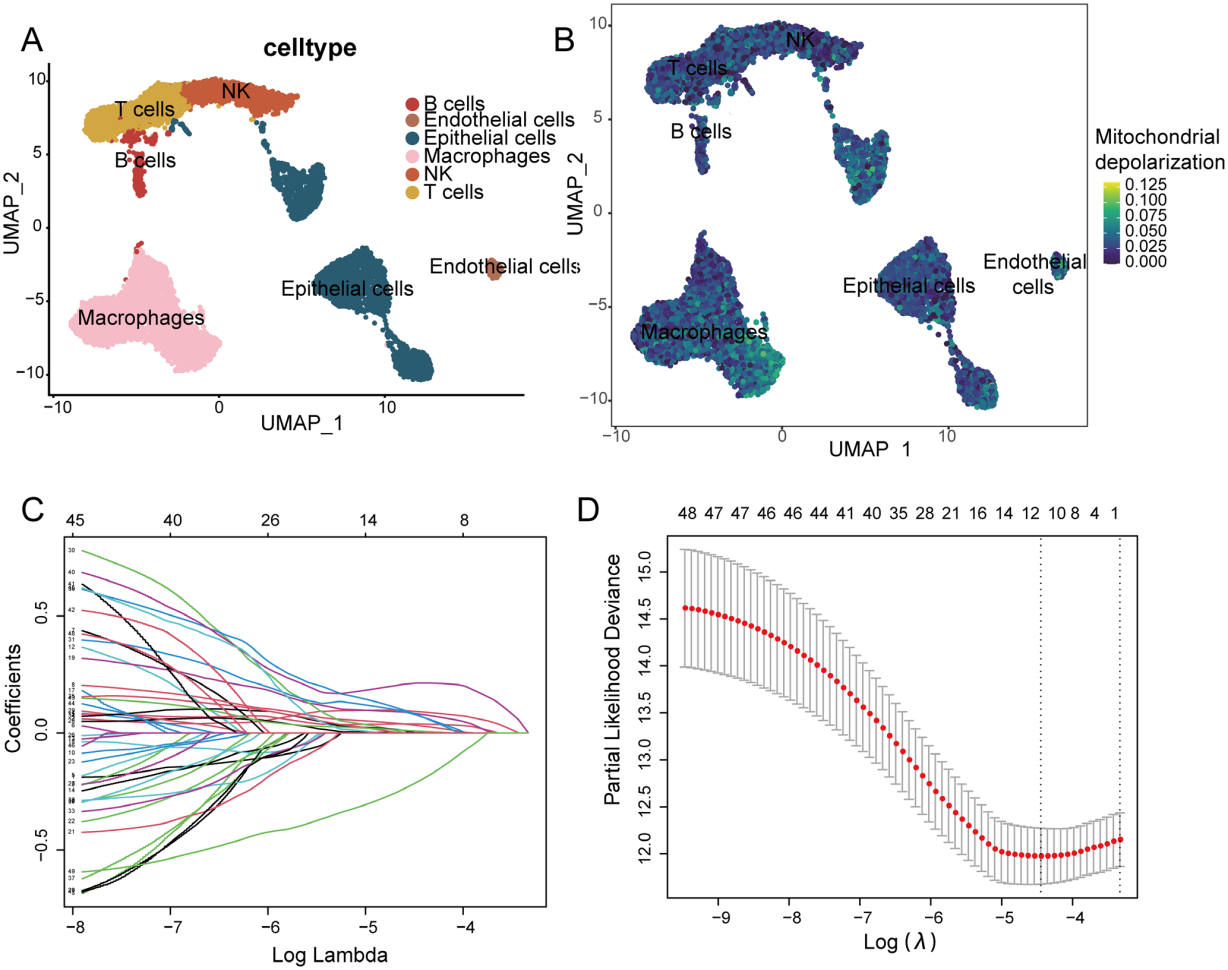
Screening genes involved in mitochondrial depolarisation linked to cuproptosis. (A) The UMAP plot shows the clustering pattern of NSCLC single‐cell data by cell type, with different colours representing different cell types. (B) Each point in the graph represents a cellular subpopulation, and the colour gradient indicates the mitochondrial depolarisation pathway AUCell score, from low (blue) to high (yellow). (C) The figure shows the coefficient path of the Lasso model, with the horizontal axis being the logarithmic scale of the Lasso regularization parameters and the vertical axis being the model coefficients. Different coloured lines represent different genes, and the larger the absolute value of the coefficient, the higher the importance of the gene in the model. (D) The figure illustrates the cross‐validation error of the Lasso model, with the logarithmic scale of the Lasso regularization parameters on the horizontal axis and the cross‐validation error on the vertical axis. The red dashed line indicates the optimal regularization parameter value, corresponding to the best model complexity and prediction performance.

Non‐small cell lung cancer single cell dataset GSE117570 downloaded from the GEO database. After quality control and normalisation, the dataset included 11,444 cells and 10,147 genes. After automated annotation of relevant cell subpopulations, we identified a total of six cell subpopulations, including B cells, T cells, endothelial cells, epithelial cells, macrophages and NK cells (Figure 1A). After evaluating the mitochondrial depolarisation pathway and dividing them into high and low mitochondrial polarisation groups based on the median threshold, 560 DEGs were identified in epithelial cell subpopulations (Figure 1B). To further investigate mitochondrial depolarisation, we obtained gene sets from the MSigDB dataset. Due to the limited sample size of the single‐cell data, we performed a correlation analysis between CRGs, DEGs and mitochondrial depolarisation genes in MSigDB using the TCGA‐LUAD and TCGA‐LUSC datasets, and found 345 CRMDGs |cor| > 0.2 and *p* < 0.05 (Table S3). Forty‐nine of these genes were found to be significant (*p* < 0.05) by univariate Cox regression (Table S4). These genes were then refined using the Lasso machine learning algorithm, resulting in the identification of 10 risk genes for NSCLC: DCN, PTHLH, CRYAB, HMGCS1, DSG3, ZFP36L2, SCAND1, NUDT4, NDUFA4L2 and RPL36A (Figure 1C,D) (Table S5).

### 3.2 Risk Modelling and Evaluation

**FIGURE 2:**
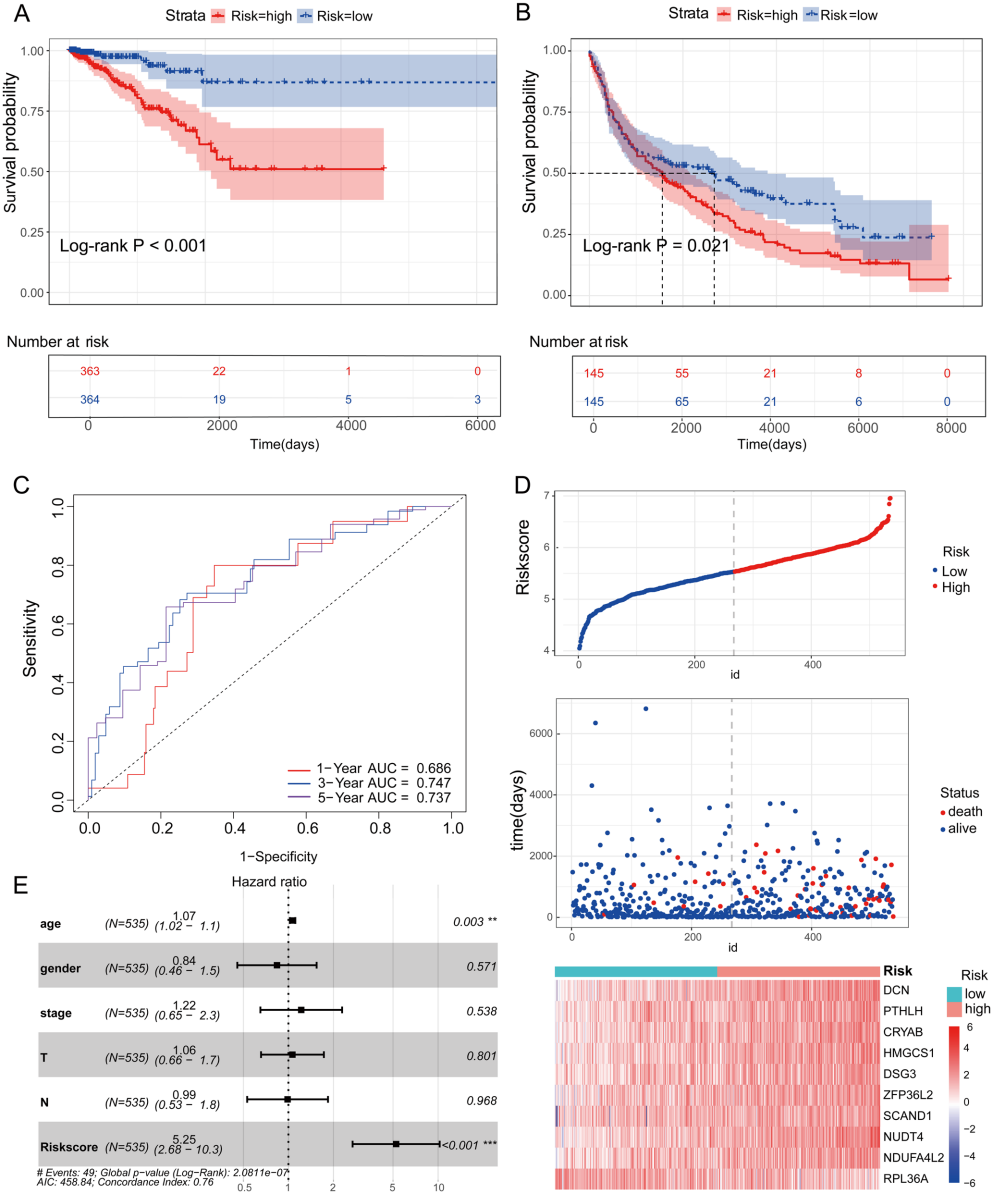
Evaluation of the risk model. (A) The results of the training set survival analysis demonstrate the change in survival probability over time for the two groups of patients (high‐risk and low‐risk groups). There is a significant difference in survival probability between the high‐risk group (red) and the low‐risk group (blue) (*p* < 0.0001). (B) The results of the validation set survival analysis demonstrate the probability of survival over time for the two groups of patients. There was a significant difference in survival probability between the high‐risk and low‐risk groups (*p* < 0.05). (C) ROC curves were used to assess the accuracy of the survival prediction model, with AUC (area under the curve) values of 0.686, 0.747 and 0.737 at 1, 3 and 5 years, respectively. (D) The top graph shows the distribution of risk scores for different patients, with a low‐risk group (blue) and a high‐risk group (red). The middle figure is a scatterplot showing the relationship between patients' survival status (dead or alive) and their risk scores. The lower panel shows the expression of several risk genes in patients in different risk groups. (E) The results showed the association between various clinical characteristics, such as age, sex, stage, T‐stage, N‐stage and risk score, and the probability of survival. Risk score was significantly associated with survival probability (*p* < 0.001), suggesting that risk score is an independent prognostic factor.

The risk score was calculated using the formula: Riskscore = (0.0910 * expression of DCN) + (0.0312 * expression of PTHLH) + (0.0388 * expression of CRYAB) + (0.0809 * expression of HMGCS1) + (0.0008 * expression of DSG3) + (0.0722 * expression of ZFP36L2) + (0.0742 * expression of SCAND1) + (0.2135 * expression of NUDT4) + (0.0075 * expression of NDUFA4L2) + (−0.2044 * expression of RPL36A). Patients were categorised into high‐ and low‐risk groups using the median risk score as the threshold. Kaplan–Meier survival analysis showed a significant difference in survival between these two groups in the TCGA dataset (*p* < 0.05) (Figure 2A). This significant difference in survival was also verified in the test set (*p* < 0.05) (Figure 2B). ROC curve analysis showed that the area under the curve for risk scores at 1, 3 and 5 years was 0.686, 0.747 and 0.737 (Figure 2C). Risk factor distribution maps showed the distribution of patients in different risk categories (Figure 2D). Furthermore, multivariable Cox regression analysis showed that risk score was an independent predictor of survival compared with other clinical information (Figure 2E).

### 3.3 Enrichment Analysis and Somatic Mutation Comparison

**FIGURE 3:**
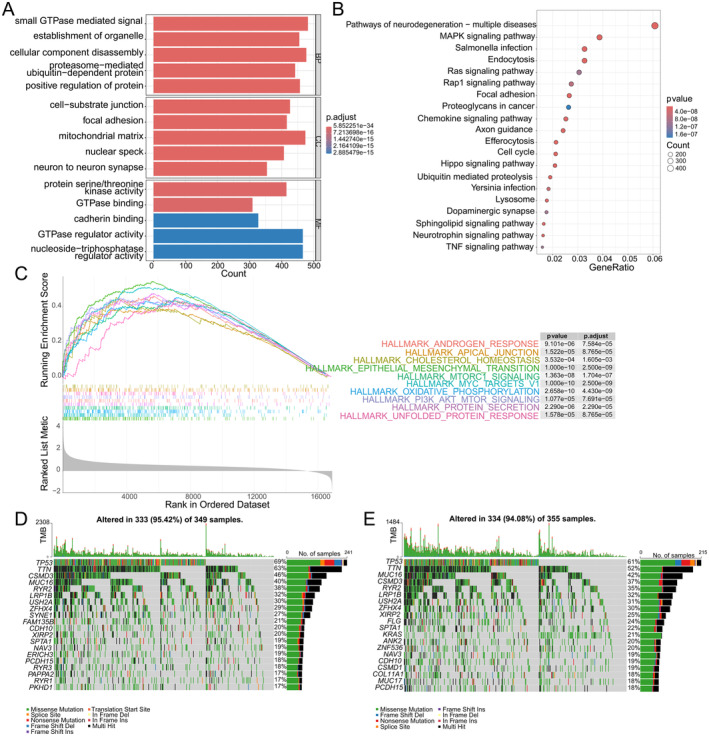
Results of enrichment analysis and somatic mutation comparison based on risk scores. (A) GO enrichment analysis bar graph showing biological processes, cellular components and molecular functions that are significantly enriched under specific conditions. The length of the bars indicates the number of genes enriched, and the colour shade may indicate the adjusted *p* value, with darker colours indicating higher significance. (B) KEGG pathway enrichment analysis bubble map showing significantly enriched signalling pathways, such as Ras signalling pathway, MAPK signalling pathway and TNF signalling pathway. The size of the bubbles may indicate the number of enriched genes, and the colour shade indicates the adjusted *p* value. (C) GSEA plot showing the enrichment of gene sets in a sorted list, with the peak of the curve indicating where the gene set is most significantly enriched in the list. The graph lists several significantly enriched pathways, such as epithelial–mesenchymal transition, oxidative phosphorylation, MYC targets and protein secretion. (D, E) Results of somatic mutation analysis in high‐risk and low‐risk patients, showing the differences in somatic mutations in the high‐risk and low‐risk patient groups.

Differences in relevant biological pathways in patients from two different risk scoring groups were analysed using GO and KEGG enrichment methods. In the GO enrichment analysis, biological processes were predominantly associated with categories such as small GTPase‐mediated signal transduction, establishment of organelle localisation, cellular component disassembly, proteasome‐mediated ubiquitin‐dependent protein catabolic process and positive regulation of protein localisation. Cellular component enrichment revealed significant associations with structures including cell‐substrate junction, focal adhesion, mitochondrial matrix and nuclear speck. Molecular function categories showed enrichments for activities like protein serine/threonine kinase activity, GTPase binding, cadherin binding and GTPase regulator activity. These findings collectively highlight key regulatory mechanisms and subcellular compartments involved in the studied phenomena, providing valuable insights into the underlying biological processes (Figure 3A). The KEGG pathway enrichment analysis reveals several significantly enriched signalling pathways, including those associated with neurodegenerative diseases, the MAPK signalling pathway, the TNF signalling pathway and more (Figure 3B). Gene Set Enrichment Analysis (GSEA) identified several pathways with significant differences, notably epithelial–mesenchymal transition, oxidative phosphorylation, MYC targets and protein secretion (Figure 3C). Furthermore, the high‐risk group exhibited a higher frequency and greater diversity of somatic mutations compared with the low‐risk group (Figure 3D,E).

### 3.4 Risk Modelling Applications

**FIGURE 4:**
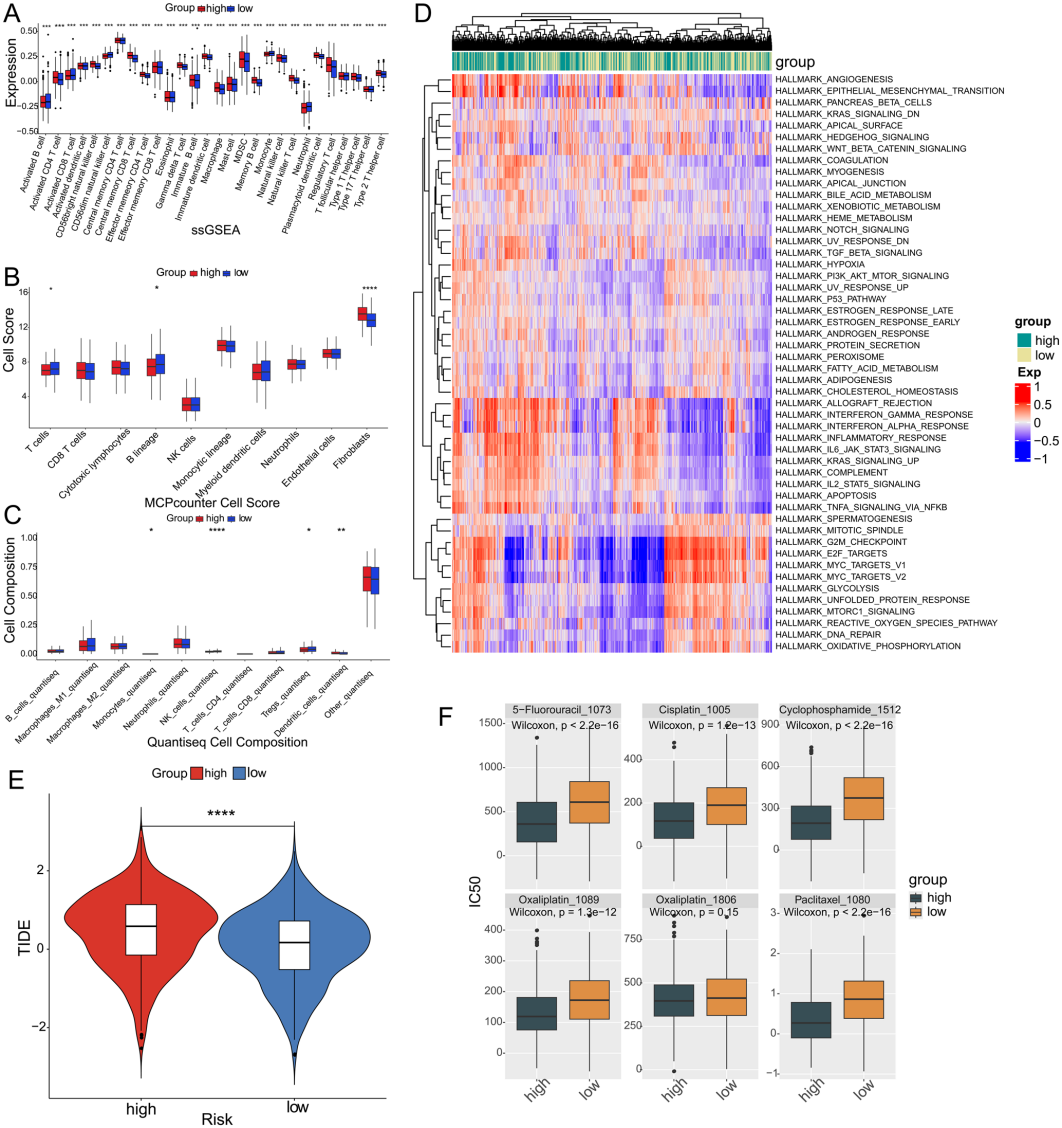
For assessing differences in immune cell infiltration, tumour immune microenvironment, biological pathway differences, and chemotherapeutic drug sensitivity in high‐risk and low‐risk patient populations. (A) ssGSEA analysis of differences in immune infiltration between high‐risk and low‐risk patient groups. (B) MCPcounter analyses differences in immune infiltration as well as stromal cells between high‐risk and low‐risk patient groups. (C) Comparison of the difference in immune infiltration between the high‐risk and low‐risk patient groups using the quanTIseq algorithm. (D) GVSA analysis of pathway enrichment differences between high‐risk and low‐risk patient groups. (E) The violin plot demonstrates the distribution of TIDE scores in the high‐risk and low‐risk patient groups, with differences in immunotherapy responsiveness between patients in the high‐risk and low‐risk groups. (F) OncoPredict analysis of the difference in sensitivity to chemotherapeutic agents between the two groups.

Following the division of patients into high‐risk and low‐risk groups, the results of ssGSEA analysis showed a significant difference in the degree of immune cell infiltration between the two groups (Figure 4A). MCPcounter results showed that patients in the high‐risk group had a higher degree of fibroblast infiltration than patients in the low‐risk group, and a lower degree of T‐cell and B‐lineage cell infiltration than patients in the low‐risk group (Figure 4B). The results using the quanTIseq algorithm showed that the degree of dendritic cell infiltration was significantly higher in high‐risk patients than in low‐risk patients, and the degree of NK cells, Tregs and monocytes infiltration was significantly lower in high‐risk patients than in low‐risk patients (Figure 4C). GSVA enrichment analysis was performed on the two groups, and significant differences in the pathways enriched in each group were found, such as angiogenesis, epithelial–mesenchymal transition and interferon γ response signalling pathway (Figure 4D). Analysis of the patients' TIDE scores revealed that the TIDE scores of high‐risk patients were significantly higher than those of patients in the low‐risk group (Figure 4E), suggesting that patients in the high‐risk group had a higher immune escape potential and a lower likelihood of benefiting from anti‐PD‐1/CTLA4. The results of drug sensitivity analysis showed that the sensitivity to chemotherapeutic agents such as 5‐fluorouracil, cisplatin, cyclophosphamide, oxaliplatin and paclitaxel was lower in the high‐risk group than in the low‐risk group (Figure 4F). In the validation set, there were significant differences in survival prognosis, immune cell infiltration and treatment responsiveness between patients in the high‐risk and low‐risk groups (Figure S1). The resulting graph shows the proportion of high‐risk and low‐risk groups by age, sex and TNM stage (Figure S2). Excitingly, we found that the prognostic prediction model was also effective in predicting the prognosis of patients with tumours other than NSCLC. Specifically, the model showed a significant difference in prognosis between high‐ and low‐risk groups for gastric, liver, ovarian and urothelial cancers. In addition, the model showed similar trends in predicting the prognosis for colorectal, pancreatic and breast cancer patients (Figure S3).

### 3.5 Nomogram Construction and Evaluation

**FIGURE 5:**
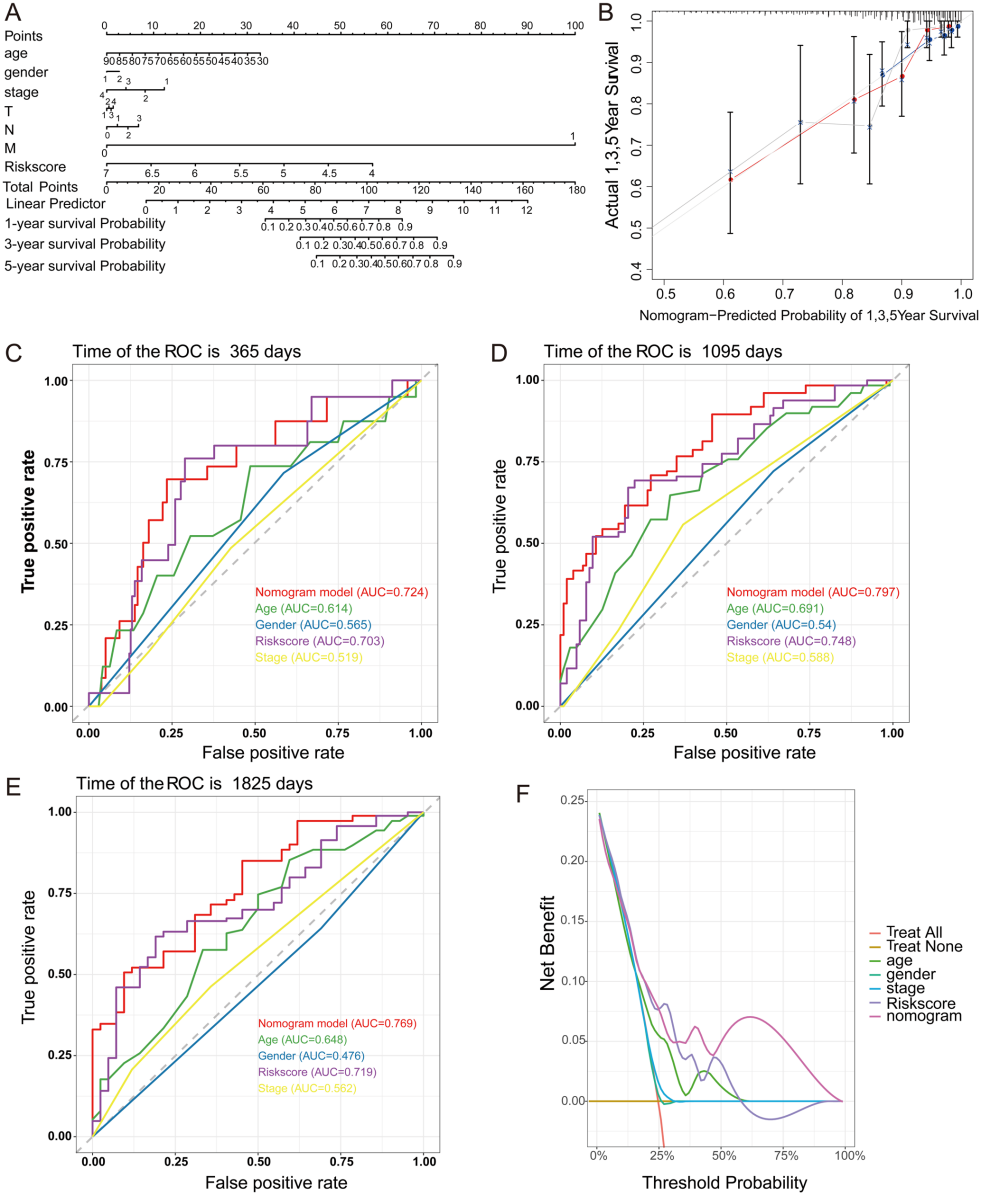
Nomogram development and model evaluation. (A) The nomogram was developed on the basis of risk scores combined with clinical factors. Gender, 1 is male, 2 is female. Stage indicates the grading of the cancer, grade I–IV. T‐stage indicates the condition of the primary tumour, T1–T4 indicate that the larger the tumour is or the more aggressive the tumour is. N‐stage indicates lymph node involvement, N0 indicates no involvement, N3 indicates extensive spread. M stage is an indicator of distant metastasis, M0 indicates no, and M1 indicates yes. (B) The calibration curves showed that the constructed nomograms were good predictors of 1‐, 3‐ and 5‐year patient survival. (C–E) The ROC curves demonstrated that the nomogram achieved higher predictive accuracy than the risk score and other clinical information, with AUCs of 0.724, 0.797 and 0.769 at 1‐, 3‐ and 5‐year follow‐ups. (F) The DCA curves showed that the nomograms had the highest value for practical clinical decision‐making compared with risk scores and other clinical information.

The Nomogram was further developed by integrating the patient's clinical information and risk scores, converting them into a friendly format that better quantifies the patient's prognostic risk (Figure 5A). The Calibration Plot demonstrates the concordance between the predicted survival probabilities and the actual observed outcomes, signifying accurate predictions at 1, 3 and 5 years (Figure 5B). Evidently, the nomogram model exhibited high predictive accuracy across all time points, with its performance remaining steady over time. The area under the curves for 1, 3 and 5 years was 0.759, 0.734 and 0.784 respectively. In contrast, when considered as single factors, gender and age exhibited lower predictive accuracies, while the risk score demonstrated better predictive capabilities. These findings imply that risk score models incorporating multiple factors might be more effective in forecasting patient survival (Figure 5C–E). DCA presents the net benefits of different models at various threshold probabilities. A higher curve indicates a greater decision‐making benefit for the model at that specific threshold. The nomogram offers more substantial clinical decision benefits compared to other factors (Figure 5F).

### 3.6 Risk Gene Expression and Pattern of Mutation in NSCLC

**FIGURE 6:**
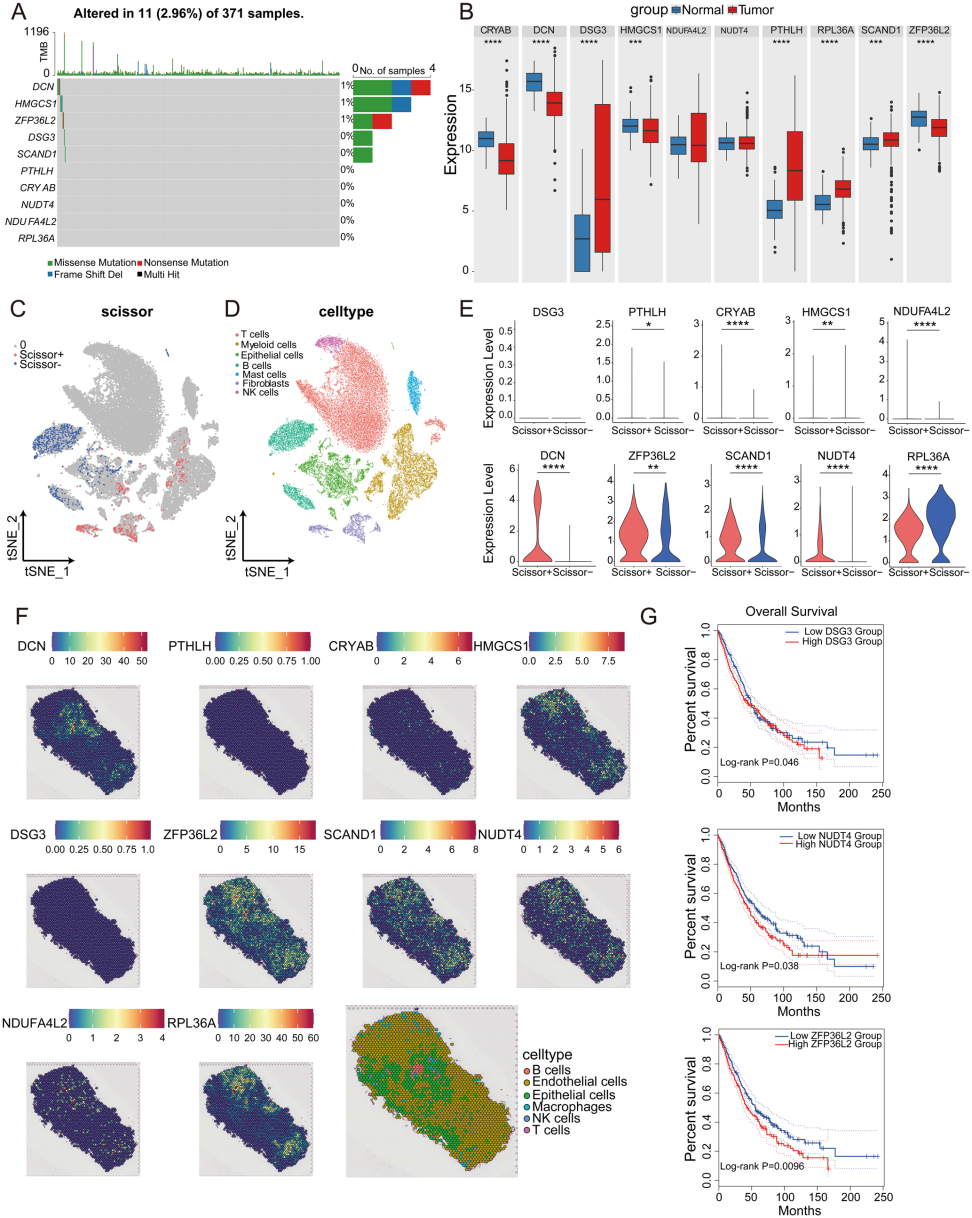
Expression and mutation patterns of risk genes at the bulk transcriptome, single‐cell and spatial transcriptome in NSCLC. (A) The results show the mutations in the 10 risk genes in the 371 samples. Colors indicate different mutation types or statuses. (B) Differential gene expression histogram comparing the expression levels of specific genes in normal and tumour tissues. Red and blue bars represent gene expression in different tissue types, respectively. (C) Single‐cell data were analyzed using the Scissor algorithm to identify subpopulations of cells associated with prognostic phenotypes, selecting cells highly correlated with phenotypes and visualizing them in reduced dimensions by the tSNE technique, thus demonstrating the distribution of these cells in low‐dimensional space. (D) The clustering of different cell types is shown by dimensionality reduction of single cell data using the tSNE algorithm. Different colors represent different cell populations, providing a view of the distribution of cell types in low‐dimensional space. (E) Results show differences in the expression of 10 risk genes in different Scissor scoring groups. Violin plots combining box and density plots show trends in the distribution and concentration of gene expression. (F) Results show the spatial expression patterns of the 10 risk genes in the spatial transcriptome data. Each subplot represents the expression of a gene in its spatial distribution, and different colors indicate the expression level as well as different cell types. This analysis helps to reveal the distribution characteristics of genes in tissues and their spatial relationship with the tumour microenvironment. (G) Survival curves showing the probability of survival over time in patients with risk genes DSG 3, NUDT 4, and ZFP 36 L2 in different expression groups.

We analysed the mutation patterns of 10 NSCLC risk genes and found mutations in DCN, HMGCS1, ZFP36L2, DSG3 and SCAND1, where the mutation patterns of DSG3 and SCAND1 were missense mutations, the mutation patterns of DCN were missense, nonsense and shifted‐code, the mutation patterns of HMGCS1 were missense and shifted‐code, and the mutation patterns of ZFP36L2 were nonsense mutation and missense mutation (Figure 6A). Analysis of TCGA data associated with NSCLC showed upregulation of DSG3, PTHLH, RPL36A and SCAND1 expression and downregulation of CRYAB, DCN, HMGCS1, NDUFA4L2, NUDT4 and ZFP36L2 expression, as compared with normal tissues (Figure 6B). To further validate the role of several risk genes in NSCLC, we used the Scissor algorithm, which combines single‐cell data, bulk RNA‐seq data and phenotypic information, to identify subpopulations of cells that are highly correlated with the disease prognosis, which are then visualised in low‐dimensional space by the tSNE technique to show their distribution. In addition, the tSNE algorithm was used for the downscaling of NSCLC single‐cell data to show clustering of different cell types, thus clearly demonstrating the distribution of cell types in low‐dimensional space. We also analysed the correlation between risk gene expression and Scissor score (Figure 6C–E). We also characterized the proportion, distribution, and expression of relevant marker genes for each cell type in the NSCLC single‐cell data (Figure S4). Immunohistochemical images from the Human Protein Atlas (HPA) database supported our analysis of risk gene expression in NSCLC (Figures S5 and S6). Spatial transcriptome data analysis confirmed that the spatial expression profiles of the risk genes aligned with our findings, except for DCN, which was not detected in the single‐cell sequencing data. However, this analysis indicated that DCN expression was predominantly high in epithelial cells, with minor expression in macrophages and endothelial cells (Figure 6F). Prognostic analysis showed that DSG3, ZFP36L2 and NUDT4 could be used as prognostic markers for NSCLC (Figure 6G). Drug prediction analyses revealed that DSG3, PTHLH, ZFP36L2, DCN and NDUFA4L2 have potential as therapeutic target genes (Table S6).

### 3.7 Expression of RPL36A in Lung Cancer

**FIGURE 7:**
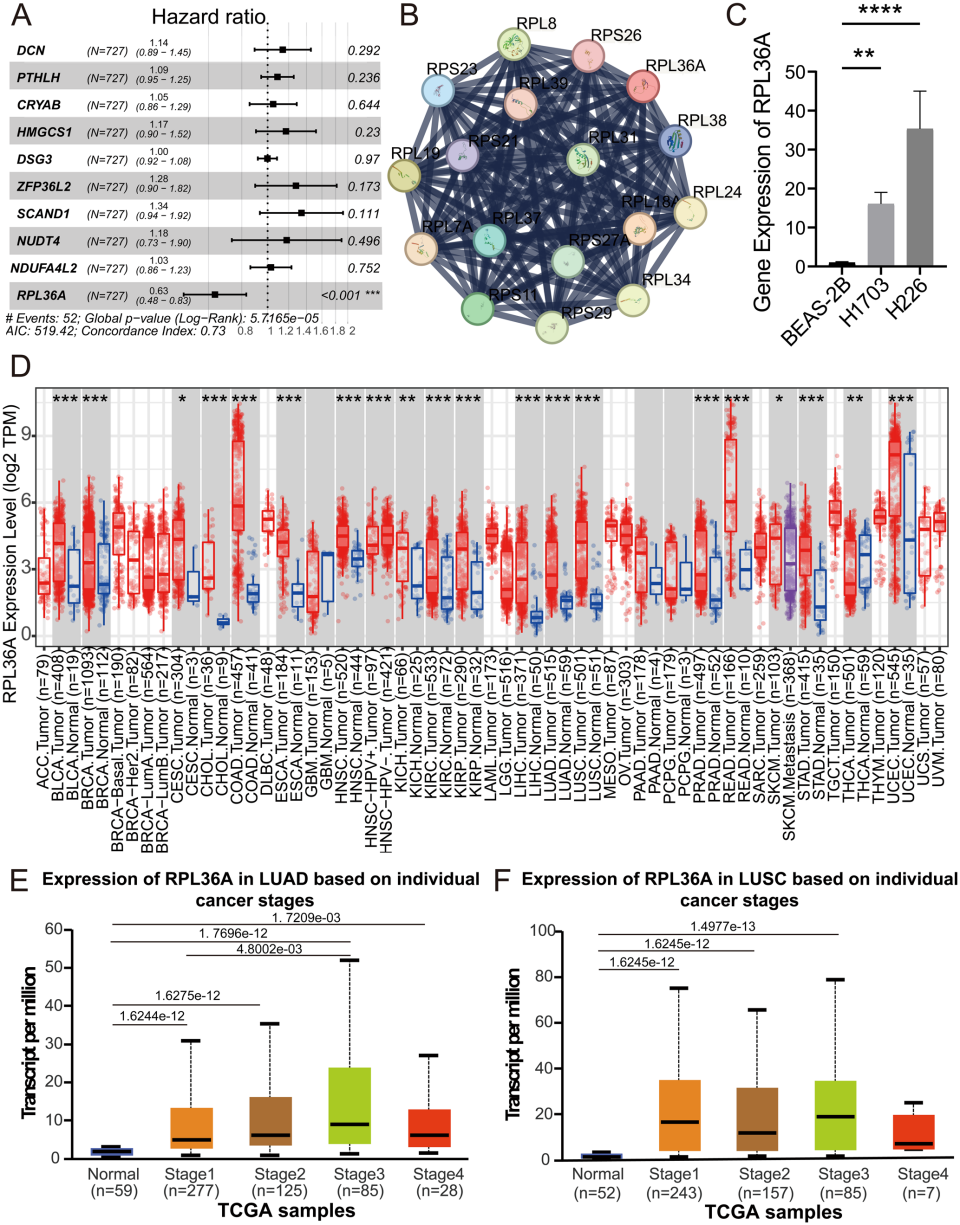
Expression of RPL36A in NSCLC. (A) Multivariable Cox regression analysis of the prognostic role of several risk genes in non‐small cell lung cancer. (B) Interaction network graph showing the gene network associated with the RPL36A gene. Nodes represent genes and edges represent interactions or associations between genes. Related interacting proteins include RPS27, RPL37A, MRL33 and HSPB2. (C) The results of qPCR of RPL36A gene, the bar graph demonstrated the expression level of RPL36A gene in different cell lines (BEAS‐2B, H1703, H226), the outcome of which was consistent with the results of previous analysis. (D) Pan‐cancer analysis showing the expression pattern of RPL36A in different tumour types, with different colors representing different sample types. (E, F) Box line plots demonstrate the expression levels of RPL36A gene in different stages of lung adenocarcinoma and squamous lung cancer. The role of RPL36A in disease progression was assessed by comparing the expression differences in different stages.

To further explore the role of several risk genes in NSCLC, we conducted multivariable Cox regression analysis on selected genes. The results indicated that RPL36A could serve as an independent prognostic factor (Figure 7A). We constructed an RPL36A‐related protein interaction network, identifying relevant interacting proteins such as RPS27, RPL37A, MRPL33 and HSPB2 (Figure 7B). To validate these findings, qPCR experiments were performed to measure RPL36A expression in NSCLC cell lines. The results corroborated our analysis (Figure 7C). Pan‐cancer analysis of RPL36A expression revealed significant differences across various tumour types, including hepatocellular carcinoma, breast cancer, colon cancer and NSCLC, compared to normal tissues (Figure 7D). Additionally, RPL36A expression showed significant variation among patients at different cancer stages (Figure 7E,F).

## 4 Discussion

Progress has been made in the treatment of NSCLC in recent years; however, the 5‐year survival rate for NSCLC patients is still approximately 15% [25]. Copper plays a significant role in modulating the immune response, affecting how the body reacts to various conditions [26]. High copper toxic activity reduces the immune response and still improves the prognosis of a wide range of tumours, but high immunity does not mean a good prognosis, which is related to the characteristics of the tumour [27]. Mitochondrial depolarisation is a complex process that is usually hampered in the heterogeneous and dynamic tumour microenvironment [24, 25]. However, the onset of mitochondrial depolarisation and consequently cell death plays an intricate and elusive role in cancer eradication and immune evasion of tumour cells [28]. Nevertheless, related studies have not yet examined the relationship between cuproptosis, mitochondrial depolarisation, and non‐small cell lung cancer.

Mitochondria serve as the powerhouses of the cell, responsible for generating the majority of the ATP required by cells. In cancer, abnormalities in mitochondrial function, including depolarisation, can lead to the reprogramming of cellular energy metabolism pathways to meet the demands of rapid tumour proliferation [29]. In NSCLC cells, mitochondrial depolarisation may promote this metabolic shift, supporting tumour growth and development [30]. Mitochondrial depolarisation can also increase reactive oxygen species (ROS) levels; high ROS levels can cause DNA damage, protein modifications and other effects that further promote tumour initiation and progression [31]. Although research on mitochondrial depolarisation in lung cancer is still limited, our analysis has revealed that genes related to mitochondrial depolarisation play significant roles in disease progression and prognosis prediction in NSCLC patients. The results of the single‐cell sequencing data analysis also showed that several mitochondrial depolarisation‐associated risk genes were significantly upregulated in the subset of cells that scored high. This finding underscores the crucial role of mitochondrial depolarisation in the development and progression of lung cancer, highlighting its potential as a target for therapeutic intervention.

This study examined the correlation between cuproptosis‐related mitochondrial depolarisation genes in lung cancer using univariate Cox regression analysis and machine learning algorithms, identifying 10 risk genes valuable for predicting the prognosis of NSCLC patients and guiding subsequent treatment and follow‐up programmes. It is well known that many NSCLC patients are already in advanced stages when first diagnosed, leading to a grim prognosis with much shorter survival once drug resistance develops. In view of this, precise and personalised treatment strategies are crucial for improving efficacy. With the help of Kaplan–Meier curve analysis, we clearly observed that the survival rate of patients in the low‐risk group was significantly higher than that of the high‐risk group. This not only aids in the early detection of high‐risk patients but also provides an important basis for doctors to make stratified treatment decisions based on risk scores. For high‐risk patients, doctors can consider more aggressive treatment regimens with stricter follow‐up monitoring, while low‐risk patients can follow routine treatment and follow‐up processes. Such risk‐based treatment and monitoring strategies are expected to improve overall outcomes in NSCLC. Immunotherapy has achieved significant efficacy in treating NSCLC, but patient responses vary widely. Currently, PD‐L1 expression levels are used to predict immunotherapy response; yet the prediction accuracy remains below 20%, making it imperative to find more accurate predictive markers [32, 33]. Notably, the risk scores constructed in this study can help physicians predict the response to immunotherapy more accurately. For example, the TIDE score within our model indicates that high‐risk patients have a higher tendency towards immune escape and a higher probability of being insensitive to immunotherapy. This result is expected to greatly improve the accuracy of immunotherapy predictions, avoid the overuse of immune drugs and optimise the allocation of medical resources.

Our model also predicted the tumour microenvironment, treatment benefits and chemotherapy sensitivity. GSVA enrichment analysis revealed significant differences in pathway enrichment between the two groups, particularly in angiogenesis, epithelial–mesenchymal transition (EMT) and the interferon‐γ (IFN‐γ) response signalling pathway. Angiogenesis plays a crucial role in tumour growth and metastasis [34]. EMT enhances tumour invasion and contributes to drug and radioresistance, making it important for improving patient prognosis [35]. IFN‐γ has complex regulatory effects on the tumour microenvironment during lung cancer development [36]. These pathways were upregulated in the high‐risk group, indicating their potential as therapeutic targets for NSCLC. The upregulation of these pathways in the high‐risk group validates the model's accuracy in differentiating prognosis and therapeutic outcomes between high‐and low‐risk patients.

By analysing immune cell infiltration in different patient groups, we found that dendritic cell (DC) infiltration was significantly higher in high‐risk patients compared to low‐risk patients. Conversely, the infiltration levels of natural killer (NK) cells, regulatory T cells (Tregs) and monocytes were significantly lower in high‐risk patients. Combining the results from the TIDE score, GSEA and immune infiltration analysis, we observed that high‐risk patients exhibited higher interferon‐γ pathway activity, lower Treg infiltration, higher DC infiltration and higher TIDE scores. We speculate that the increased interferon‐γ activity in high‐risk patients may cause Treg polarisation, reducing their infiltration. This subsequent reduction in Tregs could lead to increased DC infiltration, potentially diminishing the therapeutic benefit of anti‐PD‐1/CTLA4 treatments. Hui et al. identified several key events associated with positive clinical outcomes in NSCLC treatment, including a decrease in regulatory T cells and an increase in dendritic cells [37]. This finding aligns well with our observations, suggesting that despite being classified as high risk, these patients may still benefit from neoadjuvant treatment with immune checkpoint inhibitors combined with chemotherapy. Interestingly, the paradox of immune infiltration observed in our primary set was not replicated in the validation set, indicating that this phenomenon may relate to the individual immune status of the patient as well as the disease state.

In the course of our study, we found that RPL36A, which encodes a ribosomal protein component of the 60S subunit, can serve as an independent prognostic factor in NSCLC [38]. Expression analysis revealed significant differences and upregulation of RPL36A in various tumours, including breast cancer, renal clear cell carcinoma, hepatocellular carcinoma, pancreatic cancer and lung cancer, compared to normal tissues. Conversely, it was downregulated in thyroid cancer. The expression levels of RPL36A gene in different stages of lung adenocarcinoma and lung squamous carcinoma also differed significantly,and this difference reflects the different roles of RPL36A gene in tumour progression,which may be related to the biological behaviors of tumour growth,invasion and metastasis. Protein interaction network analysis identified several proteins interacting with RPL36A, including RPL36AL, RPL37, RPS27, HSPB2 and EEF1B2. Related studies suggest that RPL36 may be involved in the early stages of hepatocellular carcinoma, acting as an independent and potentially prognostic indicator for resection of hepatocellular carcinoma, and playing a prognostic role in renal clear cell carcinoma [39, 40]. These findings highlight the multifaceted role of RPL36A across different types of cancer and underscore its potential as a biomarker and therapeutic target.

In the analysis of risk genes using single‐cell data, we observed that, apart from DSG3, all selected risk genes exhibited significant correlations with Scissor scores. Notably, our findings regarding RPL36A's role in patient prognosis were particularly interesting. Incorporating RPL36A into the prognostic model indicated its association with a favourable outcome, as patients with higher RPL36A expression levels had lower risk scores. Single‐cell data analysis further revealed that RPL36A expression was markedly decreased in Scissor+ cells compared to Scissor‐ cells, suggesting a positive correlation between RPL36A expression and better patient outcomes. Both univariate and multivariate Cox regression analyses confirmed that the hazard ratio (HR) of RPL36A was below 1, reinforcing its link to a good prognosis. However, TCGA data analysis showed increased RPL36A expression in tumour tissues, a finding supported by qPCR validation. This discrepancy suggests a more complex role for RPL36A, indicating that elevated expression in tumours may be involved in tumour‐suppressive mechanisms rather than directly correlating with poor prognosis. It is likely that RPL36A influences patient outcomes beneficially by modulating other genes or pathways that inhibit tumour growth. Thus, despite the initial inconsistency, we conclude that higher RPL36A expression is strongly associated with improved patient prognosis through intricate cellular interactions. The complexity of the role of RPL36A highlights the need for further investigation into how this gene interacts within the tumour microenvironment and contributes to therapeutic responses.

Regarding drug predictions for several risk genes, we have identified potential interactions between DSG3, PTHLH, ZFP36L2, DCN and NDUFA4L2 and various drugs. For instance, PTHLH interacts with vincristine (previously referred to as vincaleukoblastine), barium sulfate anhydrous and dimethyl sulfoxide; ZFP36L2 is associated with saccharin and acesulfame potassium; DCN interacts with rapamycin and ascorbic acid; and NDUFA4L2 shows interaction with metformin hydrochloride. These interactions suggest that these genes hold promise as novel therapeutic targets for NSCLC. However, it is important to note that the data originate from public databases and are largely supported by existing experimental evidence. Future research should focus on conducting prospective studies and using institutional data to validate the interactions and therapeutic potential of these genes, investigating the underlying mechanisms by which these genes interact with the respective drugs and exploring how variations in these genes can inform personalised treatment strategies to enhance therapy efficacy for individual patients. By advancing our understanding of these gene–drug interactions, we can pave the way for more effective and targeted treatments for NSCLC, improving patient outcomes.

In conclusion, we have developed a highly accurate prognostic prediction model using cuproptosis‐related mitochondrial depolarisation genes to predict prognosis and response to immunotherapy and chemotherapy in lung cancer patients. Early identification of high‐risk individuals, suitable candidates for immunotherapy, as well as therapies such as chemotherapy, will help clinicians implement appropriate treatments with follow‐up and monitoring intensity in accordance with risk stratification. This approach is expected to greatly improve the outcome of NSCLC. Our study demonstrates that the model has significant predictive value for the prognosis of NSCLC and has the potential to predict the prognosis of other cancers such as ovarian, liver, urothelial and gastric cancers. Additionally, we identified lung cancer tumour markers and target genes associated with mitochondrial depolarisation and cuproptosis, examining their expression patterns at multiple transcriptome levels. These findings underscore the broad applicability and potential impact of our model on personalised medicine and cancer treatment strategies.

## Author Contributions

**Guoqing Lyu:** conceptualization (equal), formal analysis (supporting), methodology (equal), project administration (lead), supervision (equal), writing – review and editing (equal). **Lihua Dai:** data curation (lead), formal analysis (equal), methodology (lead), resources (equal), validation (equal), visualization (equal), writing – original draft (equal), writing – review and editing (lead). **Xin Deng:** data curation (equal), formal analysis (equal), methodology (lead), resources (equal), validation (equal), visualization (lead), writing – original draft (lead), writing – review and editing (equal). **Xiankai Liu:** data curation (equal), formal analysis (equal), methodology (equal), supervision (equal), writing – review and editing (equal). **Yan Guo:** data curation (equal), validation (equal), visualization (equal). **Yuan Zhang:** data curation (equal), validation (equal). **Xiufeng Wang:** formal analysis (equal), investigation (equal). **Yan Huang:** formal analysis (equal), supervision (equal). **Sun Wu:** data curation (supporting), formal analysis (supporting), project administration (supporting), supervision (supporting), visualization (supporting). **Jin‐Cheng Guo:** data curation (supporting), project administration (supporting), supervision (supporting), validation (supporting), visualization (supporting), writing – review and editing (supporting). **Yanting Liu:** conceptualization (supporting), formal analysis (supporting), project administration (supporting), software (supporting), writing – review and editing (supporting).

## Ethics Statement

The data used in this study were obtained from publicly available data in TCGA, ICGC, BioStudies and GEO databases and did not involve ethical approval or consent to participate.

## Consent

All authors have consented to the publication of the manuscript.

## Conflicts of Interest

The authors declare no conflicts of interest.

## Supporting information


Figures S1‐S6.

Tables S1‐S6.

## Data Availability

Data for this study were obtained from the TCGA, GEO, ICGC and BioStudies databases. Detailed information on dataset accessions and specific data used in this study can be found in the Materials and Methods section. For any additional data not deposited in these repositories, please contact the corresponding author. The list of related genes appearing in the article are shown in Table S1. Primer sequences are detailed in Table S2. The results of the correlation analysis between cuproptosis and mitochondrial depolarising genes and DEGs are shown in Table S3. The results of the univariate cox regression analysis to screen for CRMDGs are shown in Table S4. The results of the Lasso regression analysis to screen for risk genes are shown in Table S5. The results of the analysis of the predictive outcome of the risk genes, the DGIdb database drug, are shown in Table S6.
