# Supplementary material for: Integrative Analysis of Cuproptosis‐Related Mitochondrial Depolarisation Genes for Prognostic Prediction in Non‐Small Cell Lung Cancer
Source: J Cell Mol Med. 2025 Feb 26;29(4):e70438. doi: 10.1111/jcmm.70438 (PMC11862892; doi:10.1111/jcmm.70438)
Supplement: Supplementary file 1 — Figures S1‐S6. [file JCMM-29-e70438-s001.docx]

­­­­
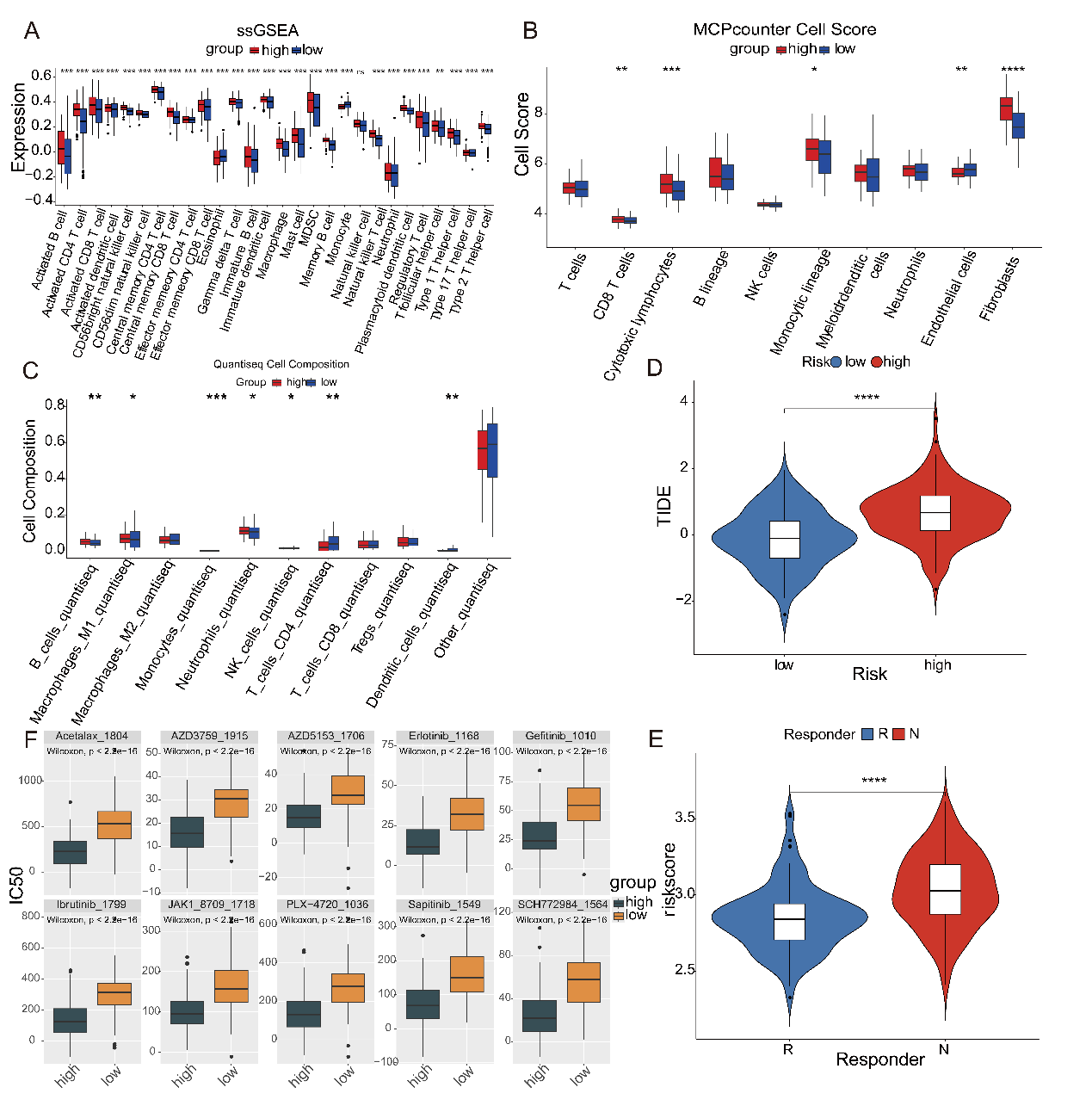


Supplementary Figure 1. (A) The ssGSEA analysis of the difference in immune infiltration between the two groups of patients in the high- and low-risk groups. (B) MCPcounter analysis of the difference in immune infiltration between the two groups of patients in the high- and low-risk groups. (C) The quanTIseq algorithm calculates stromal, immune, and estimated scores as well as tumor purity in both risk groups. (D-E) TIDE analysis of immunotherapy responsiveness in two groups of patients in the high- and low-risk groups. (F) The oncoPredict software package predicts drug susceptibility in patients in high and low risk groups.


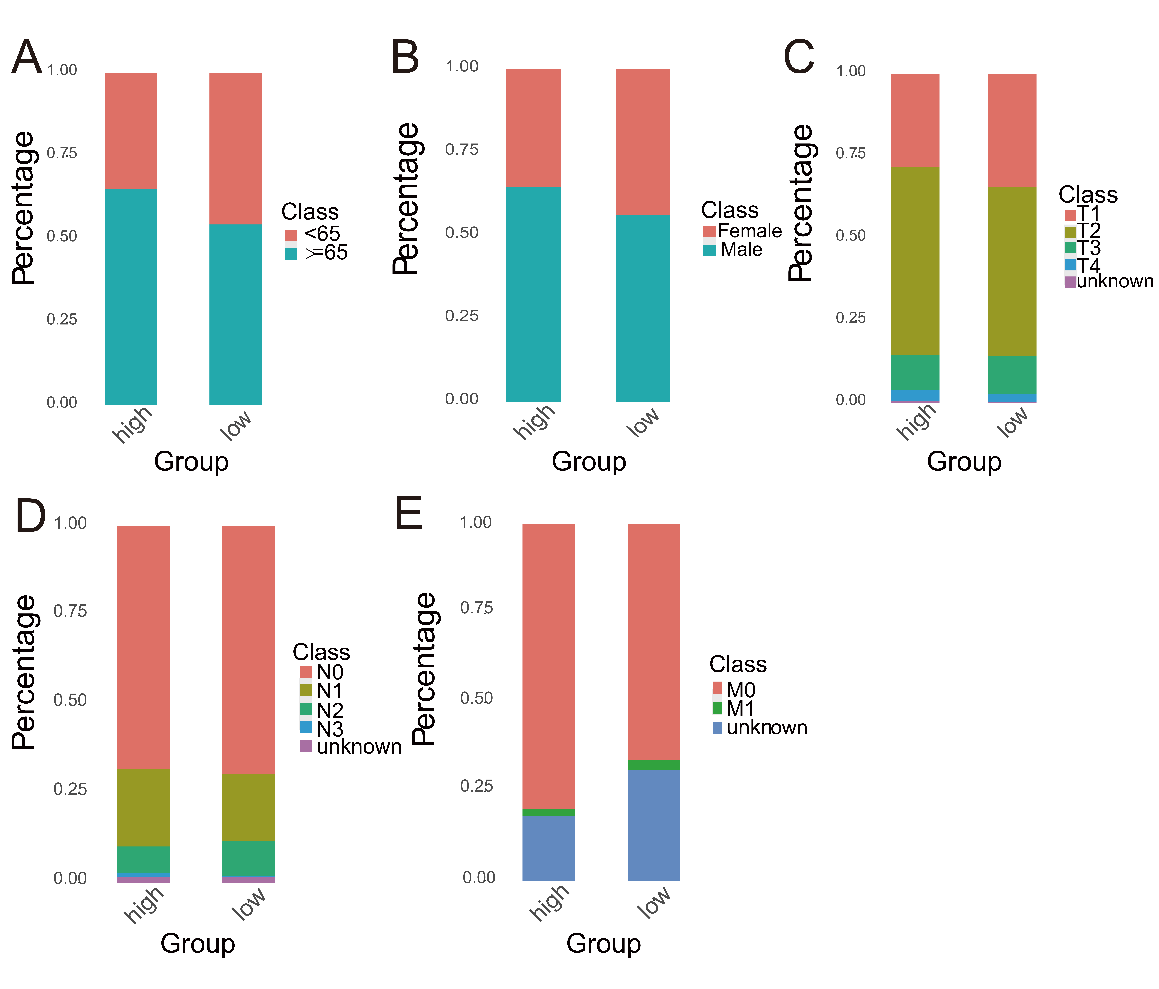


Supplementary Figure 2. Distributional outcomes in clinicopathologic characteristics based on risk modeling. (A-E) Distribution of clinicopathologic characteristics of patients in high and low risk groups, such as age, gender, and TNM stage. The age distribution by risk group was as follows: less than 65 years (35% vs. 46%) and 65 years or older (65% vs. 54%). The gender distribution in the risk groups was male (64% vs. 56%) and female (36% vs. 44%). The distribution of patients by T-stage was T1 (28% vs. 34%), T2 (57% vs. 59%), T3 (10% vs. 11%), and T4 (3% vs. 2%). The distribution by N-stage was N0 (68% vs. 70%), N1 ­­­(22% vs. 19%), N2 (7% vs. 10%), and N3 (1% vs. 0.2%). Finally, the distribution by M-stage was M0 (79% vs. 66%) and M1 (2% vs. 3%).


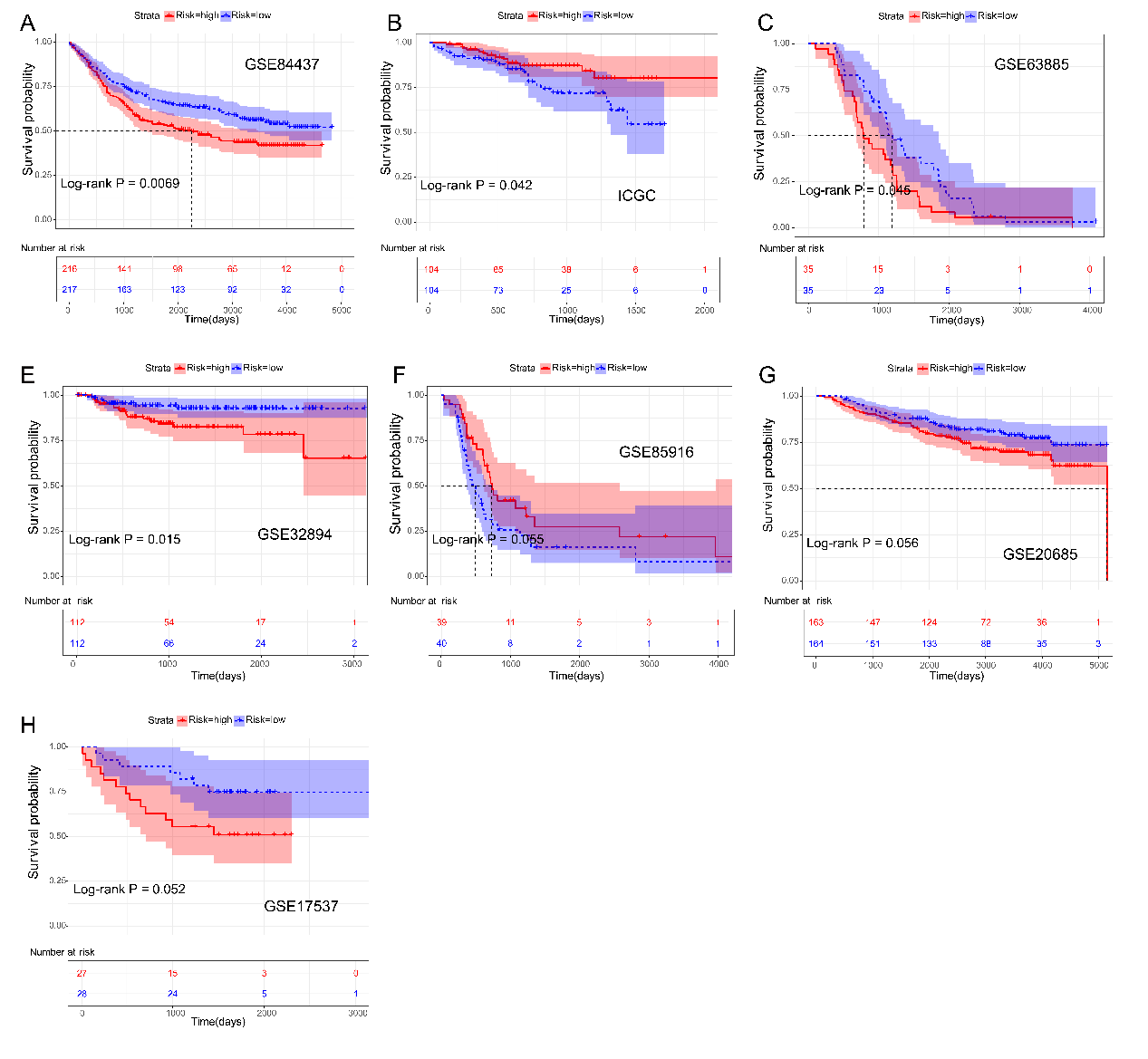


Supplementary Figure 3. (A-H) The prognostic prediction model predicts outcomes for different tumor types including gastric, liver, ovarian, urothelial, colorectal, pancreatic, and breast cancers.


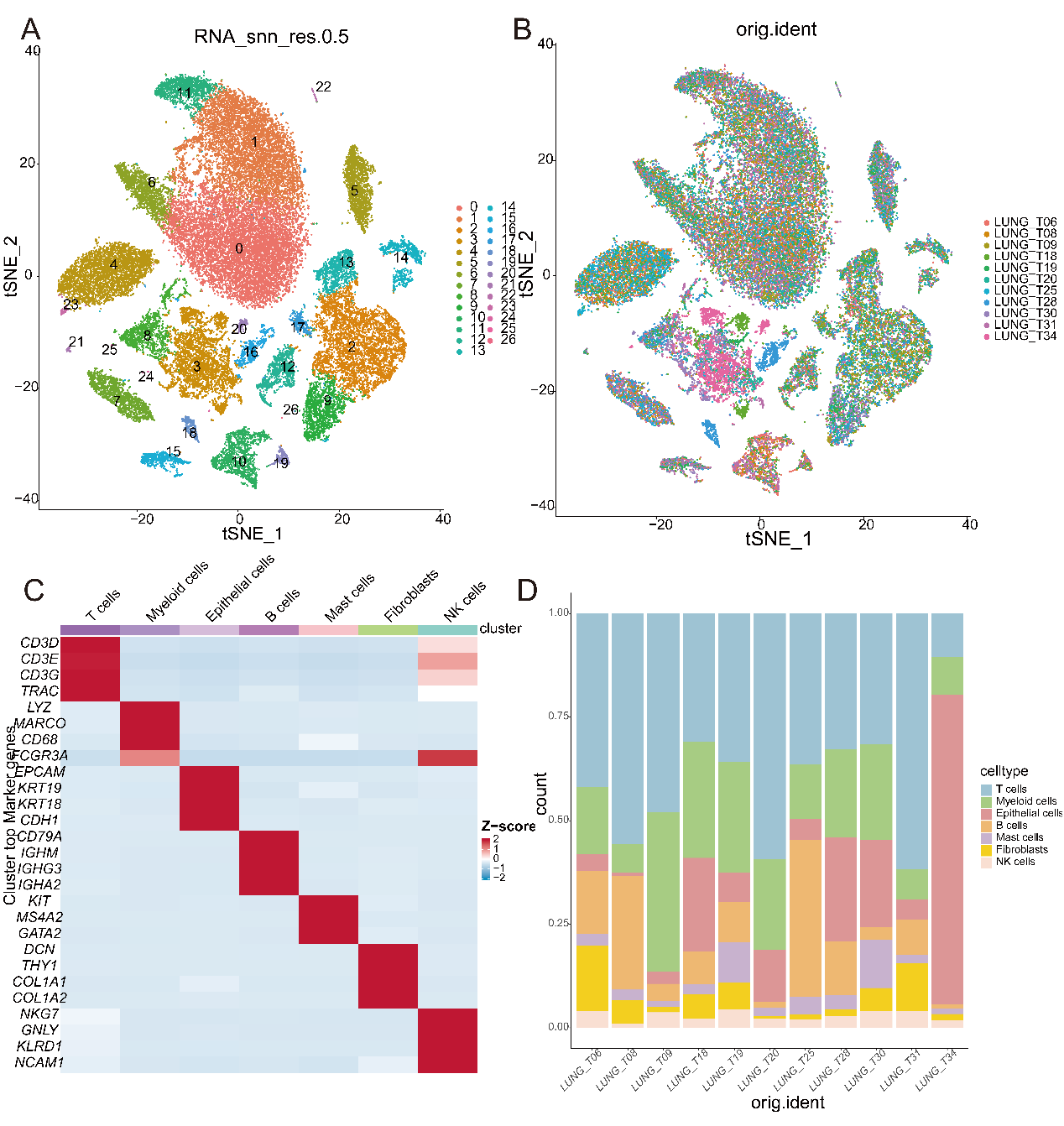


Supplementary Figure 4. The proportion, distribution and specific gene expression of each cell type in immune microenvironment of NSCLC single-cell data in validation set. (A-B) t-SNE cluster analysis shows the distribution of different cell clusters, and sample source identification shows the original sample to which each cell belongs. (C) Average expression levels of specific genes in different cell types are shown. (D) The relative proportions of various cell types in different samples are shown.


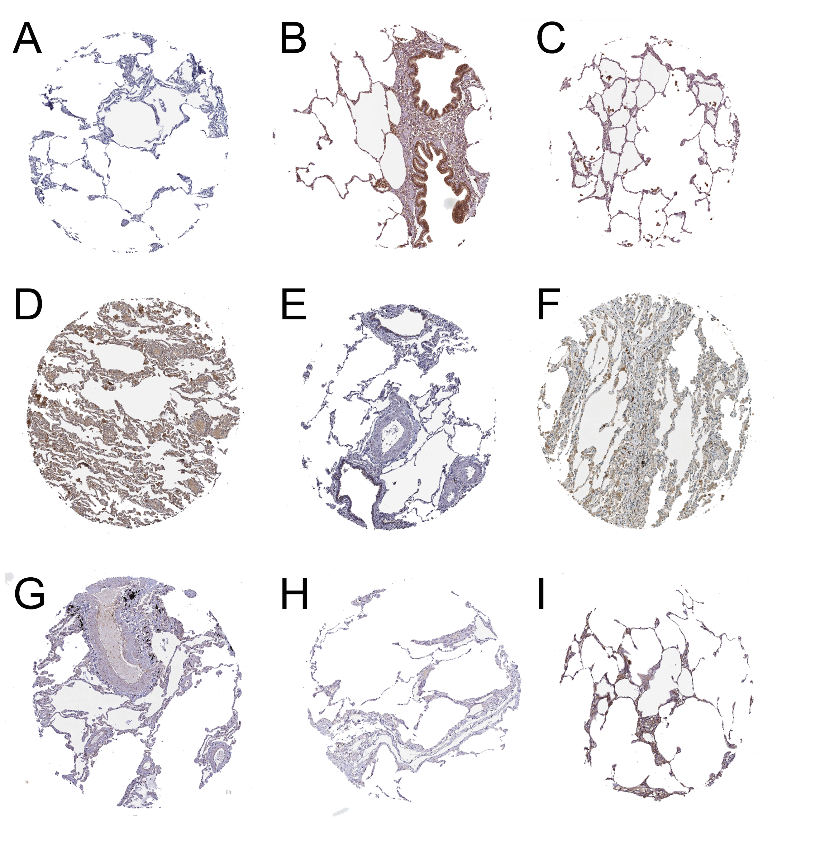


Supplementary Figure 5. Expression of CRYAB, DCN, DSG3, HMGCS1, NDUFA4L2, NUDT4, PTHLH, RPL36A and ZFP36L2 in normal lung tissues.


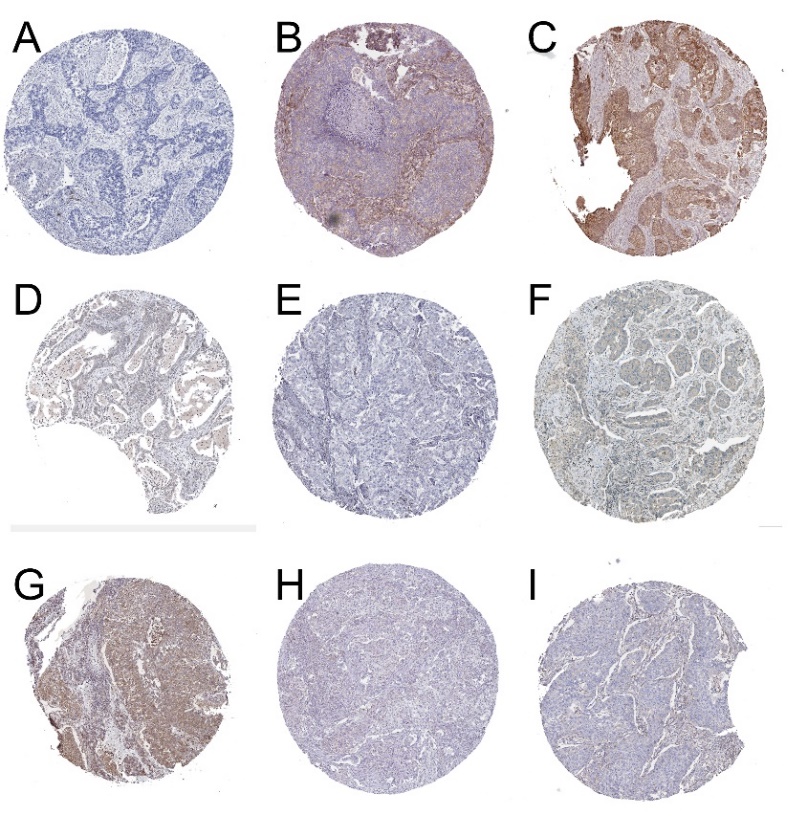


Supplementary Figure 6. Expression of CRYAB, DCN, DSG3, HMGCS1, NDUFA4L2, NUDT4, PTHLH, RPL36A and ZFP36L2 in lung cancer tissues.
