# Supplementary material for: Integrative Analysis of Cuproptosis‐Related Mitochondrial Depolarisation Genes for Prognostic Prediction in Non‐Small Cell Lung Cancer
Source: J Cell Mol Med. 2025 Feb 26;29(4):e70438. doi: 10.1111/jcmm.70438 (PMC11862892; doi:10.1111/jcmm.70438)
Supplement: Supplementary file 2 — Tables S1‐S6. [file JCMM-29-e70438-s002.docx]

Supplementary Table 1. The list of related genes appearing in the article. This table provides a comprehensive list of all relevant genes involved in this study, categorized into several key groups: cuproptosis-related genes, differentially expressed genes and mitochondrial depolarization-associated genes, genes identified through correlation analyses, genes selected following univariate Cox regression screening, and risk genes determined after Lasso regression analysis.

| Cuproptosis-related gene | DEGs and MDRGs | Correlation analysis | Univariate cox regression | Lasso |
| --- | --- | --- | --- | --- |
| ATP7B | ABCD1 | GLS | CD9 | DCN |
| ATP7A | ABL1 | CDKN2A | DCN | PTHLH |
| CDKN2A | ATP5IF1 | GCLC | RPL18 | CRYAB |
| DLD | BCL2 | CFLAR | GPC1 | HMGCS1 |
| DLAT | BOK | PDK4 | IDI1 | DSG3 |
| DBT | DCN | SLC25A5 | KLF6 | ZFP36L2 |
| DLST | FZD9 | POLR2J | ATP1B3 | SCAND1 |
| FDX1 | GCLC | CD9 | PTHLH | NUDT4 |
| GLS | GCLM | DCN | PSMD8 | NDUFA4L2 |
| GCSH | HSH2D | GCLM | SLC25A1 | RPL36A |
| LIAS | IFI6 | TMSB10 | EIF5 |  |
| LIPT1 | KDR | THOC3 | AHCY |  |
| LIPT2 | LRRK2 | RPL18 | PSMD7 |  |
| MTF1 | MLLT11 | GPC1 | FBL |  |
| NFE2L2 | MYOC | IDI1 | CRYAB |  |
| NLRP3 | P2RX7 | KLF6 | HSPA8 |  |
| PDHA1 | PARP1 | ATP1B3 | GAPDH |  |
| PDHB | PPP2R3C | NUCB2 | PERP |  |
| SLC31A1 | RACK1 | RPL31 | HMGCS1 |  |
|  | SRC | ENO1 | RNF7 |  |
|  | TSPO | SRI | PDCD10 |  |
|  | HDAC6 | PCM1 | MFN2 |  |
|  | MFN2 | HSP90AA1 | SLC2A1 |  |
|  | OPTN | CXCL2 | CSTA |  |
|  | PRKN | CTTN | SNRPD2 |  |
|  | VPS13C | DNAJA1 | KRT17 |  |
|  | CDC37 | HUWE1 | MRPS12 |  |
|  | HUWE1 | FTL | DSG3 |  |
|  | PINK1 | PTHLH | RPL7A |  |
|  | TOMM7 | PIR | DLAT |  |
|  | GBA | KIF9 | ZFP36L2 |  |
|  | HK2 | DYNLL1 | KLF10 |  |
|  | HTRA2 | RPL6 | AZIN1 |  |
|  | MUL1 | P2RX7 | UBE2L6 |  |
|  | IFI27 | FUS | NLRP3 |  |
|  | ISG15 | FXYD3 | DAPL1 |  |
|  | BST2 | MLF2 | EEF2 |  |
|  | IFI44L | ICAM1 | SCAND1 |  |
|  | RPS15 | LYZ | PGAM1 |  |
|  | RPL36 | MUL1 | NUDT4 |  |
|  | RPL19 | PPP2R3C | EIF3K |  |
|  | RPL35 | EZR | NPM1 |  |
|  | RPL18A | COMT | NDUFA4L2 |  |
|  | RPL13 | HDAC6 | POLR1D |  |
|  | RPL32 | ABL1 | RPL10A |  |
|  | CSTA | PSMD8 | PHB2 |  |
|  | RPL39 | GADD45B | UBA52 |  |
|  | RPL37 | MKNK2 | RPS28 |  |
|  | DDX5 | RANBP1 | RPL36A |  |
|  | RPL12 | SLC25A1 |  |  |
|  | GPX2 | XBP1 |  |  |
|  | RPS7 | TSPO |  |  |
|  | IGKC | RPL3 |  |  |
|  | GPNMB | EIF5 |  |  |
|  | RPL11 | PSME2 |  |  |
|  | IFITM3 | AHCY |  |  |
|  | RPS18 | ABCD1 |  |  |
|  | TXN | PLP2 |  |  |
|  | NACA | PGK1 |  |  |
|  | RPL10 | KLF5 |  |  |
|  | HSPA1A | MRPS31 |  |  |
|  | GAPDH | INTS6 |  |  |
|  | RPS23 | TSC22D1 |  |  |
|  | MT-ND3 | PSMD7 |  |  |
|  | RPS2 | EEF1D |  |  |
|  | RPL29 | SQLE |  |  |
|  | KLF6 | PDCD5 |  |  |
|  | COX6B1 | RPS16 |  |  |
|  | RPL31 | FBL |  |  |
|  | KRT6A | GPI |  |  |
|  | RPS16 | RPS19 |  |  |
|  | RPL26 | CDC37 |  |  |
|  | UBE2L6 | RPL18A |  |  |
|  | RPS27 | DNAJB6 |  |  |
|  | COX7B | PDAP1 |  |  |
|  | RPL8 | BUD31 |  |  |
|  | FTH1 | ZNHIT1 |  |  |
|  | RPL7A | TLE4 |  |  |
|  | HSP90AA1 | ZFAND5 |  |  |
|  | ALDOA | NUFIP2 |  |  |
|  | RPS28 | RPL19 |  |  |
|  | RPL35A | ALDH3A1 |  |  |
|  | DNAJC19 | DDX5 |  |  |
|  | TPRG1 | PRKAR1A |  |  |
|  | CSTB | CRYAB |  |  |
|  | GNB2L1 | HSPA8 |  |  |
|  | CDKN2A | PTGES3 |  |  |
|  | LINC01133 | GAPDH |  |  |
|  | NTS | TPI1 |  |  |
|  | XIST | NEDD9 |  |  |
|  | RPL38 | TPD52L1 |  |  |
|  | DDX3X | SRSF3 |  |  |
|  | RPL36A | SOD2 |  |  |
|  | CD55 | CD83 |  |  |
|  | RPS14 | RPS12 |  |  |
|  | S100A11 | C6orf62 |  |  |
|  | NGFRAP1 | PERP |  |  |
|  | GPC3 | ARFGEF3 |  |  |
|  | RPL18 | HMGCS1 |  |  |
|  | G6PD | ARRDC3 |  |  |
|  | RPL6 | CPEB4 |  |  |
|  | FXYD3 | FAM162A |  |  |
|  | IFI16 | RNF7 |  |  |
|  | UBA52 | PDCD10 |  |  |
|  | HLA-B | COL7A1 |  |  |
|  | PNCK | HES1 |  |  |
|  | COL17A1 | ABCC5 |  |  |
|  | ZNHIT1 | KLHL24 |  |  |
|  | MX1 | DGUOK |  |  |
|  | SLC25A5 | RPS15 |  |  |
|  | HLA-C | HTRA2 |  |  |
|  | RPS8 | SRSF7 |  |  |
|  | DUSP1 | SFPQ |  |  |
|  | RBP1 | MFN2 |  |  |
|  | ABCC5 | SLC2A1 |  |  |
|  | PSMB9 | PRDX1 |  |  |
|  | APRT | HMGN3 |  |  |
|  | SLC25A6 | TNFAIP3 |  |  |
|  | YDJC | SLIRP |  |  |
|  | CTTN | DUSP1 |  |  |
|  | SPTSSA | CCDC170 |  |  |
|  | PIR | HSPH1 |  |  |
|  | S100A2 | CSTA |  |  |
|  | UBE2L3 | ODF2L |  |  |
|  | LAMTOR4 | OPTN |  |  |
|  | RPS19 | TREM1 |  |  |
|  | GPC1 | LRRFIP1 |  |  |
|  | AKR1C2 | IRF1 |  |  |
|  | TPD52L1 | SNRPD2 |  |  |
|  | TAP1 | RBCK1 |  |  |
|  | PARL | SNRPB |  |  |
|  | DSG3 | EIF2S2 |  |  |
|  | TKT | COX6B1 |  |  |
|  | ATP5J2 | AKAP9 |  |  |
|  | RPS15A | KDR |  |  |
|  | RPL27 | DGCR6L |  |  |
|  | EIF3K | KRT17 |  |  |
|  | AKR1B10 | MRPS12 |  |  |
|  | CLK1 | VPS13C |  |  |
|  | NCOA7 | RPL36 |  |  |
|  | PGLS | PGLS |  |  |
|  | NFKBIZ | RSPH3 |  |  |
|  | RPS6 | ATP5IF1 |  |  |
|  | TTC14 | SLC6A8 |  |  |
|  | RPS4X | PNCK |  |  |
|  | RPS12 | SYNE1 |  |  |
|  | DGCR6L | COX4I1 |  |  |
|  | C22orf39 | COX7B |  |  |
|  | ATF3 | RPL27 |  |  |
|  | PSME2 | DNAJB1 |  |  |
|  | ZFP36L1 | POMP |  |  |
|  | FTL | TCEAL4 |  |  |
|  | MRPS12 | NTS |  |  |
|  | RHOB | SARAF |  |  |
|  | BRD2 | RPS15A |  |  |
|  | POLR2J | DSG3 |  |  |
|  | RPL15 | ANXA1 |  |  |
|  | MDK | PRRG4 |  |  |
|  | NTRK2 | CD164 |  |  |
|  | ALDH3A1 | GPNMB |  |  |
|  | SFN | NDUFB5 |  |  |
|  | YEATS2 | MRPL47 |  |  |
|  | ELF3 | TRA2B |  |  |
|  | COX4I1 | TXN |  |  |
|  | SLC6A8 | RPL35 |  |  |
|  | RPL22L1 | RPS6 |  |  |
|  | RNF7 | RPLP1 |  |  |
|  | PDCD5 | KIF21A |  |  |
|  | THOC3 | DNAJA4 |  |  |
|  | MRPL17 | ABHD2 |  |  |
|  | RPS27A | RPS2 |  |  |
|  | COL7A1 | TOB1 |  |  |
|  | JUND | IFITM3 |  |  |
|  | TMSB10 | PGD |  |  |
|  | C9orf16 | SH3BGRL3 |  |  |
|  | SCGB1A1 | RPL11 |  |  |
|  | HLA-A | RPS8 |  |  |
|  | PGD | PFDN2 |  |  |
|  | MIDN | MCL1 |  |  |
|  | EZR | PARP1 |  |  |
|  | PRDX1 | RPS27A |  |  |
|  | PDAP1 | SNRPG |  |  |
|  | S100A6 | SLC20A1 |  |  |
|  | MRPL47 | CSRNP1 |  |  |
|  | NUPR1 | RPL32 |  |  |
|  | NDUFB5 | RPL37 |  |  |
|  | UQCRH | PHIP |  |  |
|  | DGUOK | PNRC1 |  |  |
|  | RPL14 | ARHGAP18 |  |  |
|  | COX8A | GPC3 |  |  |
|  | PLSCR1 | CETN2 |  |  |
|  | SPARCL1 | RPL10 |  |  |
|  | CBR1 | RPL7 |  |  |
|  | TPI1 | NTRK2 |  |  |
|  | UFD1L | UGCG |  |  |
|  | JUN | RPL7A |  |  |
|  | TALDO1 | ADM |  |  |
|  | AHCY | CELF1 |  |  |
|  | SAT1 | RPS3 |  |  |
|  | RPL10A | ALDOA |  |  |
|  | FBL | AKR1C2 |  |  |
|  | RPL7 | BAG3 |  |  |
|  | RPS3 | MZT2B |  |  |
|  | BSG | ZFP36L2 |  |  |
|  | MRPL40 | HNRNPU |  |  |
|  | PDCD10 | MSI2 |  |  |
|  | CRYAB | CXADR |  |  |
|  | TMC5 | KLF10 |  |  |
|  | MZT2B | AZIN1 |  |  |
|  | WSB1 | UBE2L6 |  |  |
|  | DNAJA4 | RAB11FIP1 |  |  |
|  | METTL12 | MRPL17 |  |  |
|  | COMT | PINK1 |  |  |
|  | RPLP1 | SON |  |  |
|  | CYR61 | HK2 |  |  |
|  | RRAD | G6PD |  |  |
|  | RBCK1 | CSTB |  |  |
|  | PPIA | RPL8 |  |  |
|  | LDLR | YDJC |  |  |
|  | FOSB | RPL26 |  |  |
|  | NDUFA4L2 | RPL29 |  |  |
|  | PDK4 | ZNF281 |  |  |
|  | ARPC1B | CAPN2 |  |  |
|  | TOB1 | BOLA3 |  |  |
|  | JUNB | S100A11 |  |  |
|  | SNHG25 | DAPL1 |  |  |
|  | ATP1B3 | HIPK1 |  |  |
|  | SNRPG | IFI16 |  |  |
|  | CCDC85B | RPL22L1 |  |  |
|  | POLR1D | CDS1 |  |  |
|  | AKR1C1 | YEATS2 |  |  |
|  | CRIP1 | TKT |  |  |
|  | UCHL1 | SGMS2 |  |  |
|  | MRPS21 | TRA2A |  |  |
|  | ENO1 | RPS14 |  |  |
|  | MLF2 | ALDH1A1 |  |  |
|  | PTHLH | SPTSSA |  |  |
|  | EEF2 | RRAD |  |  |
|  | BOLA3 | B2M |  |  |
|  | SCAND1 | MIDN |  |  |
|  | ARFGEF3 | RPL13 |  |  |
|  | CYP4B1 | EEF2 |  |  |
|  | COPS6 | COPS6 |  |  |
|  | RPS17 | ARF4 |  |  |
|  | SFPQ | STAT3 |  |  |
|  | EIF2S2 | SLC25A6 |  |  |
|  | SNRPD2 | KIF5B |  |  |
|  | MZT2A | C9orf16 |  |  |
|  | EEF1D | SCAND1 |  |  |
|  | ZFP36 | PGAM1 |  |  |
|  | RAB11FIP1 | KRT19 |  |  |
|  | AZIN1 | RPS7 |  |  |
|  | CPEB4 | JMJD1C |  |  |
|  | MRPL21 | BSG |  |  |
|  | SLC7A2 | RPL38 |  |  |
|  | ZFAND5 | MZT2A |  |  |
|  | CYB5A | TRIB1 |  |  |
|  | ARPC1A | NUDT4 |  |  |
|  | PSMD8 | UQCRH |  |  |
|  | CFLAR | PLK3 |  |  |
|  | NR4A2 | FAM174A |  |  |
|  | HSPA1B | ATP2A2 |  |  |
|  | TCEAL4 | RPL15 |  |  |
|  | FOS | PARL |  |  |
|  | UGCG | SFN |  |  |
|  | PPP1R15A | COX8A |  |  |
|  | SGMS2 | TALDO1 |  |  |
|  | PSMD7 | GBA |  |  |
|  | KRT17 | RPS27 |  |  |
|  | SLIRP | KCTD12 |  |  |
|  | TSC22D1 | EIF3K |  |  |
|  | CLDN4 | NPM1 |  |  |
|  | PHB2 | RPS17 |  |  |
|  | HSPH1 | RPL35A |  |  |
|  | NPM1 | CIB1 |  |  |
|  | FAM133B | MRPL40 |  |  |
|  | KCTD12 | NDUFA4L2 |  |  |
|  | EIF6 | ZFP36L1 |  |  |
|  | PLP2 | UBE2L3 |  |  |
|  | BUD31 | POLR1D |  |  |
|  | STAT3 | RPS23 |  |  |
|  | XBP1 | INSIG1 |  |  |
|  | DNAAF1 | UBE2H |  |  |
|  | SPAG6 | HEXIM1 |  |  |
|  | SDC4 | DYNC2H1 |  |  |
|  | PLA2G16 | TPRG1 |  |  |
|  | SON | LAMTOR4 |  |  |
|  | CEBPB | RPL14 |  |  |
|  | SLC25A1 | LRRK2 |  |  |
|  | CSRNP1 | CFAP126 |  |  |
|  | FAM216B | PPIA |  |  |
|  | PFDN2 | NACA |  |  |
|  | MT1X | TOMM7 |  |  |
|  | PNRC1 | S100A2 |  |  |
|  | ETS2 | SRC |  |  |
|  | LMO7 | NEK5 |  |  |
|  | SLC5A3 | SERPINA1 |  |  |
|  | TMEM190 | MRPL21 |  |  |
|  | SLC2A1 | RPL12 |  |  |
|  | PGK1 | RPS4X |  |  |
|  | DNAH12 | AKR1B10 |  |  |
|  | UQCC3 | CALM1 |  |  |
|  | AGR3 | RPL10A |  |  |
|  | SOX4 | RPL39 |  |  |
|  | OMG | APRT |  |  |
|  | CFAP157 | BRD2 |  |  |
|  | CCDC170 | HSPA1B |  |  |
|  | C11orf97 | HLA-C |  |  |
|  | FAM92B | RACK1 |  |  |
|  | MS4A8 | UQCC3 |  |  |
|  | PKIB | KRT6A |  |  |
|  | RANBP1 | DNAJC19 |  |  |
|  | FAM46A | ANKRD28 |  |  |
|  | FAM162A | PHB2 |  |  |
|  | RP4-666F24.3 | DDX3X |  |  |
|  | DAPL1 | UBA52 |  |  |
|  | EIF5 | PRR29 |  |  |
|  | MTRNR2L12 | RPS18 |  |  |
|  | SOCS3 | RPS28 |  |  |
|  | IFT57 | FAM133B |  |  |
|  | SPATA18 | HLA-B |  |  |
|  | RARRES3 | RPL36A |  |  |
|  | C19orf60 | ARPC1A |  |  |
|  | CCNL1 | C22orf39 |  |  |
|  | TRIB1 | EIF6 |  |  |
|  | ICAM1 | HBB |  |  |
|  | SLC44A4 | MRPS21 |  |  |
|  | INTS6 | MARCKS |  |  |
|  | SH3BGRL3 | DLD |  |  |
|  | KLHL24 | NFE2L2 |  |  |
|  | CES1 | NLRP3 |  |  |
|  | NQO1 | MTF1 |  |  |
|  | CATSPERD | DLST |  |  |
|  | KCNQ1OT1 | PDHA1 |  |  |
|  | CLIC6 | SLC31A1 |  |  |
|  | ADIRF | FDX1 |  |  |
|  | NEK5 | DBT |  |  |
|  | ARRDC3 | LIPT1 |  |  |
|  | STEAP4 | DLAT |  |  |
|  | HNRNPU | PDHB |  |  |
|  | RPL3 | LIPT2 |  |  |
|  | CD9 | ATP7B |  |  |
|  | PGAM1 | ATP7A |  |  |
|  | DNAJB6 | LIAS |  |  |
|  | ERRFI1 | GCSH |  |  |
|  | DNAJA1 |  |  |  |
|  | FAM183A |  |  |  |
|  | B2M |  |  |  |
|  | GPRC5A |  |  |  |
|  | CCDC78 |  |  |  |
|  | AC013264.2 |  |  |  |
|  | CCDC17 |  |  |  |
|  | C9orf24 |  |  |  |
|  | PTGES3 |  |  |  |
|  | SPAG17 |  |  |  |
|  | GADD45B |  |  |  |
|  | CFAP43 |  |  |  |
|  | CELF1 |  |  |  |
|  | C6orf62 |  |  |  |
|  | TRA2B |  |  |  |
|  | ZC3H12A |  |  |  |
|  | SHROOM3 |  |  |  |
|  | ALDH3B1 |  |  |  |
|  | LINC01206 |  |  |  |
|  | ZMYND10 |  |  |  |
|  | ALOX15 |  |  |  |
|  | NEK10 |  |  |  |
|  | C20orf85 |  |  |  |
|  | DNAH5 |  |  |  |
|  | VPS37B |  |  |  |
|  | DNAH7 |  |  |  |
|  | GPI |  |  |  |
|  | HEXIM1 |  |  |  |
|  | SRGAP3-AS2 |  |  |  |
|  | PERP |  |  |  |
|  | CDHR3 |  |  |  |
|  | SNRPB |  |  |  |
|  | MUC15 |  |  |  |
|  | C9orf135 |  |  |  |
|  | LRRC46 |  |  |  |
|  | WDR38 |  |  |  |
|  | IDI1 |  |  |  |
|  | C5orf49 |  |  |  |
|  | ANKRD28 |  |  |  |
|  | C11orf88 |  |  |  |
|  | RP11-356K23.1 |  |  |  |
|  | SMIM22 |  |  |  |
|  | SRSF3 |  |  |  |
|  | AREG |  |  |  |
|  | KIF5B |  |  |  |
|  | C1orf194 |  |  |  |
|  | NET1 |  |  |  |
|  | MALAT1 |  |  |  |
|  | RP1 |  |  |  |
|  | NUFIP2 |  |  |  |
|  | C22orf15 |  |  |  |
|  | TSPAN19 |  |  |  |
|  | CAPSL |  |  |  |
|  | ODF2L |  |  |  |
|  | MAPK15 |  |  |  |
|  | FXYD1 |  |  |  |
|  | DRC3 |  |  |  |
|  | POMP |  |  |  |
|  | PRR29 |  |  |  |
|  | SERTAD1 |  |  |  |
|  | ROPN1L |  |  |  |
|  | RSPH1 |  |  |  |
|  | SFTPB |  |  |  |
|  | PSCA |  |  |  |
|  | SYNE1 |  |  |  |
|  | DNAJB1 |  |  |  |
|  | ZFP36L2 |  |  |  |
|  | WEE1 |  |  |  |
|  | NEDD9 |  |  |  |
|  | CCDC146 |  |  |  |
|  | HSPA8 |  |  |  |
|  | ERICH3 |  |  |  |
|  | KLF10 |  |  |  |
|  | DYNLRB2 |  |  |  |
|  | STOML3 |  |  |  |
|  | LDLRAD1 |  |  |  |
|  | ZBBX |  |  |  |
|  | RSPH9 |  |  |  |
|  | UBXN10 |  |  |  |
|  | MORN5 |  |  |  |
|  | TEKT1 |  |  |  |
|  | EHF |  |  |  |
|  | ZBTB10 |  |  |  |
|  | IER5 |  |  |  |
|  | NUDT4 |  |  |  |
|  | LRRFIP1 |  |  |  |
|  | VSTM2L |  |  |  |
|  | CCDC153 |  |  |  |
|  | MKNK2 |  |  |  |
|  | TRA2A |  |  |  |
|  | MLLT4 |  |  |  |
|  | NEAT1 |  |  |  |
|  | HIPK1 |  |  |  |
|  | SNTN |  |  |  |
|  | PRRG4 |  |  |  |
|  | SAXO2 |  |  |  |
|  | ADM |  |  |  |
|  | IRX2 |  |  |  |
|  | PRR4 |  |  |  |
|  | FUS |  |  |  |
|  | TEKT2 |  |  |  |
|  | FOXP1 |  |  |  |
|  | C2orf40 |  |  |  |
|  | BAG3 |  |  |  |
|  | DNAH9 |  |  |  |
|  | HBB |  |  |  |
|  | PRKAR1A |  |  |  |
|  | CFAP53 |  |  |  |
|  | RFX3 |  |  |  |
|  | INSIG1 |  |  |  |
|  | MUC4 |  |  |  |
|  | DDIT4 |  |  |  |
|  | AKAP9 |  |  |  |
|  | IER2 |  |  |  |
|  | 6-Mar |  |  |  |
|  | CFAP52 |  |  |  |
|  | CFAP70 |  |  |  |
|  | CAPN2 |  |  |  |
|  | PCM1 |  |  |  |
|  | NR4A1 |  |  |  |
|  | BAIAP2 |  |  |  |
|  | IRF1 |  |  |  |
|  | TLE4 |  |  |  |
|  | DNAH11 |  |  |  |
|  | ARF4 |  |  |  |
|  | ABHD2 |  |  |  |
|  | MRPS31 |  |  |  |
|  | PLK3 |  |  |  |
|  | CFAP126 |  |  |  |
|  | MCL1 |  |  |  |
|  | C9orf116 |  |  |  |
|  | DUSP6 |  |  |  |
|  | DUOX1 |  |  |  |
|  | CD164L2 |  |  |  |
|  | KRT19 |  |  |  |
|  | FAM81B |  |  |  |
|  | ARMC3 |  |  |  |
|  | CXADR |  |  |  |
|  | CLDN3 |  |  |  |
|  | AC006262.5 |  |  |  |
|  | GLS |  |  |  |
|  | STK33 |  |  |  |
|  | CTGF |  |  |  |
|  | MTRNR2L8 |  |  |  |
|  | EPHA2 |  |  |  |
|  | PHIP |  |  |  |
|  | HYDIN |  |  |  |
|  | LRRIQ1 |  |  |  |
|  | SQLE |  |  |  |
|  | ATF7IP2 |  |  |  |
|  | MNS1 |  |  |  |
|  | JMJD1C |  |  |  |
|  | CXCL2 |  |  |  |
|  | DYNLL1 |  |  |  |
|  | ATP2A2 |  |  |  |
|  | IQCG |  |  |  |
|  | CEP126 |  |  |  |
|  | PIFO |  |  |  |
|  | CDS1 |  |  |  |
|  | CD24 |  |  |  |
|  | KLF5 |  |  |  |
|  | SRSF7 |  |  |  |
|  | DYNC2H1 |  |  |  |
|  | SPP1 |  |  |  |
|  | ENKUR |  |  |  |
|  | CKB |  |  |  |
|  | RSPH3 |  |  |  |
|  | PLAC8 |  |  |  |
|  | EFHC1 |  |  |  |
|  | ALCAM |  |  |  |
|  | CCND1 |  |  |  |
|  | KIF9 |  |  |  |
|  | EGR1 |  |  |  |
|  | UBE2H |  |  |  |
|  | SRI |  |  |  |
|  | IGFBP5 |  |  |  |
|  | SPEF2 |  |  |  |
|  | NUCB2 |  |  |  |
|  | FOXJ1 |  |  |  |
|  | HNRNPU-AS1 |  |  |  |
|  | POLR2J3 |  |  |  |
|  | CCL20 |  |  |  |
|  | CXCL8 |  |  |  |
|  | HES1 |  |  |  |
|  | HMGCS1 |  |  |  |
|  | PROS1 |  |  |  |
|  | UBXN11 |  |  |  |
|  | LYZ |  |  |  |
|  | GSTA1 |  |  |  |
|  | RND1 |  |  |  |
|  | TMEM231 |  |  |  |
|  | SARAF |  |  |  |
|  | CCDC74A |  |  |  |
|  | SERPINA1 |  |  |  |
|  | GEM |  |  |  |
|  | CD164 |  |  |  |
|  | IER3 |  |  |  |
|  | EFCAB1 |  |  |  |
|  | TNFAIP3 |  |  |  |
|  | CD83 |  |  |  |
|  | PMAIP1 |  |  |  |
|  | GSTA2 |  |  |  |
|  | ANXA1 |  |  |  |
|  | SCGB3A1 |  |  |  |
|  | ZNF281 |  |  |  |
|  | C8orf4 |  |  |  |
|  | RP11-295M3.4 |  |  |  |
|  | LBH |  |  |  |
|  | ARHGAP18 |  |  |  |
|  | TREM1 |  |  |  |
|  | CH17-340M24.3 |  |  |  |
|  | SLC20A1 |  |  |  |
|  | CETN2 |  |  |  |
|  | HMGN3 |  |  |  |
|  | MSI2 |  |  |  |
|  | CIB1 |  |  |  |
|  | TCTEX1D2 |  |  |  |
|  | CARS |  |  |  |
|  | ALDH1A1 |  |  |  |
|  | CHST9 |  |  |  |
|  | RABL2B |  |  |  |
|  | CAPS |  |  |  |
|  | METTL7A |  |  |  |
|  | KIF21A |  |  |  |
|  | TFF3 |  |  |  |
|  | LRRC23 |  |  |  |
|  | SOD2 |  |  |  |
|  | FAM229B |  |  |  |
|  | UPP1 |  |  |  |
|  | BPIFA1 |  |  |  |
|  | FAM174A |  |  |  |
|  | SFTPA2 |  |  |  |
|  | CALM1 |  |  |  |
|  | CXCL3 |  |  |  |
|  | SAA2 |  |  |  |
|  | SAA1 |  |  |  |
|  | LY6E |  |  |  |
|  | TPPP3 |  |  |  |
|  | S100A9 |  |  |  |
|  | SPAG16 |  |  |  |
|  | SFTPC |  |  |  |
|  | TSPAN1 |  |  |  |
|  | MARCKS |  |  |  |

Supplementary Table 2. Primer sequences for experimental validation. List the corresponding qPCR primer sequences for each gene used for experimental validation, including the specific sequences of forward and reverse primers and their key parameters.

|  | Sequence (5' -> 3') | Length | Tm | Location |
| --- | --- | --- | --- | --- |
| Forward Primer | CTAAAACCCGCCGGACTTTCT | 21 | 62.3 | 14-34 |
| Reverse Primer | CTTCCTGTCATAACGCCGCTT | 21 | 62.7 | 132-112 |

Supplementary Table 3. Correlation analysis of cuproptosis with mitochondrial depolarizing genes and differentially expressed genes. Demonstrate the strength of statistical associations between cuproptosis, mitochondrial depolarizing genes and differentially expressed genes and assess possible interactions or effects between them.

|  | gene1 | gene2 | pearson |
| --- | --- | --- | --- |
| 1 | GLS | GLS | 1 |
| 2 | CDKN2A | CDKN2A | 1 |
| 3 | DLD | GCLC | 0.527047514 |
| 4 | NFE2L2 | GCLC | 0.549989117 |
| 5 | GLS | CFLAR | 0.576567375 |
| 6 | NLRP3 | CFLAR | 0.67440547 |
| 7 | MTF1 | CFLAR | 0.548503118 |
| 8 | NLRP3 | PDK4 | 0.589761481 |
| 9 | DLD | SLC25A5 | 0.670525505 |
| 10 | NFE2L2 | SLC25A5 | 0.561898258 |
| 11 | DLST | SLC25A5 | 0.637458529 |
| 12 | PDHA1 | SLC25A5 | 0.650888201 |
| 13 | SLC31A1 | SLC25A5 | 0.536484173 |
| 14 | FDX1 | SLC25A5 | 0.50775474 |
| 15 | DBT | SLC25A5 | 0.529874869 |
| 16 | LIPT1 | SLC25A5 | 0.517997679 |
| 17 | DLAT | SLC25A5 | 0.579735316 |
| 18 | PDHB | SLC25A5 | 0.532318981 |
| 19 | LIPT2 | SLC25A5 | 0.564793103 |
| 20 | DLD | POLR2J | 0.656905645 |
| 21 | DLST | POLR2J | 0.533293453 |
| 22 | PDHA1 | POLR2J | 0.669193976 |
| 23 | PDHB | POLR2J | 0.540140396 |
| 24 | DLD | CD9 | 0.557433795 |
| 25 | NFE2L2 | CD9 | 0.723249081 |
| 26 | NLRP3 | DCN | 0.562905028 |
| 27 | DLD | GCLM | 0.612373973 |
| 28 | NFE2L2 | GCLM | 0.57506623 |
| 29 | PDHB | TMSB10 | 0.578054154 |
| 30 | DLD | THOC3 | 0.598305652 |
| 31 | NFE2L2 | THOC3 | 0.623622184 |
| 32 | PDHA1 | THOC3 | 0.634818732 |
| 33 | LIPT2 | THOC3 | 0.568651708 |
| 34 | DLD | RPL18 | 0.502963929 |
| 35 | DLST | RPL18 | 0.599559487 |
| 36 | PDHA1 | RPL18 | 0.589284204 |
| 37 | PDHB | RPL18 | 0.597537758 |
| 38 | NFE2L2 | GPC1 | 0.672770136 |
| 39 | DLD | IDI1 | 0.525969061 |
| 40 | DLST | IDI1 | 0.539631221 |
| 41 | SLC31A1 | IDI1 | 0.537136546 |
| 42 | FDX1 | IDI1 | 0.554936547 |
| 43 | PDHB | IDI1 | 0.572083997 |
| 44 | NLRP3 | KLF6 | 0.661339239 |
| 45 | DLD | ATP1B3 | 0.542988731 |
| 46 | NFE2L2 | ATP1B3 | 0.744675223 |
| 47 | PDHA1 | ATP1B3 | 0.522163934 |
| 48 | PDHB | NUCB2 | 0.573672634 |
| 49 | DLST | RPL31 | 0.545872343 |
| 50 | FDX1 | RPL31 | 0.52952596 |
| 51 | LIPT1 | RPL31 | 0.639506251 |
| 52 | PDHB | RPL31 | 0.65884345 |
| 53 | DLD | ENO1 | 0.533662886 |
| 54 | DLST | ENO1 | 0.584891037 |
| 55 | PDHA1 | ENO1 | 0.627777617 |
| 56 | DLAT | ENO1 | 0.566709244 |
| 57 | DLST | SRI | 0.505633463 |
| 58 | FDX1 | SRI | 0.559620195 |
| 59 | LIPT1 | SRI | 0.556165448 |
| 60 | PDHB | SRI | 0.632740304 |
| 61 | ATP7B | PCM1 | 0.54511664 |
| 62 | ATP7A | PCM1 | 0.530592044 |
| 63 | PDHB | PCM1 | 0.519567417 |
| 64 | MTF1 | PCM1 | 0.587806493 |
| 65 | DLD | HSP90AA1 | 0.697020103 |
| 66 | NFE2L2 | HSP90AA1 | 0.522947788 |
| 67 | DLST | HSP90AA1 | 0.728429663 |
| 68 | PDHA1 | HSP90AA1 | 0.657121539 |
| 69 | SLC31A1 | HSP90AA1 | 0.513940608 |
| 70 | DBT | HSP90AA1 | 0.562113267 |
| 71 | DLAT | HSP90AA1 | 0.616468843 |
| 72 | PDHB | HSP90AA1 | 0.536019396 |
| 73 | NLRP3 | CXCL2 | 0.538561796 |
| 74 | PDHA1 | CTTN | 0.525995368 |
| 75 | DLD | DNAJA1 | 0.552096915 |
| 76 | DLST | DNAJA1 | 0.582131322 |
| 77 | SLC31A1 | DNAJA1 | 0.610876828 |
| 78 | FDX1 | DNAJA1 | 0.51263711 |
| 79 | DLAT | DNAJA1 | 0.556085436 |
| 80 | PDHB | DNAJA1 | 0.629308776 |
| 81 | DLD | HUWE1 | 0.551626454 |
| 82 | PDHA1 | HUWE1 | 0.620902233 |
| 83 | DBT | HUWE1 | 0.521832571 |
| 84 | DLAT | HUWE1 | 0.618420229 |
| 85 | ATP7A | HUWE1 | 0.641234276 |
| 86 | MTF1 | HUWE1 | 0.735345965 |
| 87 | SLC31A1 | FTL | 0.513435662 |
| 88 | PDHB | FTL | 0.505953924 |
| 89 | NFE2L2 | PTHLH | 0.571507783 |
| 90 | DLD | PIR | 0.571638707 |
| 91 | NFE2L2 | PIR | 0.608239414 |
| 92 | PDHA1 | PIR | 0.604757623 |
| 93 | LIPT2 | PIR | 0.613143853 |
| 94 | FDX1 | KIF9 | 0.503211694 |
| 95 | PDHB | KIF9 | 0.672858014 |
| 96 | DLD | DYNLL1 | 0.668128777 |
| 97 | NFE2L2 | DYNLL1 | 0.556199581 |
| 98 | DLST | DYNLL1 | 0.66007957 |
| 99 | PDHA1 | DYNLL1 | 0.677726014 |
| 100 | DBT | DYNLL1 | 0.512792734 |
| 101 | LIPT1 | DYNLL1 | 0.508700597 |
| 102 | DLAT | DYNLL1 | 0.555672992 |
| 103 | PDHB | DYNLL1 | 0.558868012 |
| 104 | LIPT2 | DYNLL1 | 0.5427266 |
| 105 | DLD | RPL6 | 0.620299822 |
| 106 | DLST | RPL6 | 0.596771923 |
| 107 | LIAS | RPL6 | 0.511366128 |
| 108 | PDHA1 | RPL6 | 0.67811494 |
| 109 | DBT | RPL6 | 0.511411267 |
| 110 | LIPT1 | RPL6 | 0.531990016 |
| 111 | DLAT | RPL6 | 0.519160071 |
| 112 | PDHB | RPL6 | 0.633720572 |
| 113 | NLRP3 | P2RX7 | 0.769561914 |
| 114 | DLST | FUS | 0.537055445 |
| 115 | PDHA1 | FUS | 0.680940205 |
| 116 | GCSH | FUS | 0.505787948 |
| 117 | MTF1 | FUS | 0.566344755 |
| 118 | NFE2L2 | FXYD3 | 0.582153784 |
| 119 | DLD | MLF2 | 0.636413146 |
| 120 | NFE2L2 | MLF2 | 0.549058595 |
| 121 | DLST | MLF2 | 0.551849713 |
| 122 | PDHA1 | MLF2 | 0.725578773 |
| 123 | LIPT2 | MLF2 | 0.54929952 |
| 124 | GLS | ICAM1 | 0.51682236 |
| 125 | NLRP3 | ICAM1 | 0.687009995 |
| 126 | NLRP3 | LYZ | 0.71685525 |
| 127 | DLST | MUL1 | 0.67914069 |
| 128 | PDHA1 | MUL1 | 0.516742543 |
| 129 | SLC31A1 | MUL1 | 0.581519226 |
| 130 | FDX1 | MUL1 | 0.579178812 |
| 131 | DLAT | MUL1 | 0.568503711 |
| 132 | PDHB | MUL1 | 0.616241407 |
| 133 | MTF1 | MUL1 | 0.616283285 |
| 134 | DLST | PPP2R3C | 0.583117972 |
| 135 | SLC31A1 | PPP2R3C | 0.556460523 |
| 136 | FDX1 | PPP2R3C | 0.503560594 |
| 137 | LIPT1 | PPP2R3C | 0.531553073 |
| 138 | DLAT | PPP2R3C | 0.506719509 |
| 139 | PDHB | PPP2R3C | 0.620986431 |
| 140 | DLST | EZR | 0.501257998 |
| 141 | FDX1 | EZR | 0.515520781 |
| 142 | PDHB | EZR | 0.50433778 |
| 143 | MTF1 | EZR | 0.543240453 |
| 144 | DLD | COMT | 0.511626274 |
| 145 | DLST | COMT | 0.561958965 |
| 146 | PDHA1 | COMT | 0.552248679 |
| 147 | FDX1 | COMT | 0.512498282 |
| 148 | PDHB | COMT | 0.527439588 |
| 149 | DLST | HDAC6 | 0.51050258 |
| 150 | PDHA1 | HDAC6 | 0.651623961 |
| 151 | DLAT | HDAC6 | 0.501313507 |
| 152 | PDHB | HDAC6 | 0.528690581 |
| 153 | MTF1 | HDAC6 | 0.613256323 |
| 154 | DLD | ABL1 | 0.511610682 |
| 155 | DLST | ABL1 | 0.583821374 |
| 156 | PDHA1 | ABL1 | 0.561983462 |
| 157 | SLC31A1 | ABL1 | 0.550147746 |
| 158 | DLAT | ABL1 | 0.514791401 |
| 159 | PDHB | ABL1 | 0.521815815 |
| 160 | MTF1 | ABL1 | 0.657448461 |
| 161 | DLD | PSMD8 | 0.588186801 |
| 162 | DLST | PSMD8 | 0.581509103 |
| 163 | PDHA1 | PSMD8 | 0.615256647 |
| 164 | DLAT | PSMD8 | 0.542665998 |
| 165 | PDHB | PSMD8 | 0.540218429 |
| 166 | LIPT2 | PSMD8 | 0.508929836 |
| 167 | NLRP3 | GADD45B | 0.610185725 |
| 168 | DLST | MKNK2 | 0.506009682 |
| 169 | PDHA1 | MKNK2 | 0.514798575 |
| 170 | MTF1 | MKNK2 | 0.571077464 |
| 171 | DLD | RANBP1 | 0.644225404 |
| 172 | NFE2L2 | RANBP1 | 0.559408905 |
| 173 | DLST | RANBP1 | 0.502776045 |
| 174 | PDHA1 | RANBP1 | 0.682178376 |
| 175 | DLAT | RANBP1 | 0.510608789 |
| 176 | LIPT2 | RANBP1 | 0.573120009 |
| 177 | DLD | SLC25A1 | 0.580355346 |
| 178 | NFE2L2 | SLC25A1 | 0.521391569 |
| 179 | DLST | SLC25A1 | 0.55324142 |
| 180 | PDHA1 | SLC25A1 | 0.679635608 |
| 181 | LIPT2 | SLC25A1 | 0.508925444 |
| 182 | PDHB | XBP1 | 0.535416277 |
| 183 | DLST | TSPO | 0.502755274 |
| 184 | DLD | RPL3 | 0.529597756 |
| 185 | DLST | RPL3 | 0.640110837 |
| 186 | PDHA1 | RPL3 | 0.572422154 |
| 187 | FDX1 | RPL3 | 0.513225115 |
| 188 | DBT | RPL3 | 0.507281836 |
| 189 | LIPT1 | RPL3 | 0.506481573 |
| 190 | PDHB | RPL3 | 0.674242391 |
| 191 | DLD | EIF5 | 0.673111465 |
| 192 | NFE2L2 | EIF5 | 0.643447249 |
| 193 | DLST | EIF5 | 0.740811307 |
| 194 | PDHA1 | EIF5 | 0.579893327 |
| 195 | SLC31A1 | EIF5 | 0.568848842 |
| 196 | DBT | EIF5 | 0.621902631 |
| 197 | LIPT1 | EIF5 | 0.526024979 |
| 198 | DLAT | EIF5 | 0.614584342 |
| 199 | ATP7A | EIF5 | 0.507163606 |
| 200 | PDHB | EIF5 | 0.512838651 |
| 201 | LIPT2 | EIF5 | 0.532403827 |
| 202 | MTF1 | EIF5 | 0.540643078 |
| 203 | DLST | PSME2 | 0.551723414 |
| 204 | PDHA1 | PSME2 | 0.504183598 |
| 205 | PDHB | PSME2 | 0.55358647 |
| 206 | DLD | AHCY | 0.672383754 |
| 207 | NFE2L2 | AHCY | 0.501282702 |
| 208 | DLST | AHCY | 0.582201356 |
| 209 | PDHA1 | AHCY | 0.678187535 |
| 210 | DBT | AHCY | 0.505608803 |
| 211 | DLAT | AHCY | 0.583252913 |
| 212 | PDHB | AHCY | 0.506420159 |
| 213 | LIPT2 | AHCY | 0.586664725 |
| 214 | PDHA1 | ABCD1 | 0.563069106 |
| 215 | DLD | PLP2 | 0.545205201 |
| 216 | NFE2L2 | PLP2 | 0.528636764 |
| 217 | DLST | PLP2 | 0.511737211 |
| 218 | PDHA1 | PLP2 | 0.619260537 |
| 219 | DLD | PGK1 | 0.627904686 |
| 220 | DLST | PGK1 | 0.583358638 |
| 221 | PDHA1 | PGK1 | 0.576632572 |
| 222 | DLAT | PGK1 | 0.579486788 |
| 223 | DLD | KLF5 | 0.537327433 |
| 224 | NFE2L2 | KLF5 | 0.734379638 |
| 225 | DLD | MRPS31 | 0.560214111 |
| 226 | DLST | MRPS31 | 0.618955927 |
| 227 | LIAS | MRPS31 | 0.578914258 |
| 228 | PDHA1 | MRPS31 | 0.583774203 |
| 229 | FDX1 | MRPS31 | 0.572598181 |
| 230 | DBT | MRPS31 | 0.595071059 |
| 231 | LIPT1 | MRPS31 | 0.664414826 |
| 232 | DLAT | MRPS31 | 0.550483461 |
| 233 | PDHB | MRPS31 | 0.677253587 |
| 234 | LIPT2 | MRPS31 | 0.507709543 |
| 235 | DLD | INTS6 | 0.587911351 |
| 236 | NFE2L2 | INTS6 | 0.532041312 |
| 237 | DLST | INTS6 | 0.501566275 |
| 238 | DBT | INTS6 | 0.575158944 |
| 239 | DLAT | INTS6 | 0.572955188 |
| 240 | ATP7A | INTS6 | 0.507661575 |
| 241 | MTF1 | INTS6 | 0.533341979 |
| 242 | PDHB | TSC22D1 | 0.541485322 |
| 243 | DLD | PSMD7 | 0.703664703 |
| 244 | NFE2L2 | PSMD7 | 0.574342825 |
| 245 | DLST | PSMD7 | 0.662431881 |
| 246 | PDHA1 | PSMD7 | 0.659592192 |
| 247 | SLC31A1 | PSMD7 | 0.54019166 |
| 248 | DBT | PSMD7 | 0.579128986 |
| 249 | DLAT | PSMD7 | 0.691449816 |
| 250 | PDHB | PSMD7 | 0.506500205 |
| 251 | LIPT2 | PSMD7 | 0.573792645 |
| 252 | DLST | EEF1D | 0.614730687 |
| 253 | LIAS | EEF1D | 0.528321962 |
| 254 | PDHA1 | EEF1D | 0.604580399 |
| 255 | DLAT | EEF1D | 0.507909141 |
| 256 | PDHB | EEF1D | 0.548424722 |
| 257 | DLD | SQLE | 0.530455053 |
| 258 | NFE2L2 | SQLE | 0.50756957 |
| 259 | PDHA1 | SQLE | 0.567191274 |
| 260 | DLD | PDCD5 | 0.606117309 |
| 261 | PDHA1 | PDCD5 | 0.659312896 |
| 262 | GCSH | PDCD5 | 0.526762427 |
| 263 | LIPT2 | PDCD5 | 0.515610488 |
| 264 | DLST | RPS16 | 0.533439025 |
| 265 | PDHA1 | RPS16 | 0.519006671 |
| 266 | PDHB | RPS16 | 0.563966488 |
| 267 | DLD | FBL | 0.605522835 |
| 268 | NFE2L2 | FBL | 0.544132721 |
| 269 | PDHA1 | FBL | 0.670483429 |
| 270 | LIPT2 | FBL | 0.541454214 |
| 271 | DLD | GPI | 0.653058039 |
| 272 | NFE2L2 | GPI | 0.580394497 |
| 273 | PDHA1 | GPI | 0.688470437 |
| 274 | LIPT2 | GPI | 0.554090634 |
| 275 | DLD | RPS19 | 0.516648001 |
| 276 | DLST | RPS19 | 0.530133246 |
| 277 | PDHA1 | RPS19 | 0.588445548 |
| 278 | PDHB | RPS19 | 0.516936659 |
| 279 | DLD | CDC37 | 0.525719193 |
| 280 | DLST | CDC37 | 0.604047375 |
| 281 | PDHA1 | CDC37 | 0.65921792 |
| 282 | PDHB | CDC37 | 0.5552523 |
| 283 | MTF1 | CDC37 | 0.506552705 |
| 284 | DLD | RPL18A | 0.50311584 |
| 285 | DLST | RPL18A | 0.526152279 |
| 286 | PDHA1 | RPL18A | 0.516419925 |
| 287 | LIPT1 | RPL18A | 0.502299112 |
| 288 | PDHB | RPL18A | 0.556202116 |
| 289 | DLD | DNAJB6 | 0.686995143 |
| 290 | NFE2L2 | DNAJB6 | 0.557662112 |
| 291 | DLST | DNAJB6 | 0.570121343 |
| 292 | PDHA1 | DNAJB6 | 0.59861455 |
| 293 | DBT | DNAJB6 | 0.554717503 |
| 294 | DLAT | DNAJB6 | 0.555655073 |
| 295 | MTF1 | DNAJB6 | 0.504554617 |
| 296 | DLD | PDAP1 | 0.731270184 |
| 297 | NFE2L2 | PDAP1 | 0.508510537 |
| 298 | DLST | PDAP1 | 0.588056013 |
| 299 | PDHA1 | PDAP1 | 0.750691601 |
| 300 | DLAT | PDAP1 | 0.557444813 |
| 301 | DLD | BUD31 | 0.723317558 |
| 302 | DLST | BUD31 | 0.602866112 |
| 303 | PDHA1 | BUD31 | 0.631092452 |
| 304 | FDX1 | BUD31 | 0.507966363 |
| 305 | DBT | BUD31 | 0.508898323 |
| 306 | LIPT1 | BUD31 | 0.553047312 |
| 307 | DLAT | BUD31 | 0.549315848 |
| 308 | PDHB | BUD31 | 0.597085066 |
| 309 | LIPT2 | BUD31 | 0.525571301 |
| 310 | DLD | ZNHIT1 | 0.516214892 |
| 311 | LIPT1 | ZNHIT1 | 0.526943368 |
| 312 | PDHB | ZNHIT1 | 0.604571239 |
| 313 | NFE2L2 | TLE4 | 0.528237026 |
| 314 | SLC31A1 | ZFAND5 | 0.586925573 |
| 315 | ATP7A | ZFAND5 | 0.536543325 |
| 316 | PDHB | ZFAND5 | 0.558282488 |
| 317 | DLD | NUFIP2 | 0.549479189 |
| 318 | DBT | NUFIP2 | 0.549701491 |
| 319 | DLAT | NUFIP2 | 0.580018131 |
| 320 | ATP7A | NUFIP2 | 0.656673343 |
| 321 | MTF1 | NUFIP2 | 0.643746129 |
| 322 | DLD | RPL19 | 0.571754774 |
| 323 | DLST | RPL19 | 0.580240043 |
| 324 | LIAS | RPL19 | 0.510914685 |
| 325 | PDHA1 | RPL19 | 0.625875956 |
| 326 | FDX1 | RPL19 | 0.523230073 |
| 327 | LIPT1 | RPL19 | 0.572330381 |
| 328 | PDHB | RPL19 | 0.673538284 |
| 329 | NFE2L2 | ALDH3A1 | 0.603415229 |
| 330 | DLST | DDX5 | 0.568910017 |
| 331 | PDHA1 | DDX5 | 0.555233579 |
| 332 | SLC31A1 | DDX5 | 0.535658764 |
| 333 | FDX1 | DDX5 | 0.512097274 |
| 334 | DBT | DDX5 | 0.517643686 |
| 335 | LIPT1 | DDX5 | 0.594067492 |
| 336 | ATP7A | DDX5 | 0.547095872 |
| 337 | PDHB | DDX5 | 0.611336072 |
| 338 | MTF1 | DDX5 | 0.610634644 |
| 339 | DLST | PRKAR1A | 0.519525175 |
| 340 | SLC31A1 | PRKAR1A | 0.552062033 |
| 341 | FDX1 | PRKAR1A | 0.520509612 |
| 342 | DBT | PRKAR1A | 0.520371017 |
| 343 | DLAT | PRKAR1A | 0.521683826 |
| 344 | ATP7A | PRKAR1A | 0.639633224 |
| 345 | PDHB | PRKAR1A | 0.638202908 |
| 346 | MTF1 | PRKAR1A | 0.603562763 |
| 347 | NFE2L2 | CRYAB | 0.506351074 |
| 348 | DLD | HSPA8 | 0.663172103 |
| 349 | DLST | HSPA8 | 0.672521726 |
| 350 | SLC31A1 | HSPA8 | 0.646623125 |
| 351 | FDX1 | HSPA8 | 0.548575801 |
| 352 | DBT | HSPA8 | 0.578683475 |
| 353 | LIPT1 | HSPA8 | 0.510037757 |
| 354 | DLAT | HSPA8 | 0.751946659 |
| 355 | PDHB | HSPA8 | 0.61670934 |
| 356 | MTF1 | HSPA8 | 0.518771697 |
| 357 | DLD | PTGES3 | 0.69802093 |
| 358 | NFE2L2 | PTGES3 | 0.516146349 |
| 359 | DLST | PTGES3 | 0.647120509 |
| 360 | PDHA1 | PTGES3 | 0.63667564 |
| 361 | SLC31A1 | PTGES3 | 0.532765555 |
| 362 | FDX1 | PTGES3 | 0.53548615 |
| 363 | DBT | PTGES3 | 0.591280067 |
| 364 | LIPT1 | PTGES3 | 0.581521811 |
| 365 | DLAT | PTGES3 | 0.65274828 |
| 366 | PDHB | PTGES3 | 0.578851486 |
| 367 | LIPT2 | PTGES3 | 0.559819883 |
| 368 | DLD | GAPDH | 0.630899386 |
| 369 | NFE2L2 | GAPDH | 0.527127782 |
| 370 | PDHA1 | GAPDH | 0.615528543 |
| 371 | DLD | TPI1 | 0.660050608 |
| 372 | NFE2L2 | TPI1 | 0.510922839 |
| 373 | DLST | TPI1 | 0.554642442 |
| 374 | PDHA1 | TPI1 | 0.645601268 |
| 375 | DLAT | TPI1 | 0.532910832 |
| 376 | LIPT2 | TPI1 | 0.527290046 |
| 377 | NLRP3 | NEDD9 | 0.51608283 |
| 378 | DLD | TPD52L1 | 0.510334211 |
| 379 | NFE2L2 | TPD52L1 | 0.588238597 |
| 380 | PDHA1 | TPD52L1 | 0.50325497 |
| 381 | LIPT2 | TPD52L1 | 0.50183989 |
| 382 | DLD | SRSF3 | 0.698163704 |
| 383 | NFE2L2 | SRSF3 | 0.576334609 |
| 384 | DLST | SRSF3 | 0.696972788 |
| 385 | LIAS | SRSF3 | 0.562857782 |
| 386 | PDHA1 | SRSF3 | 0.684825878 |
| 387 | SLC31A1 | SRSF3 | 0.569156333 |
| 388 | FDX1 | SRSF3 | 0.579676758 |
| 389 | DBT | SRSF3 | 0.666423834 |
| 390 | LIPT1 | SRSF3 | 0.698353787 |
| 391 | DLAT | SRSF3 | 0.630721509 |
| 392 | PDHB | SRSF3 | 0.663560605 |
| 393 | LIPT2 | SRSF3 | 0.577394956 |
| 394 | NLRP3 | SOD2 | 0.52767044 |
| 395 | NLRP3 | CD83 | 0.616427148 |
| 396 | DLD | RPS12 | 0.572086533 |
| 397 | DLST | RPS12 | 0.605521029 |
| 398 | PDHA1 | RPS12 | 0.535157099 |
| 399 | FDX1 | RPS12 | 0.532741555 |
| 400 | LIPT1 | RPS12 | 0.529474788 |
| 401 | PDHB | RPS12 | 0.606830103 |
| 402 | DLST | C6orf62 | 0.604953909 |
| 403 | LIAS | C6orf62 | 0.508046216 |
| 404 | SLC31A1 | C6orf62 | 0.547700914 |
| 405 | FDX1 | C6orf62 | 0.535333427 |
| 406 | DBT | C6orf62 | 0.549275463 |
| 407 | LIPT1 | C6orf62 | 0.589878926 |
| 408 | DLAT | C6orf62 | 0.582670056 |
| 409 | ATP7A | C6orf62 | 0.505446023 |
| 410 | PDHB | C6orf62 | 0.644902339 |
| 411 | MTF1 | C6orf62 | 0.615215807 |
| 412 | DLD | PERP | 0.548675537 |
| 413 | NFE2L2 | PERP | 0.70271284 |
| 414 | PDHA1 | PERP | 0.506579605 |
| 415 | ATP7B | ARFGEF3 | 0.507540713 |
| 416 | DLD | HMGCS1 | 0.566259889 |
| 417 | NFE2L2 | HMGCS1 | 0.629729514 |
| 418 | PDHA1 | HMGCS1 | 0.501391112 |
| 419 | PDHB | ARRDC3 | 0.501952599 |
| 420 | NLRP3 | CPEB4 | 0.610657666 |
| 421 | ATP7A | CPEB4 | 0.583179744 |
| 422 | MTF1 | CPEB4 | 0.523022332 |
| 423 | DLD | FAM162A | 0.603699707 |
| 424 | NFE2L2 | FAM162A | 0.60461183 |
| 425 | PDHA1 | FAM162A | 0.609033924 |
| 426 | LIPT2 | FAM162A | 0.546193715 |
| 427 | DLD | RNF7 | 0.625899058 |
| 428 | NFE2L2 | RNF7 | 0.646122406 |
| 429 | DLST | RNF7 | 0.501480548 |
| 430 | PDHA1 | RNF7 | 0.629609886 |
| 431 | LIPT1 | RNF7 | 0.505822473 |
| 432 | LIPT2 | RNF7 | 0.584190982 |
| 433 | DLD | PDCD10 | 0.659475039 |
| 434 | NFE2L2 | PDCD10 | 0.679326471 |
| 435 | PDHA1 | PDCD10 | 0.544966824 |
| 436 | DBT | PDCD10 | 0.525408991 |
| 437 | LIPT2 | PDCD10 | 0.603299134 |
| 438 | NFE2L2 | COL7A1 | 0.528002515 |
| 439 | PDHA1 | HES1 | 0.518382962 |
| 440 | DLD | ABCC5 | 0.511304279 |
| 441 | NFE2L2 | ABCC5 | 0.746737956 |
| 442 | LIPT2 | ABCC5 | 0.541284742 |
| 443 | NFE2L2 | KLHL24 | 0.598649046 |
| 444 | DBT | KLHL24 | 0.544517588 |
| 445 | LIPT2 | KLHL24 | 0.52810827 |
| 446 | DLD | DGUOK | 0.652823804 |
| 447 | NFE2L2 | DGUOK | 0.531602155 |
| 448 | DLST | DGUOK | 0.565709697 |
| 449 | PDHA1 | DGUOK | 0.698424944 |
| 450 | GCSH | DGUOK | 0.5278694 |
| 451 | LIPT1 | DGUOK | 0.597802258 |
| 452 | DLAT | DGUOK | 0.524805101 |
| 453 | PDHB | DGUOK | 0.544594011 |
| 454 | LIPT2 | DGUOK | 0.60971934 |
| 455 | DLD | RPS15 | 0.504477827 |
| 456 | DLST | RPS15 | 0.530859347 |
| 457 | PDHA1 | RPS15 | 0.585729404 |
| 458 | PDHB | RPS15 | 0.553341067 |
| 459 | DLD | HTRA2 | 0.638694115 |
| 460 | DLST | HTRA2 | 0.684841837 |
| 461 | PDHA1 | HTRA2 | 0.665140884 |
| 462 | SLC31A1 | HTRA2 | 0.521237603 |
| 463 | DBT | HTRA2 | 0.542110057 |
| 464 | LIPT1 | HTRA2 | 0.585340438 |
| 465 | DLAT | HTRA2 | 0.624612334 |
| 466 | PDHB | HTRA2 | 0.635232315 |
| 467 | DLD | SRSF7 | 0.654422881 |
| 468 | NFE2L2 | SRSF7 | 0.540513496 |
| 469 | DLST | SRSF7 | 0.680273695 |
| 470 | LIAS | SRSF7 | 0.558393052 |
| 471 | PDHA1 | SRSF7 | 0.694983605 |
| 472 | SLC31A1 | SRSF7 | 0.538783173 |
| 473 | FDX1 | SRSF7 | 0.547949338 |
| 474 | DBT | SRSF7 | 0.590829536 |
| 475 | LIPT1 | SRSF7 | 0.700406639 |
| 476 | DLAT | SRSF7 | 0.580548667 |
| 477 | PDHB | SRSF7 | 0.646774055 |
| 478 | LIPT2 | SRSF7 | 0.529551191 |
| 479 | DLD | SFPQ | 0.609416522 |
| 480 | NFE2L2 | SFPQ | 0.519238803 |
| 481 | DLST | SFPQ | 0.577502114 |
| 482 | LIAS | SFPQ | 0.514474647 |
| 483 | PDHA1 | SFPQ | 0.704863266 |
| 484 | DBT | SFPQ | 0.590294423 |
| 485 | GCSH | SFPQ | 0.534462951 |
| 486 | LIPT1 | SFPQ | 0.532284294 |
| 487 | DLAT | SFPQ | 0.57511906 |
| 488 | MTF1 | SFPQ | 0.675160377 |
| 489 | DLD | MFN2 | 0.604595635 |
| 490 | NFE2L2 | MFN2 | 0.55180629 |
| 491 | DLST | MFN2 | 0.656600962 |
| 492 | PDHA1 | MFN2 | 0.67035173 |
| 493 | DBT | MFN2 | 0.60433866 |
| 494 | DLAT | MFN2 | 0.603940401 |
| 495 | MTF1 | MFN2 | 0.747428398 |
| 496 | DLD | SLC2A1 | 0.511543442 |
| 497 | NFE2L2 | SLC2A1 | 0.532515117 |
| 498 | DLD | PRDX1 | 0.640518934 |
| 499 | NFE2L2 | PRDX1 | 0.55024143 |
| 500 | PDHA1 | PRDX1 | 0.56967622 |
| 501 | DLAT | PRDX1 | 0.518268225 |
| 502 | LIPT2 | PRDX1 | 0.537699204 |
| 503 | LIPT1 | HMGN3 | 0.516600213 |
| 504 | NLRP3 | TNFAIP3 | 0.582438302 |
| 505 | DLD | SLIRP | 0.576757378 |
| 506 | DLST | SLIRP | 0.68225193 |
| 507 | PDHA1 | SLIRP | 0.666963598 |
| 508 | LIPT1 | SLIRP | 0.542264354 |
| 509 | PDHB | SLIRP | 0.537078117 |
| 510 | NLRP3 | DUSP1 | 0.577331939 |
| 511 | NLRP3 | CCDC170 | 0.631980064 |
| 512 | DLD | HSPH1 | 0.624589568 |
| 513 | NFE2L2 | HSPH1 | 0.569386555 |
| 514 | PDHA1 | HSPH1 | 0.588851367 |
| 515 | DBT | HSPH1 | 0.515119546 |
| 516 | DLAT | HSPH1 | 0.513285019 |
| 517 | NFE2L2 | CSTA | 0.694298562 |
| 518 | MTF1 | ODF2L | 0.500325409 |
| 519 | DLST | OPTN | 0.501508903 |
| 520 | NLRP3 | TREM1 | 0.54464823 |
| 521 | NLRP3 | LRRFIP1 | 0.522240508 |
| 522 | MTF1 | LRRFIP1 | 0.627938964 |
| 523 | NLRP3 | IRF1 | 0.533864028 |
| 524 | DLD | SNRPD2 | 0.576315799 |
| 525 | DLST | SNRPD2 | 0.54805619 |
| 526 | PDHA1 | SNRPD2 | 0.614846165 |
| 527 | LIPT1 | SNRPD2 | 0.521407271 |
| 528 | PDHB | SNRPD2 | 0.523736988 |
| 529 | DLD | RBCK1 | 0.53006357 |
| 530 | DLST | RBCK1 | 0.614189991 |
| 531 | PDHA1 | RBCK1 | 0.650400609 |
| 532 | LIPT1 | RBCK1 | 0.509124034 |
| 533 | PDHB | RBCK1 | 0.577961914 |
| 534 | DLD | SNRPB | 0.589096975 |
| 535 | DLST | SNRPB | 0.5670415 |
| 536 | PDHA1 | SNRPB | 0.674937074 |
| 537 | LIPT2 | SNRPB | 0.524781511 |
| 538 | DLD | EIF2S2 | 0.735526938 |
| 539 | NFE2L2 | EIF2S2 | 0.558058694 |
| 540 | DLST | EIF2S2 | 0.627259461 |
| 541 | LIAS | EIF2S2 | 0.513544268 |
| 542 | PDHA1 | EIF2S2 | 0.72881442 |
| 543 | SLC31A1 | EIF2S2 | 0.533600668 |
| 544 | FDX1 | EIF2S2 | 0.501204067 |
| 545 | DBT | EIF2S2 | 0.572257958 |
| 546 | LIPT1 | EIF2S2 | 0.53664174 |
| 547 | DLAT | EIF2S2 | 0.676852071 |
| 548 | PDHB | EIF2S2 | 0.562570671 |
| 549 | LIPT2 | EIF2S2 | 0.548397731 |
| 550 | DLD | COX6B1 | 0.545398157 |
| 551 | DLST | COX6B1 | 0.513690517 |
| 552 | PDHA1 | COX6B1 | 0.630710385 |
| 553 | LIPT1 | COX6B1 | 0.503176834 |
| 554 | PDHB | COX6B1 | 0.531718405 |
| 555 | LIPT2 | COX6B1 | 0.518019218 |
| 556 | ATP7A | AKAP9 | 0.600125868 |
| 557 | MTF1 | AKAP9 | 0.653671563 |
| 558 | NLRP3 | KDR | 0.596448752 |
| 559 | DLD | DGCR6L | 0.500157336 |
| 560 | DLST | DGCR6L | 0.560028664 |
| 561 | PDHA1 | DGCR6L | 0.602477237 |
| 562 | NFE2L2 | KRT17 | 0.595908861 |
| 563 | DLD | MRPS12 | 0.576704264 |
| 564 | PDHA1 | MRPS12 | 0.620689746 |
| 565 | LIPT2 | MRPS12 | 0.5188523 |
| 566 | NLRP3 | VPS13C | 0.538622656 |
| 567 | ATP7A | VPS13C | 0.600778794 |
| 568 | MTF1 | VPS13C | 0.565154835 |
| 569 | DLST | RPL36 | 0.52047751 |
| 570 | PDHA1 | RPL36 | 0.554867698 |
| 571 | LIPT1 | RPL36 | 0.507048563 |
| 572 | PDHB | RPL36 | 0.554574091 |
| 573 | DLD | PGLS | 0.51040114 |
| 574 | DLST | PGLS | 0.541850794 |
| 575 | PDHA1 | PGLS | 0.580808001 |
| 576 | PDHB | PGLS | 0.577007996 |
| 577 | DBT | RSPH3 | 0.519357404 |
| 578 | ATP7A | RSPH3 | 0.522578151 |
| 579 | MTF1 | RSPH3 | 0.635007947 |
| 580 | DLST | ATP5IF1 | 0.538369789 |
| 581 | LIAS | ATP5IF1 | 0.531257335 |
| 582 | PDHA1 | ATP5IF1 | 0.566780062 |
| 583 | FDX1 | ATP5IF1 | 0.544988436 |
| 584 | LIPT1 | ATP5IF1 | 0.557183197 |
| 585 | PDHB | ATP5IF1 | 0.629207326 |
| 586 | DLD | SLC6A8 | 0.526424117 |
| 587 | NFE2L2 | SLC6A8 | 0.639993035 |
| 588 | PDHA1 | SLC6A8 | 0.542986257 |
| 589 | LIPT2 | SLC6A8 | 0.508499325 |
| 590 | NFE2L2 | PNCK | 0.528230322 |
| 591 | NLRP3 | SYNE1 | 0.685311975 |
| 592 | DLD | COX4I1 | 0.52831898 |
| 593 | DLST | COX4I1 | 0.575580885 |
| 594 | PDHA1 | COX4I1 | 0.632848889 |
| 595 | FDX1 | COX4I1 | 0.554570682 |
| 596 | LIPT1 | COX4I1 | 0.51907029 |
| 597 | PDHB | COX4I1 | 0.607830355 |
| 598 | DLD | COX7B | 0.583301542 |
| 599 | DLST | COX7B | 0.525888319 |
| 600 | PDHA1 | COX7B | 0.651995077 |
| 601 | FDX1 | COX7B | 0.533735193 |
| 602 | LIPT1 | COX7B | 0.56660553 |
| 603 | PDHB | COX7B | 0.55490045 |
| 604 | LIPT2 | COX7B | 0.54955324 |
| 605 | DLD | RPL27 | 0.527438675 |
| 606 | DLST | RPL27 | 0.556021754 |
| 607 | FDX1 | RPL27 | 0.520706078 |
| 608 | LIPT1 | RPL27 | 0.596987244 |
| 609 | PDHB | RPL27 | 0.651250767 |
| 610 | DLD | DNAJB1 | 0.58942693 |
| 611 | NFE2L2 | DNAJB1 | 0.669700695 |
| 612 | DLST | DNAJB1 | 0.504766434 |
| 613 | PDHA1 | DNAJB1 | 0.524419806 |
| 614 | DLD | POMP | 0.60440697 |
| 615 | DLST | POMP | 0.609280958 |
| 616 | PDHA1 | POMP | 0.601025893 |
| 617 | SLC31A1 | POMP | 0.511832975 |
| 618 | FDX1 | POMP | 0.591146447 |
| 619 | LIPT1 | POMP | 0.582978379 |
| 620 | DLAT | POMP | 0.55561487 |
| 621 | PDHB | POMP | 0.652225286 |
| 622 | DLST | TCEAL4 | 0.557721085 |
| 623 | PDHA1 | TCEAL4 | 0.597207303 |
| 624 | PDHB | TCEAL4 | 0.544240285 |
| 625 | NFE2L2 | NTS | 0.599364908 |
| 626 | DLST | SARAF | 0.559876996 |
| 627 | SLC31A1 | SARAF | 0.558675458 |
| 628 | FDX1 | SARAF | 0.578251224 |
| 629 | PDHB | SARAF | 0.647545258 |
| 630 | FDX1 | RPS15A | 0.506717702 |
| 631 | LIPT1 | RPS15A | 0.59485602 |
| 632 | PDHB | RPS15A | 0.610960518 |
| 633 | NFE2L2 | DSG3 | 0.643425313 |
| 634 | NFE2L2 | ANXA1 | 0.509201909 |
| 635 | DLD | PRRG4 | 0.526086204 |
| 636 | NFE2L2 | PRRG4 | 0.67151027 |
| 637 | DLD | CD164 | 0.515085161 |
| 638 | DLST | CD164 | 0.603188531 |
| 639 | SLC31A1 | CD164 | 0.566737758 |
| 640 | FDX1 | CD164 | 0.610737596 |
| 641 | DBT | CD164 | 0.610560184 |
| 642 | LIPT1 | CD164 | 0.549969594 |
| 643 | DLAT | CD164 | 0.582293287 |
| 644 | PDHB | CD164 | 0.617044143 |
| 645 | NFE2L2 | GPNMB | 0.604647379 |
| 646 | DLD | NDUFB5 | 0.66649398 |
| 647 | NFE2L2 | NDUFB5 | 0.661538697 |
| 648 | PDHA1 | NDUFB5 | 0.574454905 |
| 649 | DBT | NDUFB5 | 0.526333419 |
| 650 | LIPT1 | NDUFB5 | 0.539285932 |
| 651 | LIPT2 | NDUFB5 | 0.61435244 |
| 652 | DLD | MRPL47 | 0.646544517 |
| 653 | NFE2L2 | MRPL47 | 0.646239943 |
| 654 | PDHA1 | MRPL47 | 0.667005043 |
| 655 | LIPT2 | MRPL47 | 0.616394066 |
| 656 | DLD | TRA2B | 0.69035366 |
| 657 | NFE2L2 | TRA2B | 0.721593166 |
| 658 | DLST | TRA2B | 0.548742176 |
| 659 | PDHA1 | TRA2B | 0.681554804 |
| 660 | DBT | TRA2B | 0.595631453 |
| 661 | GCSH | TRA2B | 0.513281667 |
| 662 | LIPT1 | TRA2B | 0.5081873 |
| 663 | DLAT | TRA2B | 0.539877038 |
| 664 | LIPT2 | TRA2B | 0.623098638 |
| 665 | DLD | TXN | 0.584507595 |
| 666 | NFE2L2 | TXN | 0.5418436 |
| 667 | PDHA1 | TXN | 0.564275875 |
| 668 | DLST | RPL35 | 0.51681841 |
| 669 | PDHA1 | RPL35 | 0.533739426 |
| 670 | LIPT1 | RPL35 | 0.500427359 |
| 671 | PDHB | RPL35 | 0.545924728 |
| 672 | DLST | RPS6 | 0.531171154 |
| 673 | LIPT1 | RPS6 | 0.562983862 |
| 674 | PDHB | RPS6 | 0.637969315 |
| 675 | DLD | RPLP1 | 0.54268699 |
| 676 | DLST | RPLP1 | 0.553021872 |
| 677 | PDHA1 | RPLP1 | 0.591423547 |
| 678 | PDHB | RPLP1 | 0.52771026 |
| 679 | DLD | KIF21A | 0.579775286 |
| 680 | NFE2L2 | KIF21A | 0.563440409 |
| 681 | PDHA1 | KIF21A | 0.510164985 |
| 682 | DLAT | KIF21A | 0.505452287 |
| 683 | NFE2L2 | DNAJA4 | 0.535151549 |
| 684 | ATP7A | ABHD2 | 0.585224113 |
| 685 | MTF1 | ABHD2 | 0.562158402 |
| 686 | DLD | RPS2 | 0.529098182 |
| 687 | DLST | RPS2 | 0.570318213 |
| 688 | PDHA1 | RPS2 | 0.620720915 |
| 689 | PDHB | RPS2 | 0.563240281 |
| 690 | PDHB | TOB1 | 0.541088231 |
| 691 | PDHB | IFITM3 | 0.530267487 |
| 692 | DLD | PGD | 0.628551308 |
| 693 | NFE2L2 | PGD | 0.571690493 |
| 694 | PDHA1 | PGD | 0.55682628 |
| 695 | LIPT2 | PGD | 0.530472713 |
| 696 | DLST | SH3BGRL3 | 0.531472787 |
| 697 | DLST | RPL11 | 0.562709373 |
| 698 | PDHA1 | RPL11 | 0.539950921 |
| 699 | FDX1 | RPL11 | 0.53395003 |
| 700 | LIPT1 | RPL11 | 0.533127452 |
| 701 | PDHB | RPL11 | 0.674110141 |
| 702 | DLST | RPS8 | 0.563197573 |
| 703 | LIAS | RPS8 | 0.525340069 |
| 704 | FDX1 | RPS8 | 0.508695572 |
| 705 | DBT | RPS8 | 0.506386069 |
| 706 | LIPT1 | RPS8 | 0.600988119 |
| 707 | PDHB | RPS8 | 0.65906202 |
| 708 | DLD | PFDN2 | 0.564934272 |
| 709 | DLST | PFDN2 | 0.517069404 |
| 710 | PDHA1 | PFDN2 | 0.711663351 |
| 711 | PDHB | PFDN2 | 0.512282581 |
| 712 | DLST | MCL1 | 0.515801027 |
| 713 | SLC31A1 | MCL1 | 0.54919417 |
| 714 | NLRP3 | MCL1 | 0.572622123 |
| 715 | PDHB | MCL1 | 0.519657466 |
| 716 | MTF1 | MCL1 | 0.610524771 |
| 717 | DLD | PARP1 | 0.652270167 |
| 718 | DLST | PARP1 | 0.522093843 |
| 719 | PDHA1 | PARP1 | 0.68523654 |
| 720 | DBT | PARP1 | 0.540184175 |
| 721 | DLAT | PARP1 | 0.644376906 |
| 722 | LIPT2 | PARP1 | 0.507600091 |
| 723 | MTF1 | PARP1 | 0.508311483 |
| 724 | DLD | RPS27A | 0.634211342 |
| 725 | DLST | RPS27A | 0.587871673 |
| 726 | LIAS | RPS27A | 0.514389766 |
| 727 | PDHA1 | RPS27A | 0.616993635 |
| 728 | DBT | RPS27A | 0.502886938 |
| 729 | LIPT1 | RPS27A | 0.616621106 |
| 730 | DLAT | RPS27A | 0.504530269 |
| 731 | PDHB | RPS27A | 0.601185809 |
| 732 | DLD | SNRPG | 0.623764189 |
| 733 | DLST | SNRPG | 0.521472421 |
| 734 | PDHA1 | SNRPG | 0.62807867 |
| 735 | LIPT1 | SNRPG | 0.566733548 |
| 736 | DLAT | SNRPG | 0.508406457 |
| 737 | PDHB | SNRPG | 0.509638389 |
| 738 | LIPT2 | SNRPG | 0.586394477 |
| 739 | DLAT | SLC20A1 | 0.504783065 |
| 740 | NLRP3 | CSRNP1 | 0.64338745 |
| 741 | DLST | RPL32 | 0.570855496 |
| 742 | FDX1 | RPL32 | 0.523540292 |
| 743 | LIPT1 | RPL32 | 0.579779402 |
| 744 | PDHB | RPL32 | 0.728767418 |
| 745 | DLD | RPL37 | 0.516318699 |
| 746 | PDHA1 | RPL37 | 0.587726266 |
| 747 | PDHB | RPL37 | 0.528400198 |
| 748 | DBT | PHIP | 0.630086754 |
| 749 | DLAT | PHIP | 0.507621256 |
| 750 | ATP7A | PHIP | 0.58100407 |
| 751 | MTF1 | PHIP | 0.603370162 |
| 752 | NLRP3 | PNRC1 | 0.542007072 |
| 753 | PDHB | PNRC1 | 0.541976161 |
| 754 | SLC31A1 | ARHGAP18 | 0.504617077 |
| 755 | NLRP3 | ARHGAP18 | 0.764892086 |
| 756 | NFE2L2 | GPC3 | 0.513007635 |
| 757 | SLC31A1 | CETN2 | 0.51001528 |
| 758 | FDX1 | CETN2 | 0.542372304 |
| 759 | LIPT1 | CETN2 | 0.543237608 |
| 760 | PDHB | CETN2 | 0.590757071 |
| 761 | DLST | RPL10 | 0.641770888 |
| 762 | FDX1 | RPL10 | 0.527907262 |
| 763 | LIPT1 | RPL10 | 0.55726504 |
| 764 | PDHB | RPL10 | 0.686301691 |
| 765 | DLD | RPL7 | 0.562581054 |
| 766 | DLST | RPL7 | 0.625778387 |
| 767 | LIAS | RPL7 | 0.513565617 |
| 768 | PDHA1 | RPL7 | 0.601444221 |
| 769 | DLAT | RPL7 | 0.532484594 |
| 770 | PDHB | RPL7 | 0.628253062 |
| 771 | NFE2L2 | NTRK2 | 0.721484438 |
| 772 | SLC31A1 | UGCG | 0.641007928 |
| 773 | DLD | RPL7A | 0.541927923 |
| 774 | DLST | RPL7A | 0.60276455 |
| 775 | PDHA1 | RPL7A | 0.550995937 |
| 776 | SLC31A1 | RPL7A | 0.532422366 |
| 777 | LIPT1 | RPL7A | 0.529607874 |
| 778 | PDHB | RPL7A | 0.649567642 |
| 779 | NFE2L2 | ADM | 0.580892244 |
| 780 | DLD | CELF1 | 0.5163873 |
| 781 | DLST | CELF1 | 0.510546188 |
| 782 | PDHA1 | CELF1 | 0.636130955 |
| 783 | DBT | CELF1 | 0.501587613 |
| 784 | DLAT | CELF1 | 0.543554276 |
| 785 | ATP7A | CELF1 | 0.532507635 |
| 786 | MTF1 | CELF1 | 0.708763696 |
| 787 | DLST | RPS3 | 0.588240241 |
| 788 | FDX1 | RPS3 | 0.521982417 |
| 789 | LIPT1 | RPS3 | 0.547806125 |
| 790 | DLAT | RPS3 | 0.52053322 |
| 791 | PDHB | RPS3 | 0.62494647 |
| 792 | DLD | ALDOA | 0.539984282 |
| 793 | PDHA1 | ALDOA | 0.584060219 |
| 794 | DLAT | ALDOA | 0.519947236 |
| 795 | NFE2L2 | AKR1C2 | 0.506803115 |
| 796 | DLD | BAG3 | 0.525484893 |
| 797 | NFE2L2 | BAG3 | 0.549316939 |
| 798 | DLST | BAG3 | 0.541010503 |
| 799 | PDHA1 | BAG3 | 0.580936595 |
| 800 | DLD | MZT2B | 0.516906292 |
| 801 | PDHA1 | MZT2B | 0.656425136 |
| 802 | NLRP3 | ZFP36L2 | 0.613940502 |
| 803 | MTF1 | ZFP36L2 | 0.602690898 |
| 804 | DLD | HNRNPU | 0.626111287 |
| 805 | DLST | HNRNPU | 0.562096483 |
| 806 | PDHA1 | HNRNPU | 0.677814711 |
| 807 | DBT | HNRNPU | 0.588557822 |
| 808 | GCSH | HNRNPU | 0.503529272 |
| 809 | DLAT | HNRNPU | 0.653145455 |
| 810 | ATP7A | HNRNPU | 0.590338127 |
| 811 | PDHB | HNRNPU | 0.535245592 |
| 812 | MTF1 | HNRNPU | 0.672333213 |
| 813 | DLD | MSI2 | 0.502622582 |
| 814 | LIPT2 | MSI2 | 0.508690405 |
| 815 | DBT | CXADR | 0.564205122 |
| 816 | DLST | KLF10 | 0.564150011 |
| 817 | MTF1 | KLF10 | 0.55974565 |
| 818 | DLD | AZIN1 | 0.642161704 |
| 819 | DLST | AZIN1 | 0.620469879 |
| 820 | PDHA1 | AZIN1 | 0.523895214 |
| 821 | SLC31A1 | AZIN1 | 0.557623505 |
| 822 | DBT | AZIN1 | 0.594238294 |
| 823 | LIPT1 | AZIN1 | 0.511433935 |
| 824 | DLAT | AZIN1 | 0.673060301 |
| 825 | PDHB | AZIN1 | 0.520153435 |
| 826 | SLC31A1 | UBE2L6 | 0.517549109 |
| 827 | MTF1 | RAB11FIP1 | 0.510199292 |
| 828 | DLD | MRPL17 | 0.565759816 |
| 829 | DLST | MRPL17 | 0.587178498 |
| 830 | PDHA1 | MRPL17 | 0.63186915 |
| 831 | LIPT1 | MRPL17 | 0.55718283 |
| 832 | DLAT | MRPL17 | 0.550229255 |
| 833 | PDHB | MRPL17 | 0.637534188 |
| 834 | DLST | PINK1 | 0.521655885 |
| 835 | NLRP3 | PINK1 | 0.542626957 |
| 836 | PDHB | PINK1 | 0.558999455 |
| 837 | MTF1 | PINK1 | 0.598794992 |
| 838 | SLC31A1 | SON | 0.510382548 |
| 839 | DBT | SON | 0.58387627 |
| 840 | DLAT | SON | 0.571025674 |
| 841 | ATP7A | SON | 0.680523915 |
| 842 | MTF1 | SON | 0.796224005 |
| 843 | DLD | HK2 | 0.511776853 |
| 844 | NFE2L2 | HK2 | 0.568229839 |
| 845 | DLD | G6PD | 0.556437191 |
| 846 | PDHA1 | G6PD | 0.531911405 |
| 847 | DLD | CSTB | 0.517248329 |
| 848 | NFE2L2 | CSTB | 0.501116848 |
| 849 | PDHA1 | CSTB | 0.527808092 |
| 850 | DLST | RPL8 | 0.603032169 |
| 851 | PDHA1 | RPL8 | 0.565402524 |
| 852 | PDHB | RPL8 | 0.59656802 |
| 853 | DLD | YDJC | 0.547500837 |
| 854 | PDHA1 | YDJC | 0.660777502 |
| 855 | LIPT2 | YDJC | 0.531180948 |
| 856 | PDHB | RPL26 | 0.58035576 |
| 857 | DLST | RPL29 | 0.577074713 |
| 858 | FDX1 | RPL29 | 0.500031934 |
| 859 | LIPT1 | RPL29 | 0.521480785 |
| 860 | PDHB | RPL29 | 0.744614022 |
| 861 | DLAT | ZNF281 | 0.554489572 |
| 862 | ATP7A | ZNF281 | 0.516845317 |
| 863 | PDHB | ZNF281 | 0.561392399 |
| 864 | MTF1 | CAPN2 | 0.537520692 |
| 865 | DLD | BOLA3 | 0.626934098 |
| 866 | DLST | BOLA3 | 0.509746574 |
| 867 | PDHA1 | BOLA3 | 0.664560917 |
| 868 | DLAT | BOLA3 | 0.52615882 |
| 869 | LIPT2 | BOLA3 | 0.559156216 |
| 870 | DLD | S100A11 | 0.536529726 |
| 871 | NFE2L2 | S100A11 | 0.556676777 |
| 872 | PDHA1 | S100A11 | 0.605163284 |
| 873 | NFE2L2 | DAPL1 | 0.646563188 |
| 874 | SLC31A1 | HIPK1 | 0.531496222 |
| 875 | DBT | HIPK1 | 0.623069529 |
| 876 | DLAT | HIPK1 | 0.506861806 |
| 877 | NLRP3 | HIPK1 | 0.536501695 |
| 878 | ATP7A | HIPK1 | 0.644647255 |
| 879 | MTF1 | HIPK1 | 0.780612216 |
| 880 | NFE2L2 | IFI16 | 0.612308904 |
| 881 | DLD | RPL22L1 | 0.513997593 |
| 882 | PDHA1 | RPL22L1 | 0.502482207 |
| 883 | ATP7A | CDS1 | 0.514107231 |
| 884 | MTF1 | CDS1 | 0.504104177 |
| 885 | DLD | YEATS2 | 0.597760897 |
| 886 | NFE2L2 | YEATS2 | 0.685317886 |
| 887 | PDHA1 | YEATS2 | 0.586408405 |
| 888 | LIPT2 | YEATS2 | 0.53840355 |
| 889 | DLD | TKT | 0.509580716 |
| 890 | PDHA1 | TKT | 0.529356456 |
| 891 | PDHB | TKT | 0.628146747 |
| 892 | NLRP3 | SGMS2 | 0.61944863 |
| 893 | DLST | TRA2A | 0.526372669 |
| 894 | LIAS | TRA2A | 0.583260476 |
| 895 | DBT | TRA2A | 0.511813277 |
| 896 | LIPT1 | TRA2A | 0.598128656 |
| 897 | PDHB | TRA2A | 0.576309509 |
| 898 | DLST | RPS14 | 0.563199751 |
| 899 | FDX1 | RPS14 | 0.534907043 |
| 900 | LIPT1 | RPS14 | 0.574667254 |
| 901 | PDHB | RPS14 | 0.698655665 |
| 902 | NFE2L2 | ALDH1A1 | 0.511796736 |
| 903 | PDHB | SPTSSA | 0.500074368 |
| 904 | NLRP3 | RRAD | 0.556504498 |
| 905 | SLC31A1 | B2M | 0.515458086 |
| 906 | NLRP3 | B2M | 0.633006402 |
| 907 | MTF1 | MIDN | 0.585288433 |
| 908 | DLST | RPL13 | 0.556390878 |
| 909 | PDHA1 | RPL13 | 0.521256019 |
| 910 | FDX1 | RPL13 | 0.500017705 |
| 911 | PDHB | RPL13 | 0.623324108 |
| 912 | DLD | EEF2 | 0.551983261 |
| 913 | DLST | EEF2 | 0.59789935 |
| 914 | PDHA1 | EEF2 | 0.568272083 |
| 915 | SLC31A1 | EEF2 | 0.500880295 |
| 916 | DBT | EEF2 | 0.511050344 |
| 917 | DLAT | EEF2 | 0.539204839 |
| 918 | PDHB | EEF2 | 0.586402237 |
| 919 | DLD | COPS6 | 0.747842164 |
| 920 | DLST | COPS6 | 0.613945501 |
| 921 | PDHA1 | COPS6 | 0.7070512 |
| 922 | LIPT1 | COPS6 | 0.529232013 |
| 923 | DLAT | COPS6 | 0.570913051 |
| 924 | PDHB | COPS6 | 0.606821494 |
| 925 | LIPT2 | COPS6 | 0.527854641 |
| 926 | DLST | ARF4 | 0.52069755 |
| 927 | SLC31A1 | ARF4 | 0.597608898 |
| 928 | FDX1 | ARF4 | 0.563350227 |
| 929 | DLAT | ARF4 | 0.565138697 |
| 930 | PDHB | ARF4 | 0.739895957 |
| 931 | DLST | STAT3 | 0.557501105 |
| 932 | SLC31A1 | STAT3 | 0.558983477 |
| 933 | DBT | STAT3 | 0.554707829 |
| 934 | DLAT | STAT3 | 0.541230598 |
| 935 | ATP7A | STAT3 | 0.589328646 |
| 936 | PDHB | STAT3 | 0.598925163 |
| 937 | MTF1 | STAT3 | 0.680473496 |
| 938 | DLST | SLC25A6 | 0.577688791 |
| 939 | PDHA1 | SLC25A6 | 0.66188824 |
| 940 | PDHB | SLC25A6 | 0.582240436 |
| 941 | DLD | KIF5B | 0.550699596 |
| 942 | DLST | KIF5B | 0.574151222 |
| 943 | SLC31A1 | KIF5B | 0.573913675 |
| 944 | DBT | KIF5B | 0.583879234 |
| 945 | DLAT | KIF5B | 0.662386394 |
| 946 | ATP7A | KIF5B | 0.594959427 |
| 947 | PDHB | KIF5B | 0.606616798 |
| 948 | MTF1 | KIF5B | 0.661622557 |
| 949 | PDHA1 | C9orf16 | 0.57029689 |
| 950 | PDHA1 | SCAND1 | 0.563622153 |
| 951 | PDHB | SCAND1 | 0.523120932 |
| 952 | DLD | PGAM1 | 0.656916126 |
| 953 | NFE2L2 | PGAM1 | 0.564633642 |
| 954 | DLST | PGAM1 | 0.629138394 |
| 955 | PDHA1 | PGAM1 | 0.600417346 |
| 956 | DLAT | PGAM1 | 0.591140146 |
| 957 | NFE2L2 | KRT19 | 0.513730403 |
| 958 | DLD | RPS7 | 0.550180301 |
| 959 | DLST | RPS7 | 0.531663955 |
| 960 | PDHA1 | RPS7 | 0.557106334 |
| 961 | LIPT1 | RPS7 | 0.582521091 |
| 962 | PDHB | RPS7 | 0.565959395 |
| 963 | LIPT2 | RPS7 | 0.506197549 |
| 964 | NLRP3 | JMJD1C | 0.558152676 |
| 965 | ATP7A | JMJD1C | 0.580929662 |
| 966 | MTF1 | JMJD1C | 0.635724172 |
| 967 | DLST | BSG | 0.602520578 |
| 968 | PDHA1 | BSG | 0.609267457 |
| 969 | PDHB | BSG | 0.597077663 |
| 970 | DLD | RPL38 | 0.538634147 |
| 971 | DLST | RPL38 | 0.519496591 |
| 972 | PDHA1 | RPL38 | 0.587328285 |
| 973 | LIPT1 | RPL38 | 0.580698587 |
| 974 | PDHB | RPL38 | 0.618496436 |
| 975 | DLD | MZT2A | 0.528490871 |
| 976 | PDHA1 | MZT2A | 0.66039281 |
| 977 | NLRP3 | TRIB1 | 0.524265475 |
| 978 | DLST | NUDT4 | 0.513072558 |
| 979 | DBT | NUDT4 | 0.567814463 |
| 980 | DLAT | NUDT4 | 0.514728386 |
| 981 | MTF1 | NUDT4 | 0.530098513 |
| 982 | DLD | UQCRH | 0.614558537 |
| 983 | DLST | UQCRH | 0.533391391 |
| 984 | PDHA1 | UQCRH | 0.659346108 |
| 985 | DBT | UQCRH | 0.501202353 |
| 986 | LIPT1 | UQCRH | 0.53004116 |
| 987 | DLAT | UQCRH | 0.53712321 |
| 988 | PDHB | UQCRH | 0.524245064 |
| 989 | LIPT2 | UQCRH | 0.512122154 |
| 990 | NLRP3 | PLK3 | 0.606015341 |
| 991 | MTF1 | PLK3 | 0.524143954 |
| 992 | FDX1 | FAM174A | 0.508244039 |
| 993 | PDHB | FAM174A | 0.579742167 |
| 994 | DLD | ATP2A2 | 0.687652159 |
| 995 | NFE2L2 | ATP2A2 | 0.578910508 |
| 996 | DLST | ATP2A2 | 0.558338568 |
| 997 | PDHA1 | ATP2A2 | 0.657737526 |
| 998 | DBT | ATP2A2 | 0.515522052 |
| 999 | DLAT | ATP2A2 | 0.655936441 |
| 1000 | MTF1 | ATP2A2 | 0.596609381 |
| 1001 | DLST | RPL15 | 0.610692018 |
| 1002 | LIAS | RPL15 | 0.513338922 |
| 1003 | SLC31A1 | RPL15 | 0.520897398 |
| 1004 | FDX1 | RPL15 | 0.602851404 |
| 1005 | DBT | RPL15 | 0.545822143 |
| 1006 | LIPT1 | RPL15 | 0.626711723 |
| 1007 | PDHB | RPL15 | 0.787126663 |
| 1008 | DLD | PARL | 0.566272171 |
| 1009 | NFE2L2 | PARL | 0.604781122 |
| 1010 | PDHA1 | PARL | 0.636074876 |
| 1011 | GCSH | PARL | 0.508034797 |
| 1012 | LIPT2 | PARL | 0.607400642 |
| 1013 | NFE2L2 | SFN | 0.587147819 |
| 1014 | PDHA1 | SFN | 0.503398916 |
| 1015 | DLD | COX8A | 0.61361524 |
| 1016 | DLST | COX8A | 0.617635492 |
| 1017 | PDHA1 | COX8A | 0.699867359 |
| 1018 | FDX1 | COX8A | 0.545919965 |
| 1019 | LIPT1 | COX8A | 0.534995084 |
| 1020 | DLAT | COX8A | 0.546435497 |
| 1021 | PDHB | COX8A | 0.616231759 |
| 1022 | LIPT2 | COX8A | 0.550715138 |
| 1023 | DLD | TALDO1 | 0.596425013 |
| 1024 | NFE2L2 | TALDO1 | 0.515570959 |
| 1025 | PDHA1 | TALDO1 | 0.578331277 |
| 1026 | LIPT2 | TALDO1 | 0.529327703 |
| 1027 | DLD | GBA | 0.501461376 |
| 1028 | DLST | GBA | 0.612740106 |
| 1029 | PDHA1 | GBA | 0.516351332 |
| 1030 | SLC31A1 | GBA | 0.563358186 |
| 1031 | DLAT | GBA | 0.581658695 |
| 1032 | PDHB | GBA | 0.642209084 |
| 1033 | DLD | RPS27 | 0.501732756 |
| 1034 | DLST | RPS27 | 0.562185438 |
| 1035 | FDX1 | RPS27 | 0.520534871 |
| 1036 | LIPT1 | RPS27 | 0.630954203 |
| 1037 | PDHB | RPS27 | 0.686770162 |
| 1038 | GLS | KCTD12 | 0.504310602 |
| 1039 | NLRP3 | KCTD12 | 0.758908477 |
| 1040 | DLD | EIF3K | 0.570411818 |
| 1041 | DLST | EIF3K | 0.559870467 |
| 1042 | PDHA1 | EIF3K | 0.612400097 |
| 1043 | LIPT1 | EIF3K | 0.504470434 |
| 1044 | PDHB | EIF3K | 0.515588542 |
| 1045 | DLD | NPM1 | 0.598575668 |
| 1046 | DLST | NPM1 | 0.593349372 |
| 1047 | LIAS | NPM1 | 0.557733464 |
| 1048 | PDHA1 | NPM1 | 0.623267141 |
| 1049 | DBT | NPM1 | 0.529634523 |
| 1050 | LIPT1 | NPM1 | 0.54936945 |
| 1051 | DLAT | NPM1 | 0.586908884 |
| 1052 | PDHB | NPM1 | 0.634441868 |
| 1053 | PDHA1 | RPS17 | 0.515415715 |
| 1054 | GCSH | RPS17 | 0.579161752 |
| 1055 | DLD | RPL35A | 0.588916137 |
| 1056 | NFE2L2 | RPL35A | 0.559373997 |
| 1057 | PDHA1 | RPL35A | 0.601364703 |
| 1058 | LIPT1 | RPL35A | 0.526625594 |
| 1059 | LIPT2 | RPL35A | 0.529965498 |
| 1060 | PDHB | CIB1 | 0.537432323 |
| 1061 | DLD | MRPL40 | 0.567930141 |
| 1062 | DLST | MRPL40 | 0.574308925 |
| 1063 | LIAS | MRPL40 | 0.503445933 |
| 1064 | PDHA1 | MRPL40 | 0.620834191 |
| 1065 | FDX1 | MRPL40 | 0.505038766 |
| 1066 | DBT | MRPL40 | 0.523422854 |
| 1067 | LIPT1 | MRPL40 | 0.545570686 |
| 1068 | PDHB | MRPL40 | 0.568724912 |
| 1069 | LIPT2 | MRPL40 | 0.527051536 |
| 1070 | NFE2L2 | NDUFA4L2 | 0.527080267 |
| 1071 | MTF1 | ZFP36L1 | 0.522713355 |
| 1072 | DLD | UBE2L3 | 0.646086596 |
| 1073 | NFE2L2 | UBE2L3 | 0.586342596 |
| 1074 | DLST | UBE2L3 | 0.6096601 |
| 1075 | PDHA1 | UBE2L3 | 0.710010066 |
| 1076 | FDX1 | UBE2L3 | 0.502814524 |
| 1077 | DBT | UBE2L3 | 0.534139665 |
| 1078 | DLAT | UBE2L3 | 0.550232016 |
| 1079 | PDHB | UBE2L3 | 0.50926467 |
| 1080 | LIPT2 | UBE2L3 | 0.536969339 |
| 1081 | DLD | POLR1D | 0.619821962 |
| 1082 | NFE2L2 | POLR1D | 0.593711835 |
| 1083 | DLST | POLR1D | 0.632737185 |
| 1084 | LIAS | POLR1D | 0.511348416 |
| 1085 | PDHA1 | POLR1D | 0.620840041 |
| 1086 | FDX1 | POLR1D | 0.518491079 |
| 1087 | DBT | POLR1D | 0.574426764 |
| 1088 | LIPT1 | POLR1D | 0.571909379 |
| 1089 | DLAT | POLR1D | 0.52827874 |
| 1090 | PDHB | POLR1D | 0.572798982 |
| 1091 | LIPT2 | POLR1D | 0.559850188 |
| 1092 | DLST | RPS23 | 0.524186587 |
| 1093 | FDX1 | RPS23 | 0.52596608 |
| 1094 | LIPT1 | RPS23 | 0.510170223 |
| 1095 | PDHB | RPS23 | 0.658036153 |
| 1096 | DLD | INSIG1 | 0.519511476 |
| 1097 | SLC31A1 | INSIG1 | 0.525727884 |
| 1098 | DLD | UBE2H | 0.675128004 |
| 1099 | DLST | UBE2H | 0.513904054 |
| 1100 | PDHA1 | UBE2H | 0.550704399 |
| 1101 | DLAT | UBE2H | 0.541499701 |
| 1102 | ATP7A | UBE2H | 0.53579738 |
| 1103 | MTF1 | UBE2H | 0.594355476 |
| 1104 | PDHA1 | HEXIM1 | 0.528472645 |
| 1105 | MTF1 | HEXIM1 | 0.56534787 |
| 1106 | MTF1 | DYNC2H1 | 0.573299045 |
| 1107 | NFE2L2 | TPRG1 | 0.618327824 |
| 1108 | DLD | LAMTOR4 | 0.628008251 |
| 1109 | DLST | LAMTOR4 | 0.519207813 |
| 1110 | PDHA1 | LAMTOR4 | 0.576891967 |
| 1111 | LIPT1 | LAMTOR4 | 0.515017035 |
| 1112 | PDHB | LAMTOR4 | 0.516617116 |
| 1113 | LIPT2 | LAMTOR4 | 0.534804205 |
| 1114 | DLST | RPL14 | 0.553249188 |
| 1115 | LIAS | RPL14 | 0.500029779 |
| 1116 | FDX1 | RPL14 | 0.559105887 |
| 1117 | LIPT1 | RPL14 | 0.542782621 |
| 1118 | PDHB | RPL14 | 0.778232485 |
| 1119 | NLRP3 | LRRK2 | 0.561136429 |
| 1120 | MTF1 | CFAP126 | 0.553868115 |
| 1121 | DLD | PPIA | 0.611654734 |
| 1122 | DLST | PPIA | 0.590153898 |
| 1123 | LIAS | PPIA | 0.513612141 |
| 1124 | PDHA1 | PPIA | 0.639199203 |
| 1125 | LIPT1 | PPIA | 0.52642037 |
| 1126 | DLAT | PPIA | 0.520164694 |
| 1127 | PDHB | PPIA | 0.589335972 |
| 1128 | DLD | NACA | 0.608582185 |
| 1129 | DLST | NACA | 0.619747901 |
| 1130 | LIAS | NACA | 0.524102415 |
| 1131 | PDHA1 | NACA | 0.704721818 |
| 1132 | FDX1 | NACA | 0.555639333 |
| 1133 | DBT | NACA | 0.534079006 |
| 1134 | LIPT1 | NACA | 0.580639245 |
| 1135 | DLAT | NACA | 0.559947835 |
| 1136 | PDHB | NACA | 0.655962494 |
| 1137 | LIPT2 | NACA | 0.502644919 |
| 1138 | DLD | TOMM7 | 0.518078712 |
| 1139 | DLST | TOMM7 | 0.545681415 |
| 1140 | PDHA1 | TOMM7 | 0.525439826 |
| 1141 | FDX1 | TOMM7 | 0.530332411 |
| 1142 | LIPT1 | TOMM7 | 0.548054731 |
| 1143 | PDHB | TOMM7 | 0.591342809 |
| 1144 | NFE2L2 | S100A2 | 0.540032867 |
| 1145 | DLD | SRC | 0.550867033 |
| 1146 | DLST | SRC | 0.562727079 |
| 1147 | PDHA1 | SRC | 0.647197408 |
| 1148 | MTF1 | SRC | 0.516420086 |
| 1149 | ATP7B | NEK5 | 0.509030552 |
| 1150 | NLRP3 | SERPINA1 | 0.537247106 |
| 1151 | DLD | MRPL21 | 0.569091827 |
| 1152 | DLST | MRPL21 | 0.563876349 |
| 1153 | PDHA1 | MRPL21 | 0.665321019 |
| 1154 | LIPT1 | MRPL21 | 0.505934123 |
| 1155 | DLAT | MRPL21 | 0.529554682 |
| 1156 | PDHB | MRPL21 | 0.529214216 |
| 1157 | LIPT2 | MRPL21 | 0.5430896 |
| 1158 | DLST | RPL12 | 0.557450446 |
| 1159 | PDHA1 | RPL12 | 0.528203144 |
| 1160 | LIPT1 | RPL12 | 0.527060074 |
| 1161 | PDHB | RPL12 | 0.64277522 |
| 1162 | DLST | RPS4X | 0.564106477 |
| 1163 | PDHA1 | RPS4X | 0.53077225 |
| 1164 | FDX1 | RPS4X | 0.504147262 |
| 1165 | LIPT1 | RPS4X | 0.54347312 |
| 1166 | PDHB | RPS4X | 0.645909788 |
| 1167 | NFE2L2 | AKR1B10 | 0.508925652 |
| 1168 | DLD | CALM1 | 0.509361633 |
| 1169 | DLST | CALM1 | 0.695428163 |
| 1170 | SLC31A1 | CALM1 | 0.53732629 |
| 1171 | FDX1 | CALM1 | 0.526870783 |
| 1172 | DBT | CALM1 | 0.531301645 |
| 1173 | PDHB | CALM1 | 0.546470685 |
| 1174 | MTF1 | CALM1 | 0.561847228 |
| 1175 | DLD | RPL10A | 0.534122042 |
| 1176 | DLST | RPL10A | 0.648798981 |
| 1177 | LIAS | RPL10A | 0.531697966 |
| 1178 | PDHA1 | RPL10A | 0.510872725 |
| 1179 | FDX1 | RPL10A | 0.524665306 |
| 1180 | DBT | RPL10A | 0.559524486 |
| 1181 | LIPT1 | RPL10A | 0.6019533 |
| 1182 | DLAT | RPL10A | 0.543020271 |
| 1183 | PDHB | RPL10A | 0.693520634 |
| 1184 | DLST | RPL39 | 0.517920247 |
| 1185 | LIPT1 | RPL39 | 0.580645792 |
| 1186 | PDHB | RPL39 | 0.598841904 |
| 1187 | DLST | APRT | 0.596184297 |
| 1188 | PDHA1 | APRT | 0.598888471 |
| 1189 | DLAT | APRT | 0.500123948 |
| 1190 | PDHB | APRT | 0.606321663 |
| 1191 | DLD | BRD2 | 0.606060466 |
| 1192 | NFE2L2 | BRD2 | 0.529031006 |
| 1193 | DLST | BRD2 | 0.66441555 |
| 1194 | PDHA1 | BRD2 | 0.679126515 |
| 1195 | DBT | BRD2 | 0.591472586 |
| 1196 | DLAT | BRD2 | 0.621759344 |
| 1197 | PDHB | BRD2 | 0.561849942 |
| 1198 | MTF1 | BRD2 | 0.667529172 |
| 1199 | DLD | HSPA1B | 0.542542318 |
| 1200 | PDHA1 | HSPA1B | 0.543533523 |
| 1201 | NLRP3 | HLA-C | 0.567448433 |
| 1202 | DLST | RACK1 | 0.590080741 |
| 1203 | PDHA1 | RACK1 | 0.535092276 |
| 1204 | LIPT1 | RACK1 | 0.519009855 |
| 1205 | PDHB | RACK1 | 0.698780848 |
| 1206 | DLD | UQCC3 | 0.544807142 |
| 1207 | DLST | UQCC3 | 0.551607542 |
| 1208 | PDHA1 | UQCC3 | 0.645017839 |
| 1209 | GCSH | UQCC3 | 0.501796236 |
| 1210 | LIPT1 | UQCC3 | 0.526032793 |
| 1211 | DLAT | UQCC3 | 0.534843251 |
| 1212 | PDHB | UQCC3 | 0.512113403 |
| 1213 | LIPT2 | UQCC3 | 0.539558571 |
| 1214 | NFE2L2 | KRT6A | 0.600348403 |
| 1215 | DLD | DNAJC19 | 0.626009277 |
| 1216 | NFE2L2 | DNAJC19 | 0.672979788 |
| 1217 | PDHA1 | DNAJC19 | 0.617782781 |
| 1218 | DBT | DNAJC19 | 0.501443349 |
| 1219 | GCSH | DNAJC19 | 0.505982266 |
| 1220 | LIPT1 | DNAJC19 | 0.538012346 |
| 1221 | LIPT2 | DNAJC19 | 0.658527661 |
| 1222 | DLAT | ANKRD28 | 0.513606996 |
| 1223 | ATP7A | ANKRD28 | 0.603418143 |
| 1224 | PDHB | ANKRD28 | 0.536982076 |
| 1225 | MTF1 | ANKRD28 | 0.60212764 |
| 1226 | DLD | PHB2 | 0.639893196 |
| 1227 | DLST | PHB2 | 0.613486524 |
| 1228 | PDHA1 | PHB2 | 0.663417188 |
| 1229 | DLAT | PHB2 | 0.529735307 |
| 1230 | PDHB | PHB2 | 0.548596661 |
| 1231 | LIPT2 | PHB2 | 0.562471049 |
| 1232 | DLD | DDX3X | 0.567277734 |
| 1233 | DLST | DDX3X | 0.576863714 |
| 1234 | PDHA1 | DDX3X | 0.553874399 |
| 1235 | SLC31A1 | DDX3X | 0.558763182 |
| 1236 | FDX1 | DDX3X | 0.516371338 |
| 1237 | DBT | DDX3X | 0.602611885 |
| 1238 | DLAT | DDX3X | 0.612491664 |
| 1239 | ATP7A | DDX3X | 0.641776379 |
| 1240 | PDHB | DDX3X | 0.528138014 |
| 1241 | MTF1 | DDX3X | 0.744547383 |
| 1242 | DLD | UBA52 | 0.593478651 |
| 1243 | NFE2L2 | UBA52 | 0.511118177 |
| 1244 | DLST | UBA52 | 0.597136902 |
| 1245 | PDHA1 | UBA52 | 0.623240944 |
| 1246 | LIPT1 | UBA52 | 0.518211522 |
| 1247 | PDHB | UBA52 | 0.583362153 |
| 1248 | NLRP3 | PRR29 | 0.521517844 |
| 1249 | DLST | RPS18 | 0.541474304 |
| 1250 | LIPT1 | RPS18 | 0.573921498 |
| 1251 | PDHB | RPS18 | 0.632875116 |
| 1252 | DLST | RPS28 | 0.538242577 |
| 1253 | PDHA1 | RPS28 | 0.563657366 |
| 1254 | PDHB | RPS28 | 0.56268968 |
| 1255 | DLD | FAM133B | 0.676967592 |
| 1256 | DLST | FAM133B | 0.582945926 |
| 1257 | LIAS | FAM133B | 0.538081977 |
| 1258 | PDHA1 | FAM133B | 0.594013958 |
| 1259 | DBT | FAM133B | 0.637316081 |
| 1260 | GCSH | FAM133B | 0.507697857 |
| 1261 | LIPT1 | FAM133B | 0.545354401 |
| 1262 | DLAT | FAM133B | 0.582393143 |
| 1263 | ATP7A | FAM133B | 0.513792501 |
| 1264 | MTF1 | FAM133B | 0.525045658 |
| 1265 | NLRP3 | HLA-B | 0.626150617 |
| 1266 | GCSH | RPL36A | 0.570654464 |
| 1267 | DLD | ARPC1A | 0.653194992 |
| 1268 | PDHA1 | ARPC1A | 0.598652752 |
| 1269 | GCSH | ARPC1A | 0.538095806 |
| 1270 | DLD | C22orf39 | 0.514638184 |
| 1271 | DLST | C22orf39 | 0.58115734 |
| 1272 | PDHA1 | C22orf39 | 0.525319773 |
| 1273 | FDX1 | C22orf39 | 0.534011049 |
| 1274 | DBT | C22orf39 | 0.570863822 |
| 1275 | LIPT1 | C22orf39 | 0.563791819 |
| 1276 | PDHB | C22orf39 | 0.578890376 |
| 1277 | DLD | EIF6 | 0.594248531 |
| 1278 | DLST | EIF6 | 0.646580852 |
| 1279 | PDHA1 | EIF6 | 0.675867009 |
| 1280 | DLAT | EIF6 | 0.578394749 |
| 1281 | PDHB | EIF6 | 0.551404709 |
| 1282 | NLRP3 | HBB | 0.505313702 |
| 1283 | DLST | MRPS21 | 0.506579558 |
| 1284 | PDHA1 | MRPS21 | 0.575575965 |
| 1285 | LIPT1 | MRPS21 | 0.56520654 |
| 1286 | PDHB | MRPS21 | 0.605346105 |
| 1287 | DLD | MARCKS | 0.550657419 |
| 1288 | NFE2L2 | MARCKS | 0.565912395 |

Supplementary Table 4. Univariate Cox regression analysis screening for cuproptosis-associated mitochondrial depolarization genes. Based on the results of the univariate Cox proportional risk model, the mitochondrial depolarization genes associated with cuproptosis were listed and the risk ratio (HR), 95% confidence interval (CI), and p-value of each gene are shown for initial screening of potential prognostic markers.

| Characteristics | HR | p | CI |
| --- | --- | --- | --- |
| CD9 | 1.25 | 0.044 | 1.01 - 1.55 |
| DCN | 1.38 | 0.001 | 1.14 - 1.68 |
| RPL18 | 1.42 | 0.047 | 1.00 - 2.00 |
| GPC1 | 1.2 | 0.011 | 1.04 - 1.39 |
| IDI1 | 1.4 | 0.033 | 1.03 - 1.90 |
| KLF6 | 1.29 | 0.039 | 1.01 - 1.65 |
| ATP1B3 | 1.27 | 0.009 | 1.06 - 1.51 |
| PTHLH | 1.1 | 0.018 | 1.02 - 1.19 |
| PSMD8 | 1.55 | 0.011 | 1.11 - 2.16 |
| SLC25A1 | 1.38 | 0.04 | 1.02 - 1.89 |
| EIF5 | 1.51 | 0.024 | 1.05 - 2.15 |
| AHCY | 1.46 | 0.007 | 1.11 - 1.92 |
| PSMD7 | 1.51 | 0.02 | 1.07 - 2.13 |
| FBL | 1.3 | 0.049 | 1.00 - 1.69 |
| CRYAB | 1.24 | 0.004 | 1.07 - 1.44 |
| HSPA8 | 1.54 | 0.011 | 1.10 - 2.14 |
| GAPDH | 1.31 | 0.027 | 1.03 - 1.66 |
| PERP | 1.18 | 0.047 | 1.00 - 1.39 |
| HMGCS1 | 1.31 | 0.005 | 1.08 - 1.59 |
| RNF7 | 1.41 | 0.026 | 1.04 - 1.91 |
| PDCD10 | 1.29 | 0.045 | 1.01 - 1.66 |
| MFN2 | 1.48 | 0.031 | 1.04 - 2.12 |
| SLC2A1 | 1.16 | 0.038 | 1.01 - 1.34 |
| CSTA | 1.11 | 0.027 | 1.01 - 1.23 |
| SNRPD2 | 1.43 | 0.017 | 1.07 - 1.93 |
| KRT17 | 1.09 | 0.025 | 1.01 - 1.18 |
| MRPS12 | 1.34 | 0.035 | 1.02 - 1.76 |
| DSG3 | 1.06 | 0.012 | 1.01 - 1.12 |
| RPL7A | 1.38 | 0.044 | 1.01 - 1.89 |
| DLAT | 1.61 | 0.01 | 1.12 - 2.31 |
| ZFP36L2 | 1.43 | 0.004 | 1.12 - 1.83 |
| KLF10 | 1.44 | 0.018 | 1.06 - 1.95 |
| AZIN1 | 1.38 | 0.045 | 1.01 - 1.90 |
| UBE2L6 | 1.31 | 0.037 | 1.02 - 1.70 |
| NLRP3 | 1.26 | 0.022 | 1.03 - 1.53 |
| DAPL1 | 1.08 | 0.018 | 1.01 - 1.15 |
| EEF2 | 1.36 | 0.039 | 1.02 - 1.81 |
| SCAND1 | 1.37 | 0.042 | 1.01 - 1.85 |
| PGAM1 | 1.53 | 0.004 | 1.15 - 2.04 |
| NUDT4 | 1.83 | 0 | 1.32 - 2.56 |
| EIF3K | 1.56 | 0.013 | 1.1 - 2.21 |
| NPM1 | 1.4 | 0.037 | 1.02 - 1.93 |
| NDUFA4L2 | 1.17 | 0.012 | 1.03 - 1.31 |
| POLR1D | 1.55 | 0.015 | 1.09 - 2.21 |
| RPL10A | 1.53 | 0.013 | 1.09 - 2.13 |
| PHB2 | 1.43 | 0.033 | 1.03 - 1.99 |
| UBA52 | 1.46 | 0.033 | 1.03 - 2.06 |
| RPS28 | 1.36 | 0.046 | 1.01 - 1.85 |
| RPL36A | 0.8 | 0.047 | 0.65 – 1.00 |

Supplementary Table 5. Risk genes screened by Lasso regression analysis. The table includes the final selected genes and their coefficient estimates.

|  | Gene | Coef |
| --- | --- | --- |
| 1 | DCN | 0.090958619 |
| 2 | PTHLH | 0.031205741 |
| 3 | CRYAB | 0.038814322 |
| 4 | HMGCS1 | 0.080914645 |
| 5 | DSG3 | 0.000761663 |
| 6 | ZFP36L2 | 0.07224978 |
| 7 | SCAND1 | 0.074244289 |
| 8 | NUDT4 | 0.213496317 |
| 9 | NDUFA4L2 | 0.00750522 |
| 10 | RPL36A | -0.204396028 |

Supplementary Table 6. Results of the DGIdb drug database for predicting risk genes. The predictive effects of risk genes on patient prognosis are summarized and combined with information from the DGIdb database to explore whether these genes are known targets of drug action and provide a reference point for personalized treatment.

| gene | drug | regulatory approval | indication | interaction score |
| --- | --- | --- | --- | --- |
| DSG3 | COMPOUND 66 [PMID: 19788238] | Not Approved |  | 4.773424658 |
| PTHLH | VINBLASTINE | Approved | Antineoplastic Agents | 0.514781091 |
| PTHLH | PANCREATIC PROTEOLYTIC ENZYMES | Not Approved |  | 4.37563927 |
| PTHLH | MAGNESIUM SULFATE ANHYDROUS | Approved |  | 4.37563927 |
| PTHLH | DIMETHYL SULFOXIDE | Approved |  | 1.093909817 |
| PTHLH | CAL | Not Approved |  | 8.751278539 |
| PTHLH | DIFFERENTIATION INDUCER | Not Approved |  | 1.591141553 |
| ZFP36L2 | 3-METHYLHEXANAL | Not Approved |  | 3.750547945 |
| ZFP36L2 | ALLOIN | Not Approved |  | 3.750547945 |
| ZFP36L2 | GROSHEIMIN | Not Approved |  | 1.250182648 |
| ZFP36L2 | CYCLOLINOPEPTIDE 1-MSO,3-MET-CL6 | Not Approved |  | 3.750547945 |
| ZFP36L2 | CITRONELLAL | Not Approved |  | 3.750547945 |
| ZFP36L2 | SACCHARIN | Approved |  | 1.875273973 |
| ZFP36L2 | BENGALENSOL | Not Approved |  | 1.875273973 |
| ZFP36L2 | BUTYRIC ACID | Not Approved |  | 0.258658479 |
| ZFP36L2 | GIV3727 | Not Approved |  | 1.250182648 |
| ZFP36L2 | AMAROGENTIN | Not Approved |  | 1.250182648 |
| ZFP36L2 | LACTUCOPICRIN | Not Approved |  | 1.875273973 |
| ZFP36L2 | ARISTOLOCHIC ACID | Not Approved |  | 0.750109589 |
| ZFP36L2 | CYCLAMATE | Not Approved |  | 1.875273973 |
| ZFP36L2 | ACESULFAME | Approved |  | 1.875273973 |
| DCN | MARIMASTAT | Not Approved | Antineoplastic Agents | 0.500073059 |
| DCN | RECOMBINANT 70-KD HEAT-SHOCK PROTEIN | Not Approved |  | 1.500219178 |
| DCN | CM-352 | Not Approved |  | 3.750547945 |
| DCN | SIROLIMUS | Approved | for treatment of wet age-related macular degeneration,immunosuppressant | 0.263196347 |
| DCN | MMP13 TRACER [18F]5J | Not Approved |  | 2.500365297 |
| DCN | RECOMBINANT INTERFERON | Not Approved |  | 0.384671584 |
| DCN | ASCORBIC ACID | Approved |  | 0.577007376 |
| NDUFA4L2 | ME-344 | Not Approved |  | 0.343187394 |
| NDUFA4L2 | METFORMIN HYDROCHLORIDE | Approved |  | 0.312545662 |
| NDUFA4L2 | NV-128 | Not Approved |  | 0.350051142 |
